# Wilson Lines in the Abelian Lattice Higgs Model

**DOI:** 10.1007/s00220-024-05128-x

**Published:** 2024-11-05

**Authors:** Malin P. Forsström

**Affiliations:** Mathematical Sciences, Chalmers University of Technology and University of Gothenburg, 41296 Gothenburg, Sweden

## Abstract

Lattice gauge theories are lattice approximations of the Yang–Mills theory in physics. The abelian lattice Higgs model is one of the simplest examples of a lattice gauge theory interacting with an external field. In a previous paper (Forsström et al. in Math Phys 4(2):257–329, 2023), we calculated the leading order term of the expected value of Wilson loop observables in the low-temperature regime of the abelian lattice Higgs model on ${\mathbb {Z}}^4,$ with structure group $G = {\mathbb {Z}}_n$ for some $n \ge 2.$ In the absence of a Higgs field, these are important observables since they exhibit a phase transition which can be interpreted as distinguishing between regions with and without quark confinement. However, in the presence of a Higgs field, this is no longer the case, and a more relevant family of observables are so-called open Wilson lines. In this paper, we extend and refine the ideas introduced in Forsström et al. (Math Phys 4(2):257–329, 2023) to calculate the leading order term of the expected value of the more general Wilson line observables. Using our main result, we then calculate the leading order term of several natural ratios of expected values and confirm the behavior predicted by physicists.

## Introduction

### Background

Lattice gauge theories are spin models which describe the interaction of elementary particles. These were first introduced by Wilson [32] as lattice approximations of the quantum field theories that appear in the standard model, known as Yang-Mills theory. Since then, lattice gauge theories have been successfully used to understand the corresponding continuous models, and several of the predictions made using these lattice approximations have been verified experimentally. At about the same time as lattice gauge theories were introduced in the physics literature by Wilson, Wegner [31] introduced what he then called *generalized Ising models* as an example of a family of models with a phase transition without a local order parameter. In special cases, these generalized Ising models are lattice gauge theories, and as such, they have been used extensively as toy models for the lattice gauge theories that are more relevant for physics.

In the last couple of years, there has been a renewed interest in the rigorous analysis of four-dimensional lattice gauge theories in the mathematical community, see, e.g., [7–9, 16, 17, 21]. Most relevant for this work are the papers [7, 9, 17], in which the leading order term for the expectation of Wilson loop observables was computed for lattice gauge theories with Wilson action and finite structure groups.

Pure gauge theories model only the gauge field itself, and to advance towards physically relevant theories; it is necessary also to understand models that include external fields interacting with the gauge field, see, e.g., [19, 30]. In this paper, we consider a lattice gauge theory that models a gauge field coupled to a scalar Bosonic field with a quartic Higgs potential. The resulting model is called the *lattice Higgs model*. This model has received significant attention in the physics community. Some examples are the works [1–3], where calculations to obtain critical parameters of these models were performed, and [27, 28], in which phase diagrams were sketched. For further background, as well as more references, we refer the reader to [19] and [30].

In a recent paper [18], we extended the theory developed in [7, 9, 17] in order to describe the leading order term for the expectation of Wilson loop observables in the fixed length and low-temperature regime of the abelian Higgs model. Wilson loop expectations are natural observables in lattice gauge theories and were introduced by Wilson as a means to detect whether quark confinement occurs, see [32]. In lattice gauge theories without matter fields, one can show that the expected value of large Wilson loops undergo a phase transition, where it changes from following a so-called area law to following a so-called perimeter law. However, as discussed in, e.g., [29], in gauge theories with matter fields, the Wilson loop observable obeys a perimeter law for all parameters, and hence one cannot see a relevant phase transition using only the Wilson loop observable. For this reason, alternative observables have been suggested for studying the lattice Higgs model. One such observable is the open Wilson line observable, in which the loop in the Wilson loop observable is replaced by an open path that is saturated at the end-points by the Higgs field. This type of observable has been relatively well studied in the physics literature (see, e.g., [5, 6, 13, 15, 23, 24, 29, 30]). Moreover, the asymptotic behavior of such observables has been argued to be related to, e.g., the absence of bound states of the charged particle in the presence of an external source [13], confinement versus deconfinement in lattice gauge theories with matter fields [6], and binding versus unbinding of dynamical quarks in the field of a static color source [6]. Hence the Wilson line observables are of physical relevance.

### Preliminary notation

For $m \ge 2$, the graph naturally associated to ${\mathbb {Z}}^m$ has a vertex at each point $x \in {\mathbb {Z}}^m$ with integer coordinates and a non-oriented edge between nearest neighbors. We will work with oriented edges throughout this paper, and for this reason we associate to each non-oriented edge ${\bar{e}}$ two oriented edges $e_1$ and $e_2 = -e_1$ with the same endpoints as ${\bar{e}}$ and opposite orientations.

Let $d{\textbf{e}}_1 \, {:}{=}\, (1,0,0,\ldots ,0)$, $d{\textbf{e}}_2 \, {:}{=}\, (0,1,0,\ldots , 0)$, ..., $d{\textbf{e}}_m \, {:}{=}\, (0,\ldots ,0,1)$ be oriented edges corresponding to the unit vectors in ${\mathbb {Z}}^m$. We say that an oriented edge *e* is *positively oriented* if it is equal to a translation of one of these unit vectors, i.e., if there is a $v \in {\mathbb {Z}}^m$ and a $j \in \{ 1,2, \ldots , m\}$ such that $e = v + d{{\textbf{e}}}_j$. If $v \in {\mathbb {Z}}^m$ and $j_1 < j_2$, then $p = (v + d{\textbf{e}}_{j_1}) \wedge (v+ d{\textbf{e}}_{j_2})$ is a positively oriented 2-cell, also known as a *positively oriented plaquette*. We let $C_0({\mathbb {Z}}^4)$, $C_1({\mathbb {Z}}^4)$, and $C_2({\mathbb {Z}}^4)$ denote the sets of oriented vertices, edges, and plaquettes. Next, we let $B_N$ denote the set $[-N,N]^m \subseteq {\mathbb {Z}}^m$, and we let $C_0(B_N)$, $C_1(B_N)$, and $C_2(B_N)$ denote the sets of oriented vertices, edges, and plaquettes, respectively, whose end-points are all in $B_N$.

Whenever we talk about a lattice gauge theory we do so with respect to some (abelian) group $$(G,+) $$, referred to as the *structure group*. We also fix a unitary and faithful representation $$\rho $$ of $$(G,+)$$. In this paper, we will always assume that $$G = {\mathbb {Z}}_n$$ for some $$n \ge 2$$ with the group operation $$+$$ given by standard addition modulo *n*. Also, we will assume that $$\rho $$ is a one-dimensional representation of *G*. We note that a natural such representation is given by $$j\mapsto e^{j \cdot 2 \pi i/n}$$.

Now assume that a structure group $(G,+)$, a one-dimensional unitary representation $\rho$ of $(G,+)$, and an integer $N\ge 1$ are given. We let $\Omega ^1(B_N,G)$ denote the set of all *G*-valued 1-forms $\sigma$ on $C_1(B_N)$, i.e., the set of all *G*-valued functions $\sigma :e \mapsto \sigma (e)$ on $C_1(B_N)$ such that $\sigma (e) = -\sigma (-e)$ for all $e \in C_1(B_N)$. Similarly, we let $\Omega ^0(B_N,G)$ denote the set of all *G*-valued functions $\phi :x \mapsto \phi (x)$ on $C_0(B_N)$ which are such that $\phi (x) = - \phi (-x)$ for all $x \in C_1(B_N).$ When $\sigma \in \Omega ^1(B_N,G)$ and $p \in C_2(B_N)$, we let $\partial p$ denote the formal sum of the four edges $e_1,$ $e_2,$ $e_3,$ and $e_4$ in the oriented boundary of *p* (see Sect. 2.1.5), and define

$$\begin{aligned} d\sigma (p)\, {:}{=}\,\sigma (\partial p) \, {:}{=}\, \sum _{e \in \partial p} \sigma (e) \, {:}{=}\, \sigma (e_1) + \sigma (e_2) + \sigma (e_3) + \sigma (e_4). \end{aligned}$$

Similarly, when $\phi \in \Omega ^0(B_N,G)$ and $e \in C_1(B_N)$ is an edge from $x_1$ to $x_2$, we let $\partial e$ denote the formal sum $x_2-x_1,$ and define $d\phi (e) \, {:}{=}\, \phi (\partial e) \, {:}{=}\, \phi (x_2) - \phi (x_1).$

### The abelian lattice Higgs model

Given $\beta , \kappa ,\zeta \ge 0$, the action $S_{N,\beta ,\kappa , \zeta }$ for lattice gauge theory with Wilson action coupled to a Higgs field on $B_N$ is, for $\sigma \in \Omega ^1(E_N,G),$ $\phi \in \Omega ^0(B_N,G),$ and a symmetric function $r :C_0(B_N) \rightarrow {\mathbb {R}}_+$, defined by1.1The first term on the right hand side of (1.1) is referred to as the *Wilson action functional* for pure gauge theory (see, e.g., [32]), the second term on the right hand side of (1.1) is referred to as the interaction term, and the third and fourth term on the right hand side of (1.1) together are referred to as a *sombrero potential*. Since $\phi \in \Omega ^0(B_N,G)$ and $\sigma \in \Omega ^1(B_N,G)$, the action $S_{N,\beta ,\kappa ,\zeta }(\sigma , \phi ,r)$ is real for all $\sigma ,$ $\phi ,$ and *r*. Elements $\sigma \in \Omega ^1(B_N,G)$ will be referred to as *gauge field configurations*, and pairs $(\phi ,r),$ with $\psi \in \Omega ^0(B_N,G)$ and $r :C_0(B_N) \rightarrow {\mathbb {R}}_+$ symmetric, will be referred to as *Higgs field configurations*. The quantity $\beta$ is known as the *gauge coupling constant*, $\kappa$ is known as the *hopping parameter*, and $\zeta$ is known as the *quartic Higgs self coupling*.

The Gibbs measure corresponding to the action $S_{N,\beta ,\kappa ,\zeta }$ is given by

$$\begin{aligned} d\mu _{N,\beta , \kappa , \zeta }(\sigma , \phi ,r)  &   = Z^{-1}_{N,\beta ,\kappa , \zeta } e^{-S_{N,\beta ,\kappa ,\zeta }(\sigma , \phi ,r)} \prod _{e \in C_1(B_N)^+} d\mu _G\bigl (\sigma (e)\bigr )\\  &   \prod _{x \in C_0(B_N)^+} d\mu _{G}\bigl (\phi (x)\bigr ) \, d\mu _{{\mathbb {R}}_+}\! \bigl ( r(x)\bigr ), \end{aligned}$$

where $C_1(B_N)^+$ denotes the set of positively oriented edges in $C_1(B_N)$, $d\mu _G$ is the uniform measure on *G*, and $\mu _{{\mathbb {R}}_+}$ is the Lebesgue measure on ${\mathbb {R}}_+$. We refer to this lattice gauge theory as the *abelian lattice Higgs model*.

We will work with the model obtained from this action in the *fixed length limit* $\zeta \rightarrow \infty$, in which the radial component of the Higgs field concentrates at one. In the physics literature, this is sometimes called the *London limit*. We do not discuss the limit of the Gibbs measure corresponding to $S_{N,\beta , \kappa , \zeta }$ as $\zeta \rightarrow \infty$ here, but simply from the outset adopt the action resulting from only considering $r :C_0(B_N) \rightarrow {\mathbb {R}}_+$ with $r(x) = 1$ for all $x \in C_0(B_N).$ In this case, for $\sigma \in \Omega ^1(B_N,G),$ and $\phi \in \Omega ^0(B_N,G)$, we obtain the actionWe then consider a corresponding probability measure $\mu _{N,\beta , \kappa , \infty }$ on $\Omega ^1(B_N,G) \times \Omega ^0(B_N,G)$ given by

$$\begin{aligned} \mu _{N,\beta , \kappa , \infty }(\sigma , \phi ) \, {:}{=}\, Z_{N,\beta ,\kappa , \infty }^{-1} e^{-S_{N,\beta ,\kappa , \infty }(\sigma , \phi )}, \qquad \sigma \in \Omega ^1(B_N,G),\, \phi \in \Omega ^0(B_N,G), \end{aligned}$$

where $Z_{N,\beta ,\kappa , \infty }$ is a normalizing constant. This is the *fixed length lattice Higgs model*. We let ${\mathbb {E}}_{N,\beta ,\kappa ,\infty }$ denote the corresponding expectation. Whenever $f :\Omega ^1(B_M,G) \times \Omega ^0(B_M,G) \rightarrow {\mathbb {R}}$ for some $M \ge 1,$ then, as a consequence of the Ginibre inequalities (see Sect. 2.6), the infinite volume limit

$$\begin{aligned} \bigl \langle f(\sigma ,\phi ) \bigr \rangle _{\beta ,\kappa ,\infty } \, {:}{=}\, \lim _{N \rightarrow \infty } {\mathbb {E}}_{N,\beta ,\kappa ,\infty } \bigl [f(\sigma ,\phi ) \bigr ] \end{aligned}$$

exists, and it is this limit that we will use in our main result.

### Wilson loops and Wilson lines

For $k \in \{ 0,1,\dots , m,$ a *k*-chain is a formal sum of positively oriented k-cells with integer coefficients, see Sect. 2.1.4 below. The support of a 1-chain $\gamma$, written ${{\,\textrm{supp}\,}}\gamma$, is the set of directed edges with non-zero coefficient in $\gamma .$ We say that a 1-chain with finite support is a *generalized loop* if it has coefficients in $\{-1,0,1\}$ and empty boundary, see Definition 2.13. Roughly speaking, this means that a generalized loop is a disjoint union of a finite number of closed loops, where each closed loop is a nearest-neighbor path in the graph ${\mathbb {Z}}^4$ starting and ending at the same vertex. For example, any rectangular loop, as well as any finite disjoint union of such loops, is a generalized loop. We say that a 1-chain with finite support is an *open path* from $x_1\in \Omega _0^+(B_N)$ to $x_2 \in \Omega _2^+(B_N)$ if it has coefficients in $\{-1,0,1\}$ and boundary $\partial \gamma \, {:}{=}\, x_2 - x_1.$ If $\gamma$ is either an open path or a generalized loop, we refer to $\gamma$ as a *path*.

Given a path $\gamma$, the *Wilson line observable* $L_\gamma (\sigma ,\phi )$ is defined by

$$\begin{aligned} L_\gamma (\sigma ,\phi ) \, {:}{=}\, \rho \bigl ( \sigma (\gamma ) - \phi (\partial \gamma ) \bigr ),\qquad \sigma \in \Omega ^1(B_N,G),\, \phi \in \Omega ^0(B_N,G), \end{aligned}$$

where $\sigma (\gamma ) \, {:}{=}\, \sum _{e \in \gamma } \sigma (e),$ and $\phi (\partial \gamma ) = \phi (x_2) - \phi (x_1)$ if $\gamma$ is an open path from $x_1$ to $x_2$, and $\phi (\partial \gamma ) = 0$ if the boundary of $\gamma$ is empty. If $\gamma$ is a generalized loop, then $W_\gamma (\sigma ) \, {:}{=}\, L_\gamma (\sigma ,\phi )$ is referred to as a *Wilson loop observable*.

### Main results

#### Theorem 1.1

Consider the fixed length lattice Higgs model on ${\mathbb {Z}}^4$, with structure group $G = {\mathbb {Z}}_2$, and representation $\rho :G \rightarrow {\mathbb {C}}$ given by $\rho (0) = 1$ and $\rho (1)=-1$.

Let $\beta ,\kappa \ge 0$ be such that $18^2 e^{-4\kappa }(2 + e^{-4\kappa })< 1$ and $6\beta > \kappa .$ Further, let $\gamma$ be a path along the boundary of a rectangle with side lengths $\ell _1,\ell _2 \ge 8,$ and assume that $|{{\,\textrm{supp}\,}}\gamma | \ge 24.$ Finally, let $e \in C_1({\mathbb {Z}}^4)$ be arbitrary.

Then1.2where

$$\begin{aligned} \Theta '_{\beta ,\kappa }(\gamma ) \,&\, {:}{=}\,&\, e^{-2|{{\,\textrm{supp}\,}}\gamma |e^{-24\beta -4\kappa }\bigl (1+(e^{8\kappa }-1) \langle L_{e}(\sigma ,\phi ) \rangle _{\infty ,\kappa ,\infty }\bigr )}, \\ H_\kappa (\gamma ) \,&{:}{=}&\, \bigl \langle L_\gamma (\sigma ,\phi ) \bigr \rangle _{\infty ,\kappa ,\infty }, \end{aligned}$$

and $K_0 = K_0(\kappa ,\beta ,\ell _1,\ell _2,\gamma )$ is a non-negative function with

$$\begin{aligned} K_0 \le 2 \cdot 18^3 +|{{\,\textrm{supp}\,}}\gamma |^{1/2} e^{-4\kappa }\bigl ( 18^2 (2+e^{-4\kappa }) \bigr )^{\min (\ell _1,\ell _2)} + o_\kappa (1). \end{aligned}$$

An exact expression for $K_0$ is given in (10.42).

#### Remark 1.2

We later show, in Corollary 2.18, that if $\gamma$ is an open path, then the function $H_\kappa (\gamma )$ is exactly equal to the spin-spin-correlation of the spins at the endpoints of $\gamma$ in the Ising model with coupling parameter $\kappa .$ By the same argument, the term $\langle L_e(\sigma ,\phi ) \rangle _{\infty ,\kappa ,\infty }$ in the function $\Theta '_{\beta ,\kappa }(\gamma )$ will be equal to the spin-spin-correlation of the spins at the end-points of the (arbitrary) edge *e*.

It is well known (see, e.g., [12]) that when $\kappa$ is larger than the critical parameter for the Ising model, then $H_\kappa (\gamma )$ is uniformly bounded from below for all $\gamma .$ At the same time, by standard arguments, we have $\bigl \langle L_e(\sigma ,\phi ) \bigr \rangle _{\infty ,\kappa ,\infty } = e^{-4 \cdot 8 \kappa } + o_\kappa (1).$

#### Remark 1.3

Using the previous remark, we now interpret our main theorem. To this, end, assume that $\gamma$ is a loop along the boundary of a rectangle *R*. Assume further that the two sides of *R* are of the same order, so that $K_0$ is bounded from above, and that $\beta$ and $|{{\,\textrm{supp}\,}}\gamma |$ are both very large. Then, by Theorem 1.1, the following holds. If $|{{\,\textrm{supp}\,}}\gamma |e^{-24\beta -4\kappa }$ is very large, then $\langle L_\gamma (\sigma ,\phi )\rangle _{\beta ,\kappa ,\infty }$ is very close to zero, and if $|{{\,\textrm{supp}\,}}\gamma |e^{-24\beta -4\kappa }$ is bounded from above, then $\langle L_\gamma (\sigma ,\phi ) \rangle _{\beta ,\kappa ,\infty }$ will be non-trivial.

#### Remark 1.4

The assumption that $18^2 e^{-4\kappa _0}(2 + e^{-4\kappa _0})<1$ guarantees that the clusters formed by the edges in unitary gauge (see Sect. 2.5) are finite almost surely, and this is one of the main properties of the model which we use in the proof of Theorem 1.1. The assumptions that $6\beta \ge \kappa$ and that the path $\gamma$ is along the boundary of a rectangle is used only to simplify $\Theta '_{\beta ,\kappa }(\gamma )$ and $K_0,$ and is not needed for any of the main ideas of the proof. In particular, the strategy used to do this also works for more general classes of loops, as long as their shape is not too rough.

#### Remark 1.5

If $\gamma$ is a generalized loop, then $H_\kappa (\gamma ) = 1$, and hence, in this case, we essentially recover Theorem 1.1 [18].

In Sect. 10, we state a more general version of Theorem 1.1 (Theorem 10.1). While this result is stated for cyclic groups, with minor changes, this paper’s arguments should also work in a more general setting. In particular, the proof strategy should work for all finite abelian groups. Finally, we also mention that alternative versions of our main result, with different error bounds, are given by Proposition 7.1 and Proposition 10.18.

### Applications

We now apply our main result to a few different Wilson lines, and ratios of Wilson lines, which has been considered in the physics literature. In all of these examples, we will work under the assumptions of Theorem 1.1. We note that, when these hold, if $\gamma$ is a loop along a rectangle with side lengths of the same order, then, by Theorem 1.1, we have

$$\begin{aligned} \bigl \langle L_\gamma (\sigma ,\phi ) \bigr \rangle _{\beta ,\kappa ,\infty } = \Theta '_{\beta ,\kappa }(\gamma ) H_\kappa (\gamma ) + o_{\beta }(1) + o_{|{{\,\textrm{supp}\,}}\gamma |}(1). \end{aligned}$$

#### Example 1.6

**Fig. 1:**
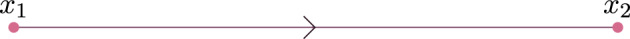
The open path $\gamma$. Note that for any $\ell _1 \ge |x_2-x_1|$ and $\ell _2 \ge 0$ there is a rectangle *R* with side lengths $\ell _1$ and $\ell _2$ so that $\gamma$ is a path along the boundary of *R*, and hence $\gamma$ satisfies the assumptions of Theorem 1.1

In [5], Bricmont and Frölich consider Wilson line observables $L_{\gamma }(\sigma ,\phi )$ for axis parallel paths $\gamma$ which are a shortest path between two points $x_1$ and $x_2$ (see Fig. 1).

The authors argue that the expectation $\langle L_\gamma (\sigma ,\phi ) \rangle _{\beta ,\kappa }$ should exhibit a phase transition, corresponding to binding versus unbinding of dynamical quarks in the field of a static colour source.

In detail, they argue that $\langle L_\gamma (\sigma ,\phi ) \rangle _{\beta ,\kappa ,\infty }$ should have exponential decay with polynomial corrections if $\beta$ is large and $\kappa$ is small, and exponential decay if either $\beta$ is large and $\kappa$ is not too small.

Since, under assumption [A], $H_\kappa (\gamma )$ is uniformly bounded from below for all $\gamma$, and $\Theta '_{\beta ,\kappa }(\gamma )$ has exponential decay in $|{{\,\textrm{supp}\,}}\gamma |,$ we see that $\langle L_\gamma (\sigma ,\phi ) \rangle _{\beta ,\kappa ,\infty }$ indeed has exponential decay in $|{{\,\textrm{supp}\,}}\gamma |$ when $\beta$ is large and $\kappa$ is not too small.

#### Example 1.7

.

**Fig. 2:**
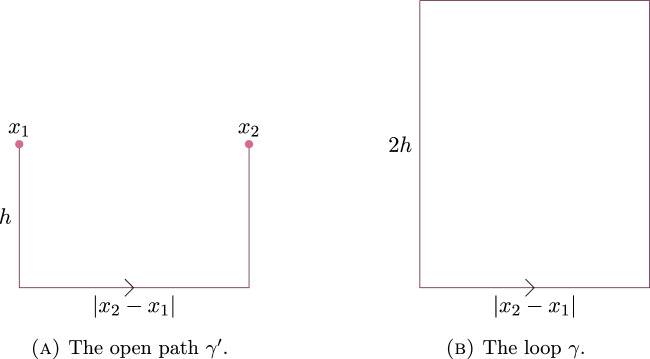
The open path $\gamma '$ and the generalized loop $\gamma$ considered in Example 1.7

Let $\gamma$ and $\gamma '$ be as in Fig. 2. In [15, 29], they consider the ratio

1.3 $$\begin{aligned} \frac{\langle L_{\gamma '}(\sigma ,\phi ) \rangle _{\beta ,\kappa ,\infty }\langle L_{\gamma -\gamma '}(\sigma ,\phi ) \rangle _{\beta ,\kappa ,\infty } }{\langle W_{\gamma }(\sigma )\rangle _{\beta ,\kappa ,\infty }}. \end{aligned}$$

The limit of this ratio, when $|x_2-x_1|$ is proportional to *h* and $h \rightarrow \infty ,$ is often referred to as the *Marcu-Fredenhagen order parameter*.

If this limit is zero, the model in argued to have charged states, and no confinement, whereas if the limit is non-zero, then there should be no charged states and confinement.

We mention that this ratio is also studied in, e.g., [6, 20, 25, 29, 30].

As an immediate consequence of our Theorem 1.1, if $\kappa$ is not too small and $\beta ,$ $\kappa ,$ and $\gamma$ are such that $|{{\,\textrm{supp}\,}}\gamma |e^{-24\beta -4\kappa }$ is bounded away from infinity, then the right hand side of (1.3) is equal to $H_\kappa (\gamma ')^2 + o_{|{{\,\textrm{supp}\,}}\gamma |}(1) + o_{\beta }(1).$

However, since letting $|{{\,\textrm{supp}\,}}\gamma |$ tend to infinity while keeping $\beta$ and $\kappa$ fixed violates that assumption that $|{{\,\textrm{supp}\,}}\gamma |e^{-24\beta -4\kappa }$ is bounded away from infinity, we cannot use this approximate equation to make conclusions about the Marcu-Fredenhagen parameter itself.

#### Example 1.8

**Fig. 3:**
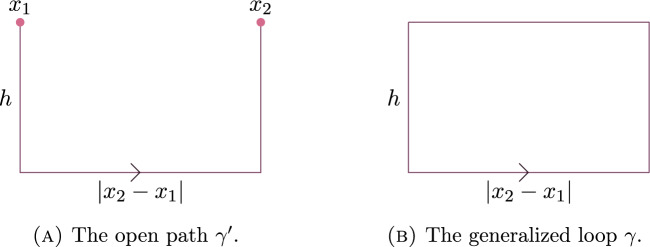
The open path $\gamma '$ and generalized loop $\gamma$ considered in Example 1.8

Let $\gamma$ and $\gamma '$ be as in Fig. 3.

In [23], Gliozzi considers the ratio

1.4 $$\begin{aligned} \frac{ \langle L_{\gamma '}(\sigma ,\phi ) \rangle _{\beta ,\kappa ,\infty } L_{\gamma -\gamma '}(\sigma ,\phi )\rangle _{\beta ,\kappa ,\infty } }{\langle W_{\gamma }(\sigma ) \rangle _{\beta ,\kappa ,\infty }}, \end{aligned}$$

and note that it, asymptotically, seem to only depend on the distance $|x_2-x_1|.$ Indeed, from Theorem 1.1, it follows that if $|{{\,\textrm{supp}\,}}\gamma |e^{-24\beta -4\kappa }$ is bounded away from infinity and $\kappa$ is not too small, then the expression in (1.8) is equal to $H_\kappa (\gamma ')^2 + o_{|{{\,\textrm{supp}\,}}\gamma |}(1) + o_{\beta }(1).$ Using Remark 1.2 to recognise $H_\kappa (\gamma ')$ as the spin-spin-correlation function for the Ising model, evaluated at the end-points of $\gamma '$, this confirms the observation made in [23].

#### Example 1.9

**Fig. 4:**
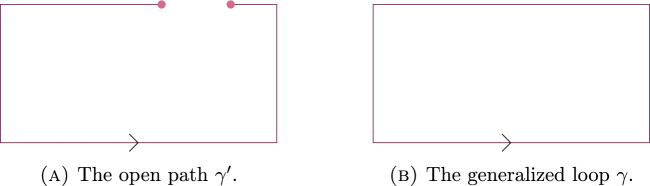
The open path $\gamma '$ and the loop $\gamma$ considered considered in Example 1.9

. Let $\gamma$ and $\gamma '$ be as in Fig. 4, and let *r* be the distance between the endpoints of $\gamma '.$ In [23], when *r* is much smaller that $|{{\,\textrm{supp}\,}}\gamma |,$ the path $\gamma '$ is referred to as an *almost closed Wilson line*, and it was argued that the following functional equation should hold.

1.5 $$\begin{aligned} \bigl \langle L_{\gamma '}(\sigma ,\phi ) \bigr \rangle _{\beta ,\kappa ,\infty } \bigl \langle L_{\gamma -\gamma '}(\sigma ,\phi )\bigr \rangle _{\beta ,\kappa ,\infty } \simeq \bigl \langle W_{\gamma }(\sigma ) \bigr \rangle _{\beta ,\kappa ,\infty } f(r) \end{aligned}$$

for some function *f*(*r*) that should neither depend on $\gamma$ nor on the placement of the open path $\gamma -\gamma '$ on $\gamma .$

Using our main result, it indeed see that if $\kappa$ is not too small, then (assuming that the side lengths of the rectangle are proportional to $|{{\,\textrm{supp}\,}}\gamma |$ is large), we have

$$\begin{aligned} \bigl \langle L_{\gamma '}(\sigma ,\phi ) \bigr \rangle _{\beta ,\kappa ,\infty } \bigl \langle L_{\gamma -\gamma '}(\sigma ,\phi )\bigr \rangle _{\beta ,\kappa ,\infty } = \bigl \langle W_{\gamma }(\sigma ) \bigr \rangle _{\beta ,\kappa ,\infty } H_\kappa (\gamma ')^2 + o_{|{{\,\textrm{supp}\,}}\gamma |}(1) + o_{\beta +6\kappa }(1). \end{aligned}$$

In particular, using Remark 1.2, this shows that the functional equation in (1.5) indeed hold when $|{{\,\textrm{supp}\,}}\gamma |$ and $\beta$ are both large, and with *f*(*r*) given by the spin-spin-correlation function evaluated at the endpoints of $\gamma '.$

### Relation to other work

Many of the ideas used in this paper are refined versions of analogue ideas used in [18], which in turn build upon the works [7, 9, 17]. However, since this paper deals with general paths $\gamma ,$ and not only generalized loops as in [7, 9, 17, 18], the first main idea in these papers, which is to pass from a generalized loop to an oriented surface, does not work. One of the main contributions of this paper thus consists in dealing with this obstacle. Even in the case when the path $\gamma$ in Theorem 1.1 is a generalized loop, our proof is different from that in [18], and we hence provides an alternative proof in this case. In addition, when $\gamma$ is a generalized loop, we express the leading-order term in a more transparent way than in [18].

We mention that although the recent paper [21] also calculate the first order term of Wilson loop observables in an abelian lattice gauge theory, they work with a continuous structure group, and thus their methods are fundamentally different from the ideas used here.

### Structure of paper

In Sect. 2, we give a brief introduction to the cell complex of ${\mathbb {Z}}^m$ and the discrete exterior calculus on this cell complex. We also define vortices and recall some of their properties from [17] to [18]. Moreover, we recall the definition of generalized loops and oriented surfaces from [18], explain unitary gauge and define a corresponding measure, and discuss the existence of the infinite volume limit ${\mathbb {E}}_{\beta ,\kappa ,\infty }[L_\gamma (\sigma ,\phi )].$ In Sect. 3, we introduce additional notation which will be useful throughout the paper. In Sect. 4, we recall the notion of activity of gauge field configurations from [18]. In Sect. 5, we describe a useful edge graph, and introduce two couplings, one between the abelian lattice Higgs model and a ${\mathbb {Z}}_n$-model, and one between two ${\mathbb {Z}}_n$-models. These will be important in the proof of our main result. In Sect. 6, using the edge graph from Sect. 5, we give upper bounds on a number of events related to the couplings introduced in Sect. 5. Next, in Sect. 7, we show how one of the couplings introduced in Sect. 5 can be used to obtain a first version of our main result, which is useful when $|{{\,\textrm{supp}\,}}\gamma | e^{-4(\kappa + 6\beta )}$ is small. This result is not needed for the proof of Theorem 1.1, but illustrates the usefulness of the coupling. In Sect. 8, we introduce a spin decomposition of two coupled configurations. In Sect. 9, we describe how different 1-forms affect the Wilson line observable. Finally, in Sect. 10, we use the setup from the earlier sections to give a proof of our main result.

## Preliminaries

### The cell complex

In this section, we introduce notation for the cell complexes of the lattices ${\mathbb {Z}}^m$ and $B_N \, {:}{=}\, [-N,N]^m \cap {\mathbb {Z}}^m$ for $m,N \ge 1$. This section will closely follow the corresponding section in [17], where we refer the reader for further details.

To simplify notation, we define $e_1 \, {:}{=}\, (1,0,\dots ,0)$, $e_2 \, {:}{=}\, (0,1,0,\dots ,0)$, ..., $e_m \, {:}{=}\, (0,\dots ,0,1)$.

#### Boxes and cubes.

A set *B* of the form $\bigl ( [a_1,b_1] \times \cdots \times [a_m,b_m] \bigr ) \cap {\mathbb {Z}}^m$ where, for each $j \in \{ 1,2, \ldots , m \}$, $\{a_j, b_j\} \subset {\mathbb {Z}}$ satisfies $a_j < b_j$, will be referred to as a *box*. If all the intervals $[a_j,b_j]$, $1 \le j \le m$, have the same length, then the set $\bigl ( [a_1,b_1] \times \cdots \times [a_m,b_m] \bigr ) \cap {\mathbb {Z}}^m$ will be referred to as a *cube*.

#### Non-oriented cells.

When $a \in {\mathbb {Z}}^m$, $k \in \{ 0,1, \dots , m \}$, and $\{ j_1,\dots , j_k \} \subseteq \{ 1,2, \dots , m \}$, we say that the set

$$\begin{aligned} (a; e_{j_1}, \dots , e_{j_k}) \, {:}{=}\, \bigl \{ x \in {\mathbb {R}}^m :\exists b_1, \dots , b_k \in [0,1] \text { such that } x = a + \sum _{i=1}^k b_i e_{j_i} \bigr \} \end{aligned}$$

is a *non-oriented* *k*-*cell*. Note that if $\sigma$ is a permutation, then $(a; e_{j_1}, \dots , e_{j_k})$ and $(a; \sigma (e_{j_1}, \dots , e_{j_k}))$ represent the same non-oriented *k*-cell.

#### Oriented cells.

To each non-oriented *k*-cell $(a; e_{j_1}, \dots , e_{j_k})$ with $a \in {\mathbb {Z}}^m$, $k \ge 1$, and $1\le j_1< \dots < j_k\le m$, we associate two *oriented* *k*-*cells*, denoted $\frac{\partial }{\partial x^{j_1}}\big |_a \wedge \dots \wedge \frac{\partial }{\partial x^{j_k}}\big |_a$ and $-\frac{\partial }{\partial x^{j_1}}\big |_a \wedge \dots \wedge \frac{\partial }{\partial x^{j_k}}\big |_a$, with opposite orientation. When $a \in {\mathbb {Z}}^m$, $1\le j_1< \dots < j_k\le m$, and $\sigma$ is a permutation of $\{ 1,2, \dots , k \}$, we define

$$\begin{aligned} \frac{\partial }{\partial x^{j_{\sigma (1)}}}\bigg |_a \wedge \dots \wedge \frac{\partial }{\partial x^{j_{\sigma (k)}}}\bigg |_a \, {:}{=}\, {{\,\textrm{sgn}\,}}(\sigma ) \, \frac{\partial }{\partial x^{j_1}}\bigg |_a \wedge \dots \wedge \frac{\partial }{\partial x^{j_k}}\bigg |_a \end{aligned}$$

If ${{\,\textrm{sgn}\,}}(\sigma )=1$, then $\frac{\partial }{\partial x^{j_{\sigma (1)}}}\big |_a \wedge \dots \wedge \frac{\partial }{\partial x^{j_{\sigma (k)}}}\big |_a$ is said to be *positively oriented*, and if ${{\,\textrm{sgn}\,}}(\sigma )=-1$, then $\frac{\partial }{\partial x^{j_{\sigma (1)}}}\big |_a \wedge \dots \wedge \frac{\partial }{\partial x^{j_{\sigma (k)}}}\big |_a$ is said to be *negatively oriented*. Analogously, we define

$$\begin{aligned} -\frac{\partial }{\partial x^{j_{\sigma (1)}}}\bigg |_a \wedge \dots \wedge \frac{\partial }{\partial x^{j_{\sigma (k)}}}\bigg |_a \, {:}{=}\, -{{\,\textrm{sgn}\,}}(\sigma ) \, \frac{\partial }{\partial x^{j_1}}\bigg |_a \wedge \dots \wedge \frac{\partial }{\partial x^{j_k}}\bigg |_a, \end{aligned}$$

and say that $-\frac{\partial }{\partial x^{j_{\sigma (1)}}}\bigg |_a \wedge \dots \wedge \frac{\partial }{\partial x^{j_{\sigma (k)}}}\bigg |_a$ is positively oriented if $-{{\,\textrm{sgn}\,}}(\sigma ) = 1$, and negatively oriented if $-{{\,\textrm{sgn}\,}}(\sigma ) = -1.$

Let ${\mathcal {L}} = {\mathbb {Z}}^m$ or ${\mathcal {L}} = B_N \subseteq {\mathbb {Z}}^m$. An oriented cell $\frac{\partial }{\partial x^{j_1}}\big |_a \wedge \dots \wedge \frac{\partial }{\partial x^{j_k}}\big |_a$ is said to be in ${\mathcal {L}}$ if all corners of $(a;e_{j_1},\dots , e_{j_k})$ belong to ${\mathcal {L}}$; otherwise it is said to be *outside* ${\mathcal {L}}$. The set of all oriented *k*-cells in ${\mathcal {L}}$ will be denoted by $C_k({\mathcal {L}}).$ The set of all positively and negatively oriented cells in $C_k({\mathcal {L}})$ will be denoted by $C_k^+({\mathcal {L}})$ and $C_k^-({\mathcal {L}})$, respectively. A set $C \subseteq C_k({\mathcal {L}})$ is said to be *symmetric* if for each $c \in C$ we have $-c \in C$.

A non-oriented 0-cell $$a\in {\mathbb {Z}}^m$$ is simply a point, and to each point we associate two oriented 0-cells $$a^+$$ and $$a^- $$ with opposite orientation. We let $$C_0({\mathcal {L}})$$ denote the set of all oriented 0-cells.

Oriented 1-cells will be referred to as *edges*, and oriented 2-cells will be referred to as *plaquettes*.

#### *k*-chains.

The space of finite formal sums of positively oriented *k*-cells with integer coefficients will be denoted by $C_k({\mathcal {L}},{\mathbb {Z}})$. Elements of $C_k({\mathcal {L}},{\mathbb {Z}})$ will be referred to as *k*-*chains*. If $q \in C_k({\mathcal {L}},{\mathbb {Z}})$ and $c \in C^+_k({\mathcal {L}})$, we let *q*[*c*] denote the coefficient of *c* in *q*. If $c \in C^-_k({\mathcal {L}})$, we let $q[c]\, {:}{=}\, -q[-c].$ For $q,q' \in C_k({\mathcal {L}},{\mathbb {Z}})$, we define

$$\begin{aligned} q+q' \, {:}{=}\, \sum _{c \in C_k^+({\mathcal {L}})} \bigl (q[c] + q'[c] \bigr ) c. \end{aligned}$$

Using this operation, $C_k({\mathcal {L}},{\mathbb {Z}})$ becomes a group.

When $q \in C_k({\mathcal {L}},G)$, we let the *support* of *q* be defined by

$$\begin{aligned} {{\,\textrm{supp}\,}}q \, {:}{=}\, \bigl \{ c \in C_k^+({\mathcal {L}}) :q[c] \ne 0 \bigr \}. \end{aligned}$$

To simplify notation, when $q \in C_k({\mathcal {L}},G)$ and $c\in C_k({\mathcal {L}})$, we write $c \in q$ if either

1. $$c \in C_k^+({\mathcal {L}})$$ and $$q[c]>0$$, or
2. $$c \in C_k^-({\mathcal {L}})$$ and $$q[-c]<0.$$

#### The boundary of a cell

When $k \ge 2$, we define the *boundary* $\partial c \in C_{k-1}({\mathcal {L}}, {\mathbb {Z}})$ of $c = \frac{\partial }{\partial x^{j_1}}\big |_a \wedge \dots \wedge \frac{\partial }{\partial x^{j_k}}\big |_a \in C_k({\mathcal {L}})$ by

2.1 $$\begin{aligned} \partial c \,&\, {:}{=}\, \, \sum _{k' \in \{ 1,\dots , k \}} \biggl ( (-1)^{k'} \frac{\partial }{\partial x^{j_1}}\bigg |_a \wedge \dots \wedge \frac{\partial }{\partial x^{j_{k'-1}}}\bigg |_a \wedge \frac{\partial }{\partial x^{j_{k'+1}}}\bigg |_a \wedge \dots \wedge \frac{\partial }{\partial x^{j_k}}\bigg |_a \nonumber \\&\quad + \, (-1)^{k'+1} \frac{\partial }{\partial x^{j_1}}\bigg |_{a + e_{j_{k'}}} \wedge \dots \wedge \frac{\partial }{\partial x^{j_{k'-1}}}\bigg |_{a + e_{j_{k'}}} \wedge \frac{\partial }{\partial x^{j_{k'+1}}}\bigg |_{a + e_{j_{k'}}} \wedge \dots \wedge \frac{\partial }{\partial x^{j_k}}\bigg |_{a + e_{j_{k'}}} \biggr ). \end{aligned}$$

When $c \, {:}{=}\, \frac{\partial }{\partial x^{j_1}}\big |_a \in C_1({\mathcal {L}})$ we define the boundary $\partial c \in C_0({\mathcal {L}},{\mathbb {Z}})$ by

$$\begin{aligned} \partial c = (-1)^1 a^+ + (-1)^{1+1} (a+e_{j_1})^+ = (a+e_{j_1})^+ - a^+. \end{aligned}$$

We extend the definition of $\partial$ to *k*-chains $q \in C_k({\mathcal {L}},{\mathbb {Z}})$ by linearity. One verifies, as an immediate consequence of this definition, that if $k \in \{ 2,3, \dots , m \}$, then $\partial \partial c = 0$ for any $c \in \Omega _k({\mathcal {L}}).$

#### The coboundary of an oriented cell

If $k \in \{ 0,1, \ldots , n-1 \}$ and $c \in C_k({\mathcal {L}})$ is an oriented *k*-cell, we define the *coboundary* $\hat{\partial }c \in C_{k+1}({\mathcal {L}})$ of *c* as the $(k+1)$-chain

$$\begin{aligned} {\hat{\partial }} c \, {:}{=}\, \sum _{c' \in C_{k+1}({\mathcal {L}})} \bigl (\partial c'[c] \bigr ) c'. \end{aligned}$$

Note in particular that if $c' \in C_{k+1}({\mathcal {L}}),$ then ${\hat{\partial }} c[c'] = \partial c'[c].$ We extend the definition of $\hat{\partial }$ to *k*-chains $q \in C_k({\mathcal {L}},{\mathbb {Z}})$ by linearity.

#### The boundary of a box

An oriented *k*-cell $c = \frac{\partial }{\partial x^{j_1}}\big |_a \wedge \dots \wedge \frac{\partial }{\partial x^{j_k}}\big |_a \in C_k(B_N)$ is said to be a *boundary cell* of a box $B = \bigl ( [a_1,b_1]\times \dots \times [a_m,b_m] \bigr ) \cap {\mathbb {Z}}^m \subseteq B_N$, or equivalently to be in *the boundary* of *B*, if the non-oriented cell $(a;e_{j_1}, \dots , e_{j_k})$ is a subset of the boundary of $[a_1,b_1]\times \dots \times [a_m,b_m].$

When $k \in C_k(B_N)$, we let $\partial C_k(B_N)$ denote the set cells in $C_k(B_N)$ which are boundary cells of $B_N.$

### Discrete exterior calculus

In what follows, we give a brief overview of discrete exterior calculus on the cell complexes of ${\mathbb {Z}}^m$ and $B = [a_1,b_1] \times \dots \times [a_m,b_m] \cap {\mathbb {Z}}^m$ for $m \ge 1$. As with the previous section, this section will closely follow the corresponding section in [17], where we refer the reader for further details and proofs.

All of the results in this subsection are obtained under the assumption that an abelian group *G*, which is not necessarily finite, has been given. In particular, they all hold for both $G={\mathbb {Z}}_n$ and $G={\mathbb {Z}}$.

#### Discrete differential forms

A homomorphism from the group $C_k({\mathcal {L}},{\mathbb {Z}})$ to the group *G* is called a *k*-*form*. The set of all such *k*-forms will be denoted by $\Omega ^k({\mathcal {L}},G)$. This set becomes an abelian group if we add two homomorphisms by adding their values in *G*.

The set $C_k^+({\mathcal {L}})$ of positively oriented *k*-cells is naturally embedded in $C_k({\mathcal {L}},{\mathbb {Z}})$ via the map $c \mapsto 1 \cdot c$, and we will frequently identify $c \in C_k^+({\mathcal {L}})$ with the *k*-chain $1 \cdot c$ using this embedding. Similarly, we will identify a negatively oriented *k*-cell $c \in C_k^-({\mathcal {L}})$ with the *k*-chain $(-1) \cdot (-c)$. In this way, a *k*-form $\omega$ can be viewed as a *G*-valued function on $C_k({\mathcal {L}})$ with the property that $\omega (c) = -\omega (-c)$ for all $c \in C_k({\mathcal {L}})$. Indeed, if $\omega \in \Omega ^k({\mathcal {L}},G)$ and $q = \sum a_i c_i \in C_k({\mathcal {L}},{\mathbb {Z}})$, we have

$$\begin{aligned} \omega (q) = \omega \bigl (\sum a_i c_i \bigr ) = \sum a_i \omega (c_i), \end{aligned}$$

and hence a *k*-form is uniquely determined by its values on positively oriented *k*-cells.

If $\omega$ is a *k*-form, it is useful to represent it by the formal expression

$$\begin{aligned} \sum _{1 \le j_1< \dots < j_k \le m} \omega _{j_1\dots j_k} dx^{j_1} \wedge \cdots \wedge dx^{j_k}. \end{aligned}$$

where $\omega _{j_1\dots j_k}$ is a *G*-valued function on the set of all $a \in {\mathbb {Z}}^m$ such that $\frac{\partial }{\partial x^{j_1}} \big |_a \wedge \dots \wedge \frac{\partial }{\partial x^{j_k}}\big |_a \in C_k({\mathcal {L}})$, defined by

$$\begin{aligned} \omega _{j_1 \dots j_k}(a) = \omega \biggl ( \frac{\partial }{\partial x^{j_1}} \bigg |_a \wedge \dots \wedge \frac{\partial }{\partial x^{j_k}} \bigg |_a \biggr ). \end{aligned}$$

If $1\le j_1< \dots < j_k\le m$ and $\sigma$ is a permutation of $\{ 1,2, \dots , k \}$, we define

$$\begin{aligned} dx^{j_{\sigma (1)}} \wedge \dots \wedge d x^{j_{\sigma (k)}} \, {:}{=}\, {{\,\textrm{sgn}\,}}(\sigma ) \, d x^{j_1} \wedge \dots \wedge d x^{j_k}, \end{aligned}$$

and if $1 \le j_1,\dots , j_k \le n$ are such that $j_i = j_{i'}$ for some $1 \le i < i' \le k$, then we let

$$\begin{aligned} d x^{j_1} \wedge \dots \wedge d x^{j_k} \, {:}{=}\, 0. \end{aligned}$$

Given a *k*-form $\omega$, we let ${{\,\textrm{supp}\,}}\omega$ denote the support of $\omega$, i.e., the set of all oriented *k*-cells *c* such that $\omega (c) \ne 0$. Note that ${{\,\textrm{supp}\,}}\omega$ always contains an even number of elements.

#### The exterior derivative

Given $h :{\mathbb {Z}}^m \rightarrow G$, $a \in {\mathbb {Z}}^m$, and $i \in \{1,2, \ldots , m \}$, we let

$$\begin{aligned} \partial _i h(a) \, {:}{=}\, h(a+e_i) - h(a). \end{aligned}$$

If $k \in \{ 0,1,2, \ldots , m \}$ and $\omega \in \Omega ^k({\mathcal {L}},G)$, we define the $(k+1)$-form $d\omega \in \Omega ^{k+1}({\mathcal {L}},G)$ by

$$\begin{aligned} d\omega = \sum _{1 \le j_1< \dots < j_k \le m} \sum _{i=1}^m \partial _i \omega _{j_1,\dots ,j_k} \, dx^i \wedge (dx^{j_1} \wedge \dots \wedge dx^{j_k}). \end{aligned}$$

The operator *d* is called the *exterior derivative.* Using (2.1), one can show that $\omega \in \Omega ^k({\mathcal {L}},G)$ and $c \in C_k({\mathcal {L}},{\mathbb {Z}})$, we have $d\omega (c) = \omega (\partial c).$ This equality is known as the *discrete Stokes’ theorem.* Recalling that when $k \in \{ 2,3,\dots , m-2\}$ and $c \in C_{k+2}({\mathcal {L}})$, then $\partial \partial c = 0,$ it follows from the discrete Stokes theorem that for any $\omega \in \Omega ^{k}({\mathcal {L}},G),$ we have $dd\omega = 0.$

#### Closed forms and the Poincaré lemma

For $k \in \{ 0,\ldots , m \}$, we say that a *k*-form $\omega \in \Omega ^k({\mathcal {L}},G)$ is *closed* if $d\omega (c) = 0$ for all $c \in C_{k+1}({\mathcal {L}}).$ The set of all closed forms in $\Omega ^k({\mathcal {L}},G)$ will be denoted by $\Omega ^k_0({\mathcal {L}},G).$

##### Lemma 2.1

[The Poincaré lemma, Lemma 2.2 in [9]]. Let $k \in \{ 1, \ldots , m\}$ and let *B* be a box in ${\mathbb {Z}}^m$. Then the exterior derivative *d* is a surjective map from the set $\Omega ^{k-1}(B \cap {\mathbb {Z}}^m, G)$ to $\Omega ^k_0(B \cap {\mathbb {Z}}^m, G)$. Moreover, if *G* is finite, then this map is an $\bigl | \Omega ^{k-1}_0(B \cap {\mathbb {Z}}^m, G)\bigr |$-to-1 correspondence.

Lastly, if $k \in \{ 1,2, \ldots , m-1 \}$ and $\omega \in \Omega ^k_0(B \cap {\mathbb {Z}}^m, G)$ vanishes on the boundary of *B*, then there is a $(k-1)$-form $\omega ' \in \Omega ^{k-1}(B \cap {\mathbb {Z}}^m, G)$ that also vanishes on the boundary of *B* and satisfies $d\omega ' = \omega$.

#### Non-trivial forms.

We say that a *k*-form $\omega \in \Omega ^k({\mathcal {L}},G)$ is *non-trivial* if there is at least one *k*-cell $c \in C_k({\mathcal {L}})$ such that $\omega (c) \ne 0$.

#### Restrictions of forms.

If $\omega \in \Omega ^k({\mathcal {L}},G)$, $C \subseteq C_k({\mathcal {L}})$ is symmetric, and $c \in C$, we define

$$\begin{aligned} \omega |_C(c) \, {:}{=}\, {\left\{ \begin{array}{ll} \omega (c) & \text {if } c \in C, \\ 0 & \text {else.} \end{array}\right. } \end{aligned}$$

#### A partial ordering of $\Omega ^k({\mathcal {L}},G)$.

We now recall the partial ordering on differential forms, which was introduced in [18].

##### Definition 2.2

. When $k \in \{ 0,1,\dots , m \}$ and $\omega ,\omega ' \in \Omega ^k({\mathcal {L}},G)$, we write $\omega ' \le \omega$ if

(i) $$\omega ' = \omega |_{{{\,\textrm{supp}\,}}\omega '}$$, and
(ii) $$d\omega ' = (d\omega )|_{{{\,\textrm{supp}\,}}d\omega '}$$.

If $\omega ' \ne \omega$ and $\omega ' \le \omega$, we write $\omega '< \omega$.

The following lemma from [18] collects some basic facts about the relation $\le$ on $\Omega ^k({\mathcal {L}},G)$, and shows that $\le$ is a partial order on $\Omega ^k({\mathcal {L}},G)$.

##### Lemma 2.3

[Lemma 2.7 in [18]]. Let $k \in \{ 0,1,\dots , m \}$ and $\omega , \omega ', \omega '' \in \Omega ^k({\mathcal {L}},G)$. The relation $\le$ on $\Omega ^k({\mathcal {L}},G)$ has the following properties.

(i) Reflexivity: $$\omega \le \omega $$.
(i) Antisymmetry: If $\omega ' \le \omega$ and $\omega \le \omega '$, then $\omega = \omega '$.
(iii) Transitivity: If $\omega '' \le \omega '$ and $\omega ' \le \omega$, then $\omega '' \le \omega$.
(iv) If $\omega ' \le \omega$, then $\omega -\omega ' = \omega |_{C_1(B_N) \smallsetminus ({{\,\textrm{supp}\,}}\omega ')} \le \omega$.
(v) If $\omega ' \le \omega$, then ${{\,\textrm{supp}\,}}d\omega '$ and ${{\,\textrm{supp}\,}}d(\omega -\omega ')$ are disjoint.

The next lemma guarantees the existence of minimal elements satisfying certain constraints.

##### Lemma 2.4

[Lemma 2.8 in [18]]. Let $k \in \{ 0,1,\dots , m \}$, let $\Omega \subseteq \Omega ^k({\mathcal {L}},G)$, and let $\omega \in \Omega$. Then there is $\omega ' \le \omega$ such that

(i) $$ \omega ' \in \Omega $$, and
(ii) There is no $\omega '' < \omega '$ such that $\omega '' \in \Omega$.

#### Irreducible forms

The partial ordering given in Definition 2.2 allows us to introduce a notion of irreducibility.

##### Definition 2.5

[Definition 2.9 in [18]]. When $k \in \{ 0,1,\dots , m-1 \}$, a *k*-form $\omega \in \Omega ^k({\mathcal {L}},G)$ is said to be *irreducible* if there is no non-trivial *k*-form $\omega ' \in \Omega ^k({\mathcal {L}},G)$ such that $\omega ' < \omega$.

Equivalently, $\omega \in \Omega ^k({\mathcal {L}},G)$ is irreducible if there is no non-empty set $S \subsetneq {{\,\textrm{supp}\,}}\omega$ such that ${{\,\textrm{supp}\,}}d(\omega |_S)$ and ${{\,\textrm{supp}\,}}d (\omega |_{S^c})$ are disjoint. Note that if $\omega \in \Omega ^k({\mathcal {L}},G)$ satisfies $d\omega = 0$, then $\omega$ is irreducible if and only if there is no non-empty set $S \subsetneq {{\,\textrm{supp}\,}}\omega$ such that $d(\omega |_S) = d(\omega |_{S^c}) = 0$.

##### Lemma 2.6

[Lemma 2.10 in [18]]. Let $k \in \{ 0,1,\dots , m-1 \}$, and let $\omega \in \Omega ^k({\mathcal {L}},G)$ be non-trivial and have finite support.

Then there is an integer $j \ge 1$ and *k*-forms $\omega _1, \ldots , \omega _j \in \Omega ^k({\mathcal {L}},G)$ such that

(i) For each $i \in \{ 1,2, \ldots , j \}$, $\omega _i$ is non-trivial and irreducible,
(ii) For each $i \in \{ 1,2, \ldots , j \}$, $\omega _i \le \omega$,
(iii) $\omega _1, \dots , \omega _j$ have disjoint supports,
(iv) $$\omega = \omega _1 + \dots + \omega _j$$, and
(v) $d\omega _1, \dots , d\omega _j$ have disjoint supports.

A set $\Omega \, {:}{=}\, \{ \omega _1, \dots , \omega _j \} \subseteq \Omega ^k({\mathcal {L}},G)$ such that $\omega _1, \dots , \omega _j$ satisfies ((i)) –((v)) of Lemma 2.6 will be referred to as a *decomposition* of $\omega \in \Omega ^k({\mathcal {L}},G).$

We note that as an immediate consequence of the previous lemma, if $\omega \in \Omega ^2_0({\mathcal {L}},G)$ has finite support, then there is a set $\Omega \subseteq \Omega ^2_0({\mathcal {L}},G)$ which is a decomposition of $\omega$ (see also Lemma 2.12 in [18]).

#### Minimal forms.

In this section, we recall three lemmas from [18] which gives lower bounds on the size of the support of differential forms. Throughout this section, we assume that $m = 4$. In other words, we assume that we are working on the ${\mathbb {Z}}^4$-lattice.

##### Lemma 2.7

[Lemma 2.16 in [18]]. Let $\sigma \in \Omega ^1({\mathcal {L}},G)$. Then

$$\begin{aligned} |{{\,\textrm{supp}\,}}\sigma | \ge |{{\,\textrm{supp}\,}}d \sigma |/6. \end{aligned}$$

##### Lemma 2.8

Let $\omega \in \Omega ^2_0({\mathcal {L}},G)$ be non-trivial and have finite support, and assume that there is a plaquette $p \in {{\,\textrm{supp}\,}}\omega$ such that ${{\,\textrm{supp}\,}}\partial p$ contains no boundary edges of $B_N.$

Then $| ({{\,\textrm{supp}\,}}\omega )^+ | \ge 6,$ and if $| ({{\,\textrm{supp}\,}}\omega )^+| = 6$, then there is an edge $e_0 \in C_1(B_N)$ such that ${{\,\textrm{supp}\,}}\nu = {{\,\textrm{supp}\,}}{\hat{\partial }} e_0 \cup {{\,\textrm{supp}\,}}\hat{\partial }(-e_0)$.

For a proof of Lemma 2.8, see, e.g., Lemma 3.4.6 in [7].

##### Lemma 2.9

[Lemma 2.19 in [18]]. Let $\sigma \in \Omega ^1_0(B_N,G)$ be non-trivial, and assume that there is an edge $e\in {{\,\textrm{supp}\,}}\sigma$ such that the support of ${\hat{\partial }} e$ contains no boundary cells of $B_N$. Then $|({{\,\textrm{supp}\,}}\sigma )^+ | \ge 8$.

### Vortices

In this section, we use the notion of irreducibility introduced in Sect. 2.2.7 to define what we refer to as vortices. We mention that the definition of a vortex given in Definition 2.10 below is identical to the definitions used in [17, 18], but is different from the corresponding definitions in [7, 9].

#### Definition 2.10

. Let $\sigma \in \Omega ^1(B_N,G)$. A non-trivial and irreducible 2-form $\nu \in \Omega ^2_0(B_N,G)$ is said to be a *vortex* in $\sigma$ if $\nu \le d\sigma$, i.e., if $d\sigma (p) = \nu (p)$ for all $p \in {{\,\textrm{supp}\,}}\nu$.

We say that $\sigma \in \Omega ^1(B_N,G)$ has a vortex at $V \subseteq C_2(B_N)$ if $(d\sigma )|_V$ is a vortex in $\sigma$.

#### Lemma 2.11

[Lemma 3.6 in [18]]. Let $\sigma ',\sigma \in \Omega ^1(B_N,G)$ be such that $\sigma ' \le \sigma$, and let $\nu \in \Omega ^2_0(B_N,G)$ be a vortex in $\sigma '$. Then $\nu$ is a vortex in $\sigma$.

With Lemma 2.8 in mind, we say that a vortex $\nu$ such that no plaquette in ${{\,\textrm{supp}\,}}\nu$ is a boundary plaquette of ${\mathcal {L}}$ is a *minimal vortex* if $|{{\,\textrm{supp}\,}}\nu | = 12$.

#### Lemma 2.12

[Lemma 3.2 in [18]]. Let $\sigma \in \Omega ^1(B_N,G)$, and let $\nu \in \Omega ^2_0(B_N,G)$ be a minimal vortex in $\sigma$. Then there is an edge $\partial x_j \in C_1(B_N)$ and a group element $g \in G\smallsetminus \{ 0 \}$ such that

2.2 $$\begin{aligned} \nu = d\bigl (g \, d x_j \bigr ). \end{aligned}$$

In particular, $d\sigma (p) = \nu (p) = g$ whenever $p \in \hat{\partial }e_0$.

If $\sigma \in \Omega ^1(B_N,G)$ and $\nu \in \Omega ^2_0(B_N,G)$ is a minimal vortex in $\sigma$ which can be written as in (2.2) for some $e_0 \in C_1(B_N)$ and $g \in G \backslash \{ 0 \}$, then we say that $\nu$ is a *minimal vortex centered at* $e_0$.

### Generalized loops and oriented surfaces

In this section, we recall the definitions of generalized loops and oriented surfaces from [17], and outline their connection.

#### Definition 2.13

. A 1-chain $\gamma \in C_1({\mathcal {L}},{\mathbb {Z}})$ with finite support is a *generalized loop* if

1. for all $e \in \Omega ^1({\mathcal {L}})$, we have $\gamma [e] \in \{ -1,0,1 \}$, and
2. $$\partial \gamma = 0.$$

#### Definition 2.14

. Let $\gamma \in C_1({\mathcal {L}},{\mathbb {Z}})$ be a generalized loop. A 2-chain $q \in C_2({\mathcal {L}},{\mathbb {Z}})$ is an *oriented surface* with *boundary* $\gamma$ if $\partial q = \gamma .$

We recall that by Stokes’ theorem (see Sect. 2.2.2), for any $q \in C_2({\mathcal {L}},G)$ and any $\sigma \in \Omega ^1({\mathcal {L}},G)$, we have

$$\begin{aligned} \sigma (\partial q) = d\sigma (q). \end{aligned}$$

The following lemma gives a connection between generalized loops and oriented surfaces.

#### Lemma 2.15

[Lemma 2.8 in [17]]. Let $\gamma \in C_1({\mathcal {L}}, {\mathbb {Z}})$ be a generalized loop, and let $B \subseteq {\mathcal {L}}$ be a box containing the support of $\gamma$. Then there is an oriented surface $q \in C_2({\mathcal {L}}, {\mathbb {Z}})$ with support contained in *B* such that $\gamma$ is the boundary of *q*.

### Unitary gauge

In this section, we introduce gauge transforms, and the describe how these can be used to rewrite the Wilson line expectation as an expectation with respect to a slightly simpler probability measure.

Before we can state the main results of this section, we need to briefly discuss gauge transformations. To this end, for $\eta \in \Omega _0(B_N,G)$, consider the bijection $\tau \, {:}{=}\, \tau _\eta \, {:}{=}\, \tau _\eta ^{(1)} \times \tau _\eta ^{(2)} :\Omega ^1(B_N,G) \times \Omega ^0(B_N,G) \rightarrow \Omega _1(B_N,G) \times \Omega ^0(B_N,G)$, defined by

2.3 $$\begin{aligned} {\left\{ \begin{array}{ll} \sigma (e) \mapsto -\eta (x) +\sigma (e) + \eta (y), &  e=(x,y)\in C_1(B_N), \\ \phi (x) \mapsto \phi (x) + \eta (x), &  x \in C_0(B_N). \end{array}\right. } \end{aligned}$$

Any mapping $\tau$ of this form is called a *gauge transformation*. Any mapping $\tau$ of this form is called a *gauge transformation*, and functions $f: \Omega ^1(B_N,G) \times \Omega ^0(B_N,G) \rightarrow {\mathbb {C}}$ which are invariant under such mappings in the sense that $f= f \circ \tau$ are said to be *gauge invariant*.

For $\beta , \kappa \ge 0$ and $\sigma \in \Omega ^1(B_N,G)$, define2.4where $Z_{N,\beta ,\kappa }^{-1}$ is a normalizing constant which ensures that $\mu _{N,\beta ,\kappa }$ is a probability measure. We let ${\mathbb {E}}_{N,\beta ,\kappa }$ denote the corresponding expectation.

The main reason that gauge transformations are useful to us is the following result.

#### Proposition 2.16

[Proposition 2.21 in [18]]. Let $\beta ,\kappa \ge 0$, and let and assume that the function $f :\Omega ^1(B_N,G) \times \Omega ^0(B_N,G) \rightarrow {\mathbb {C}}$ is gauge invariant. Then

$$\begin{aligned} {\mathbb {E}}_{N,\beta ,\kappa ,\infty }\bigl [f(\sigma ,\phi )\bigr ] = {\mathbb {E}}_{N,\beta ,\kappa }\bigl [f(\sigma ,1)\bigr ]. \end{aligned}$$

The main idea of the proof of Proposition 2.16 is to perform a change of variables, where we for each pair $(\sigma ,\phi )$ apply the gauge transformation $\tau _{-\phi },$ thus mapping $\phi$ to 0. After having applied this gauge transformation, we are said to be working in *unitary gauge*.

Noting that for any path $\gamma$, the function $(\sigma ,\phi ) \mapsto L_\gamma (\sigma ,\phi )$ is gauge invariant, we obtain the following result as an immediate corollary of Proposition 2.16.

#### Corollary 2.17

Let $\beta \in [0,\infty ],$ $\kappa \ge 0$, and let $\gamma$ be a path in $C_1(B_N)$. Then

Results analogous to Proposition 2.16 are considered well-known in the physics literature.

By combining the previous result with Lemma 2.1, we obtain the following result, which will help us interpret our main result.

#### Corollary 2.18

Let $\kappa \ge 0$, and let $\gamma$ be an open path from $x_1 \in C_0(B_N)$ to $x_2 \in C_0(B_N).$ Then

$$\begin{aligned} H_\kappa (\gamma ) = \lim _{N \rightarrow \infty } Z_{N,\kappa }^{-1} \sum _{\eta \in \Omega ^0(B_N,G)} \rho \bigl (\eta (-x_1)\bigr )\rho \bigl (\eta (x_2)\bigr )e^{-\kappa \sum _{e \in C_1(B_N)} \rho (\eta (\partial e))}, \end{aligned}$$

where

$$\begin{aligned} Z_{N,\kappa } \, {:}{=}\, \sum _{\eta \in \Omega ^0(B_N,G)} e^{\kappa \sum _{e \in C_1(B_N)}\rho (\eta (\partial e))}. \end{aligned}$$

If particular, if $G = {\mathbb {Z}}_2,$ then $H_\kappa (\gamma )$ is the spin-spin-correlation between for the spins at the endpoints of $\gamma$ for the Ising model on $B_N$ with coupling constant $\kappa .$

#### Proof

By Corollary 2.17, we have

$$\begin{aligned} \begin{aligned} H_\kappa (\gamma )&= \bigl \langle L_\gamma (\sigma ,\phi ) \bigr \rangle _{\infty ,\kappa ,\infty } = \lim _{N \rightarrow \infty } \bigl \langle L_\gamma (\sigma ,\phi )\bigr \rangle _{N,\infty ,\kappa ,\infty } \\&= \lim _{N \rightarrow \infty }{\mathbb {E}}_{N,\infty ,\kappa ,\infty } \bigl [ L_\gamma (\sigma ,\phi ) \bigr ] = \lim _{N \rightarrow \infty }{\mathbb {E}}_{N,\infty ,\kappa } \bigl [ L_\gamma (\sigma ,1) \bigr ] \\&= \lim _{N \rightarrow \infty } Z_{N,\infty ,\kappa ,\infty }^{-1} \sum _{\sigma \in \Omega ^1_0(B_N,G)} \rho (\sigma ( \gamma ) ) e^{\kappa \sum _{e \in C_1(B_N)} \rho (\sigma ( e))}. \end{aligned} \end{aligned}$$

Since $\beta = \infty ,$ we only need to sum over $\sigma \in \Omega ^1_0(B_N,G).$ Now recall that by Lemma 2.1, for each $\sigma \in \Omega ^1_0(B_N,G)$ there is $\eta \in \Omega ^0(B_N,G)$ such that $d \eta = \sigma .$ Moreover, the mapping $\eta \mapsto d\eta$ is a $|\Omega _0^0(B_N,G)|$-to-1 correspondence. From this the desired conclusion immediately follows. $\square$

With the current section in mind, we will work with $\sigma \sim \mu _{N,\beta , \kappa }$ rather than $(\sigma ,\phi ) \sim \mu _{N,\beta ,\kappa ,\infty }$ throughout the rest of this paper, together with the observable

$$\begin{aligned} L_\gamma (\sigma ) \, {:}{=}\, L_\gamma (\sigma ,1) = \prod _{e \in \gamma } \rho \bigl (\sigma (e)\bigr ) = \rho (\sigma (\gamma )). \end{aligned}$$

### Existence of the infinite volume limit

In this section, we recall a result which shows existence and translation invariance of the infinite volume limit $\langle L_\gamma (\sigma ,\phi ) \rangle _{\beta ,\kappa }$ defined in the introduction. This result is well-known, and is often mentioned in the literature as a direct consequence of the Ginibre inequalities. A full proof of this result in the special case $\kappa = 0$ was included in [17], and the general case can be proven completely analogously, hence we omit the proof here.

#### Proposition 2.19

Let $G = {\mathbb {Z}}_n$, $M \ge 1$, and let $f :\Omega ^1(B_M,G) \rightarrow {\mathbb {R}}$.

For $M' \ge M$, we abuse notation and let *f* denote the natural extension of *f* to $C_1(B_{M'})$, i.e., the unique function such that $f(\sigma ) = f(\sigma |_{C_1(B_M)})$ for all $\sigma \in \Omega ^1(B_{M'},G)$.

Further, let $\beta \in [0, \infty ]$ and $\kappa \ge 0$. Then the following hold.

(i) The limit $\lim _{N \rightarrow \infty } {\mathbb {E}}_{N,\beta ,\kappa } \bigl [ f(\sigma ) \bigr ]$ exists.
(ii) For any translation $\tau$ of ${\mathbb {Z}}^m$, we have $\lim _{N \rightarrow \infty } {\mathbb {E}}_{N,\beta ,\kappa } \bigl [f \circ \tau (\sigma )\bigr ] = \lim _{N \rightarrow \infty } {\mathbb {E}}_{N,\beta ,\kappa }\bigl [ f(\sigma ) \bigr ]$.

## Additional Notation and Standing Assumptions

Throughout the rest of this paper, we will assume that $N \ge 1$ is given, and that $G = {\mathbb {Z}}_n$ for some $n \ge 2.$

To simplify the notation, we now introduce some additional notation.

For $r \ge 0$ and $g \in G$, we define

3.1 $$\begin{aligned} \varphi _r(g) \, {:}{=}\, e^{r \Re (\rho (g)-\rho (0))}. \end{aligned}$$

We extend this notation to $r = \infty$ by letting

$$\begin{aligned} \varphi _\infty (g) \, {:}{=}\, {\left\{ \begin{array}{ll} 1 & \text {if } g = 0, \\ 0 &  \text {if }g \in G \smallsetminus \{0\}. \end{array}\right. } \end{aligned}$$

Next, for ${\hat{g}} \in G$ and $\beta ,\kappa \ge 0$, we define

3.2 $$\begin{aligned} \theta _{\beta ,\kappa }({\hat{g}}) \, {:}{=}\, \frac{\sum _{g \in G} \rho (g) \varphi _\beta (g)^{12} \varphi _\kappa (g+{\hat{g}})^2}{\sum _{g \in G} \varphi _\beta (g)^{12} \varphi _\kappa (g+{\hat{g}})^2}. \end{aligned}$$

When $\gamma$ is a path, or when $E \subseteq C_1(B_N)$ is a finite set, we define

3.3 $$\begin{aligned} \Theta _{N,\beta ,\kappa }(\gamma ) \, {:}{=}\, {\mathbb {E}}_{N,\infty ,\kappa } \Bigl [ \prod _{e \in \gamma } \theta _{\beta ,\kappa }\bigl (\sigma (e)\bigr )\Bigr ] \quad \text {and} \quad \Theta _{N,\beta ,\kappa }(E) \, {:}{=}\, {\mathbb {E}}_{N,\infty ,\kappa } \Bigl [ \prod _{e \in E} \theta _{\beta ,\kappa }\bigl (\sigma (e)\bigr )\Bigr ].\nonumber \\ \end{aligned}$$

We next define a number of functions which will be used as error bounds. To this end, for $r \ge 0$, let

3.4 $$\begin{aligned} \alpha _0(r) \, {:}{=}\, \sum _{g \in G\smallsetminus \{ 0 \}} \varphi _r(g)^2 \quad \text {and} \quad \alpha _1(r) \, {:}{=}\, \max _{g \in G\smallsetminus \{ 0 \}} \varphi _r(g)^2. \end{aligned}$$

Next, for $\beta ,\kappa \ge 0$, define

3.5 $$\begin{aligned} \alpha _2(\beta ,\kappa )  &   \, {:}{=}\, \alpha _0(\beta ) \alpha _0(\kappa )^{1/6}, \quad \alpha _3(\beta ,\kappa ) \, {:}{=}\, \bigl | 1- \theta _{\beta ,\kappa }(0)\bigr |, \quad \alpha _4(\beta ,\kappa )\nonumber \\  &   \, {:}{=}\, \max _{g \in G} \bigl | \theta _{\beta ,\kappa }(g)- \theta _{\beta ,\kappa }(0)\bigr |, \end{aligned}$$

3.6 $$\begin{aligned}  &   \alpha _5(\beta ,\kappa ) \, {:}{=}\, \min _{g_1,g_2, \ldots , g_6 \in G}\bigg ( 1 - \biggl | \frac{\sum _{g \in G} \rho (g) \bigl (\prod _{k =1}^6 \varphi _\beta (g+g_k)^2\bigr ) \varphi _\kappa (g)^2}{\sum _{g \in G} \bigl (\prod _{k =1}^6\varphi _\beta (g+g_k)^2\bigr ) \varphi _\kappa (g)^2} \biggr |\bigg )\nonumber \\ \end{aligned}$$

and

3.7 $$\begin{aligned} \alpha _6(\beta ,\kappa ) \, {:}{=}\, \max _{g \in G}\bigl | 1 - \theta _{\beta ,\kappa }( g) \bigr |. \end{aligned}$$

When $\gamma$ is a path, an edge $e \in {{\,\textrm{supp}\,}}\gamma$ is said to be a corner edge in $\gamma$ if there is another edge $e' \in \gamma$ and a plaquette $p \in {\hat{\partial }} e$ such that $p \in \pm {\hat{\partial }} e'$. We define the 1-form $\gamma _c \in C_1({\mathcal {L}},{\mathbb {Z}})$ for $c' \in C_1(B_N)$ by

3.8 $$\begin{aligned} \gamma _c[c'] \, {:}{=}\, {\left\{ \begin{array}{ll} \gamma [c'] & \text {if } c' \text { is a corner edge of } \gamma , \\ 0 & \text {else.} \end{array}\right. } \end{aligned}$$

In the rest of this paper, we will often work under the following assumption.

[A] $$18^2 \alpha _0(\kappa )(2 + \alpha _0(\kappa ))<1$$.

In essence, the purpose of this assumption is to guarantee that we are in the sub-critical regime of the model, where certain edge clusters are finite almost surely.

## Activity of Gauge Field Configurations

In this section, we recall the useful notion of the activity of a gauge field configurations from [18]. To this end, recall the definition of $\varphi _r$ from the previous section. Since $\rho$ is a unitary representation of *G*, for any $g \in G$ we have $\rho (g) = \overline{\rho (-g)}$, and hence $\Re \rho (g) = \Re \rho (-g)$. In particular, this implies that for any $g \in G$ and any $r \ge 0$, we have

4.1 $$\begin{aligned} \varphi _r(g) = e^{ r (\Re \rho (g)-\rho (0)) } = e^{r \beta (\Re \rho (-g)-\rho (0)) } = \varphi _r(-g). \end{aligned}$$

Clearly, we also have $\varphi _\infty (g) = \varphi _\infty (-g)$ for all $g \in G$. Moreover, if $a \ge 0$ and $r \ge 0$, then

$$\begin{aligned} \varphi _r(g)^a = \varphi _{ar}(g). \end{aligned}$$

Abusing notation, for $\sigma \in \Omega ^1(B_N,G)$ and $r \in [0,\infty ]$, we define

$$\begin{aligned} \varphi _r(\sigma ) \, {:}{=}\, \prod _{e \in C_1(B_N)} \varphi _r\bigl (\sigma (e)\bigr ), \end{aligned}$$

and for $\omega \in \Omega ^2_0(B_N,G)$, we define

$$\begin{aligned} \varphi _r(\omega ) \, {:}{=}\, \prod _{p \in C_2(B_N)}\varphi _r\bigl (\omega (p)\bigr ). \end{aligned}$$

For $\beta \in [0,\infty ]$ and $\kappa \ge 0$, we define the *activity* of $\sigma \in \Omega ^1(B_N,G)$ by

$$\begin{aligned} \varphi _{\beta ,\kappa }(\sigma ) \, {:}{=}\, \varphi _\kappa (\sigma ) \varphi _\beta (d\sigma ). \end{aligned}$$

Note that with this notation, for $\sigma \in \Omega ^1(B_N,G)$, $\beta \in [0,\infty ]$, and $\kappa \ge 0$, we have

4.2 $$\begin{aligned} \mu _{N,\beta ,\kappa }(\sigma ) = \frac{\varphi _{\beta ,\kappa } (\sigma )}{\sum _{\sigma ' \in \Omega ^1(B_N,G)} \varphi _{\beta ,\kappa }(\sigma ')}. \end{aligned}$$

Before ending this section, we recall two results from [18] about the activity of gauge field configurations, which will be useful to us.

### Lemma 4.1

[Lemma 4.1 in [18]]. Let $\sigma ,\sigma ' \in \Omega ^1(B_N,G)$ be such that $\sigma ' \le \sigma$, let $\beta \in [0,\infty ]$, and let $\kappa \ge 0$. Then

4.3 $$\begin{aligned} \varphi _{\beta ,\kappa }(\sigma ) = \varphi _{\beta ,\kappa }(\sigma ')\varphi _{\beta ,\kappa }(\sigma -\sigma '). \end{aligned}$$

### Proposition 4.2

[Proposition 5.1 in [18]]. Let $\sigma ' \in \Omega ^1(B_N,G)$, let $\beta \in [0,\infty ]$, and let $\kappa \ge 0$. Then

## Two Couplings

The main purpose of this section is to introduce two couplings which will be useful to us throughout this paper. Both of these couplings use ideas from disagreement percolation, and will be constructed so that the two coupled configurations agree as often as possible, given certain constraints. Before we introduce the two couplings, we will recall the definition of a certain edge graph from [16], and state and prove some of its properties, and introduce a set $E_{E_0,{\hat{\sigma }}, {\hat{\sigma }}'}$ which will be used for the definitions of the two couplings.

### A useful edge graph

#### Definition 5.1

Given $\sigma ,\sigma ' \in \Omega ^1(B_N,G)$, let ${\mathcal {G}}(\sigma , \sigma ')$ be the graph with vertex set $C_1(B_N)$, and with an edge between two distinct vertices $e,e' \in C_1(B_N)$ if either

(i) $$e' = -e$$, or
(ii) $e,e' \in {{\,\textrm{supp}\,}}\sigma \cup {{\,\textrm{supp}\,}}\sigma '$, and either ${{\,\textrm{supp}\,}}{\hat{\partial }} e \cap {{\,\textrm{supp}\,}}{\hat{\partial }} e' \ne \emptyset$ or ${{\,\textrm{supp}\,}}{\hat{\partial }} e \cap {{\,\textrm{supp}\,}}\hat{\partial }(-e') \ne \emptyset$.

Given $\sigma \in \Omega ^1(B_N),$ we let ${\mathcal {G}}(\sigma ) \, {:}{=}\, {\mathcal {G}}(\sigma ,0).$

Given $\sigma ,\sigma ' \in \Omega ^1(B_N),$ ${\mathcal {G}} \, {:}{=}\, {\mathcal {G}}(\sigma , \sigma '),$ and $e \in C_1(B_N),$ we let ${\mathcal {C}}_{{\mathcal {G}}}(e)$ be set of all edges $e'\in C_1(B_N)$ which belong to the same connected component as *e* in ${\mathcal {G}}$. For $E \subseteq C_1(B_N)$, we let ${\mathcal {C}}_{{\mathcal {G}}}(E) \, {:}{=}\, \bigcup _{e \in E} {\mathcal {C}}_{{\mathcal {G}}}(e).$

We now state and prove a number of lemmas, which describe different properties of the sets ${\mathcal {C}}_{{\mathcal {G}}({\hat{\sigma }},\hat{\sigma }')}(E).$

#### Lemma 5.2

[Lemma 7.2 in [18]]. Let $\sigma ,\sigma ' \in \Omega ^1(B_N,G)$, $E \subseteq C_1(B_N)$, and $E' \, {:}{=}\, {\mathcal {C}}_{{\mathcal {G}}(\sigma , \sigma ')}(E)$. Then

(i) $$\sigma |_{E'} \le \sigma $$,
(ii) $$\sigma |_{C_1(B_N)\smallsetminus E'} \le \sigma $$,
(iii) $$\sigma ' |_{E'} \le \sigma '$$, and
(iv) $$\sigma ' |_{C_1(B_N) \smallsetminus E'} \le \sigma '$$.

#### Lemma 5.3

Let $\sigma \in \Omega ^1(B_N,G)$ be nontrivial and irreducible. Then the support of $\sigma$ is a connected set in ${\mathcal {G}}(\sigma ).$

#### Proof

Let $e \in {{\,\textrm{supp}\,}}\sigma$, and define $\sigma ' \, {:}{=}\, \sigma |_{{\mathcal {C}}_{{\mathcal {G}}(\sigma )}(e)}.$ Then, by definition, $\sigma '$ is non-trivial, and by Lemma 5.2, we have $\sigma ' \le \sigma .$ Since $\sigma$ is irreducible, it follows that $\sigma = \sigma ',$ and hence the desired conclusion follows. $\square$

#### Lemma 5.4

Let $\sigma ,\sigma ' \in \Omega ^1(B_N,G)$. Assume that $\sigma '' \le \sigma$ is nontrivial and irreducible, and let $e \in {{\,\textrm{supp}\,}}\sigma ''.$ Then

5.1 $$\begin{aligned} \sigma '' \le \sigma |_{{\mathcal {C}}_{{\mathcal {G}}(\sigma , \sigma ')}(e)}. \end{aligned}$$

#### Proof

Since $\sigma ''$ is irreducible, by Lemma 5.3, the support of $\sigma ''$ is a connected set ${\mathcal {G}}(\sigma '',0)$.

Since $\sigma '' \le \sigma$, we have $\sigma |_{{{\,\textrm{supp}\,}}\sigma ''} = \sigma ''$, and hence it follows that the support of $\sigma ''$ is a connected set in ${\mathcal {G}}(\sigma , \sigma ').$

Consequently, since $e \in {{\,\textrm{supp}\,}}\sigma '',$ we have

5.2 $$\begin{aligned} {{\,\textrm{supp}\,}}\sigma '' \subseteq {\mathcal {C}}_{{\mathcal {G}}(\sigma , \sigma ')}(e), \end{aligned}$$

and thus, since $\sigma |_{{{\,\textrm{supp}\,}}\sigma ''} = \sigma '',$ it follows that

$$\begin{aligned} (\sigma |_{{\mathcal {C}}_{{\mathcal {G}}(\sigma ,\sigma ')}(e)})|_{{{\,\textrm{supp}\,}}\sigma ''} = \sigma |_{{{\,\textrm{supp}\,}}\sigma ''} = \sigma ''. \end{aligned}$$

For (5.1) to follow, it thus remains to show that5.3If $d\sigma '' = 0$, then this immediately follows. Hence, assume that $d\sigma '' \ne 0$, and let $p \in {{\,\textrm{supp}\,}}d\sigma ''.$ Since $p \in {{\,\textrm{supp}\,}}d\sigma '',$ there must exist at least one $e' \in \partial p$ with $\sigma ''(e') \ne 0.$

Since $e' \in {{\,\textrm{supp}\,}}\sigma ''$, it follows from (5.2) that $e' \in {\mathcal {C}}_{{\mathcal {G}}(\sigma ,\sigma ')}(e).$ Since $e' \in \partial p$, it follows from the definition of ${\mathcal {C}}_{{\mathcal {G}}(\sigma ,\sigma ')}(e)$ that any edge $e'' \in \partial p$ with $\sigma (e'') \ne 0$ is also a member of ${\mathcal {C}}_{{\mathcal {G}}(\sigma ,\sigma ')}(e).$ Consequently, we must have $\sigma (e'') = \sigma |_{{\mathcal {C}}_{{\mathcal {G}}(\sigma ,\sigma ')}(e)}(e'')$ for all $e'' \in \partial p,$ and hence

$$\begin{aligned} d\sigma |_{{\mathcal {C}}_{{\mathcal {G}}(\sigma ,\sigma ')}(e)} (p) = d\sigma (p). \end{aligned}$$

Since $\sigma '' \le \sigma$ and $p \in {{\,\textrm{supp}\,}}d\sigma ''$, we also have $d\sigma ''(p) = d\sigma (p),$ and hence we conclude that

$$\begin{aligned} d\sigma |_{{\mathcal {C}}_{{\mathcal {G}}(\sigma ,\sigma ')}(e)} (p) = d\sigma ''(p). \end{aligned}$$

Since this holds for any $p \in {{\,\textrm{supp}\,}}d\sigma '',$ we obtain (5.3).

This concludes the proof. $\square$

#### Lemma 5.5

Let $\sigma ,\sigma ' \in \Omega ^1(B_N,G)$, and let $E \subseteq C_1(B_N).$ Assume that $\sigma '' \le \sigma$ is non-trivial and irreducible, and that ${{\,\textrm{supp}\,}}\sigma '' \cap {\mathcal {C}}_{{\mathcal {G}}(\sigma ,\sigma ')}(E) \ne \emptyset$. Then $\sigma '' \le \sigma |_{{\mathcal {C}}_{{\mathcal {G}}(\sigma ,\sigma ')}(E)}.$

#### Proof

Fix some $$e \in {{\,\textrm{supp}\,}}\sigma '' \cap {\mathcal {C}}_{{\mathcal {G}}(\sigma ,\sigma ')}(E).$$

Since $\sigma ''$ is irreducible and $e \in {{\,\textrm{supp}\,}}\sigma '',$ it follows from Lemma 5.4 that $\sigma '' \le \sigma |_{{\mathcal {C}}_{{\mathcal {G}}(\sigma ,\sigma ')}(e)}.$

Next, since $e \in {\mathcal {C}}_{{\mathcal {G}}(\sigma ,\sigma ')}(E)$, we have ${\mathcal {C}}_{{\mathcal {G}}(\sigma ,\sigma ')}(e) \subseteq {\mathcal {C}}_{{\mathcal {G}}(\sigma ,\sigma ')} (E)$, and hence, by Lemma 5.2, it follows that

$$\begin{aligned} \sigma |_{{\mathcal {C}}_{{\mathcal {G}}(\sigma ,\sigma ')}(e)} = (\sigma |_{{\mathcal {C}}_{{\mathcal {G}}(\sigma ,\sigma ')}(E)})|_{{\mathcal {C}}_{{\mathcal {G}}(\sigma ,\sigma ')}(e)} \le \sigma |_{{\mathcal {C}}_{{\mathcal {G}}(\sigma ,\sigma ')}(E)}. \end{aligned}$$

Since $\sigma '' \le \sigma |_{{\mathcal {C}}_{{\mathcal {G}}(\sigma ,\sigma ')}(e)}$, using Lemma 2.3 (iii), we thus conclude that $\sigma '' \le \sigma |_{{\mathcal {C}}_{{\mathcal {G}}(\sigma ,\sigma ')}(E)}.$ $\square$

#### Lemma 5.6

Let $\sigma ,\sigma ' \in \Omega ^1(B_N,G)$, let $E \subseteq C_1(B_N),$ and assume that $\sigma '' \le \sigma$ is non-trivial and irreducible. Then either $\sigma '' \le \sigma |_{{\mathcal {C}}_{{\mathcal {G}}(\sigma ,\sigma ')}(E)}$, or $\sigma '' \le \sigma |_{C_1(B_N)\smallsetminus {\mathcal {C}}_{{\mathcal {G}}(\sigma ,\sigma ')}(E)}.$

#### Proof

If ${{\,\textrm{supp}\,}}\sigma '' \cap {\mathcal {C}}_{{\mathcal {G}}(\sigma ,\sigma ')}(E) \ne \emptyset$, then, by Lemma 5.5, we have $\sigma '' \le \sigma |_{{\mathcal {C}}_{{\mathcal {G}}(\sigma ,\sigma ')}(E)}$, and hence the desired conclusion holds in this case.

Now instead assume that ${{\,\textrm{supp}\,}}\sigma '' \cap {\mathcal {C}}_{{\mathcal {G}}(\sigma ,\sigma ')}(E) = \emptyset ,$ and note that this implies that ${{\,\textrm{supp}\,}}\sigma '' \subseteq C_1(B_N)\smallsetminus {\mathcal {C}}_{{\mathcal {G}}(\sigma ,\sigma ')}(E).$

Define

$$\begin{aligned} E' \, {:}{=}\, {{\,\textrm{supp}\,}}\sigma |_{C_1(B_N)\smallsetminus {\mathcal {C}}_{{\mathcal {G}}(\sigma ,\sigma ')}(E)} \cup {{\,\textrm{supp}\,}}\sigma '|_{C_1(B_N)\smallsetminus {\mathcal {C}}_{{\mathcal {G}}(\sigma ,\sigma ')}(E)}. \end{aligned}$$

Then, since $\sigma '' \le \sigma$ and ${{\,\textrm{supp}\,}}\sigma '' \subseteq C_1(B_N)\smallsetminus {\mathcal {C}}_{{\mathcal {G}}(\sigma ,\sigma ')}(E),$ we have ${{\,\textrm{supp}\,}}\sigma '' \subseteq E'.$ Consequently, by Lemma 5.5, we have $\sigma '' \le \sigma |_{{\mathcal {C}}_{{\mathcal {G}}(\sigma ,\sigma ')}(E')}.$ Since

$$\begin{aligned} \sigma |_{C_1(B_N)\smallsetminus {\mathcal {C}}_{{\mathcal {G}}(\sigma ,\sigma ')}(E)} = \sigma |_{{\mathcal {C}}_{{\mathcal {G}}(\sigma ,\sigma ')}(E')}, \end{aligned}$$

we obtain $\sigma '' \le \sigma |_{C_1(B_N)\smallsetminus {\mathcal {C}}_{{\mathcal {G}}(\sigma ,\sigma ')}(E)},$ and hence the desired conclusion holds also in this case.

This completes the proof. $\square$

### The set $E_{E_0,{\hat{\sigma }},{\hat{\sigma }}'}$

We now define a set which we will need for the definitions of the couplings in Sects. 5.3 and 5.4

#### Definition 5.7

For $\sigma ,\sigma ' \in \Omega ^1(B_N,G)$ and $E_0 \subseteq C_1(B_N)$, define

5.4 $$\begin{aligned} \begin{aligned} E_{E_0,\sigma ,\sigma '} \, {:}{=}\, {\mathcal {C}}_{{\mathcal {G}}(\sigma , \sigma ')} \bigl (&E_0 \cup \{ e \in {{\,\textrm{supp}\,}}\sigma :d\sigma |_{\pm {{\,\textrm{supp}\,}}{\hat{\partial }} e} \ne 0 \} \\&\cup \{ e \in {{\,\textrm{supp}\,}}\sigma ' :d\sigma '|_{\pm {{\,\textrm{supp}\,}}{\hat{\partial }} e} \ne 0 \} \bigr ). \end{aligned} \end{aligned}$$

#### Lemma 5.8

Let $\sigma ,\sigma ' \in \Omega ^1(B_N,G)$, and let $E_0 \subseteq C_1(B_N).$

Then

(i) $$d( \sigma |_{E_{E_0,\sigma ,\sigma '}}) = d \sigma ,$$ and
(ii) $$d( \sigma |_{C_1(B_N)\smallsetminus E_{E_0,\sigma ,\sigma '}}) = 0.$$

#### Proof

To simplify notation, let $E \, {:}{=}\, E_{E_0,\sigma ,\sigma '}$.

By Lemma 5.2, applied with $\sigma$, $\sigma '$, and *E*, we then have $\sigma |_{E} \le \sigma$, $\sigma '|_{E} \le \sigma '$, $\sigma |_{C_1(B_N) \smallsetminus E} \le \sigma$ and $\sigma '|_{C_1(B_N) \smallsetminus E} \le \sigma '$.

We now show that $d( \sigma |_{E} ) = d \sigma$. Since $\sigma |_{E} \le \sigma$, it suffices to show that $d(\sigma |_{E})(p) \ne 0$ whenever $d \sigma (p) \ne 0$. To this end, assume that $d \sigma (p) \ne 0$. Then the set ${{\,\textrm{supp}\,}}\partial p \cap {{\,\textrm{supp}\,}}\sigma$ must be non-empty. Fix one edge $e \in {{\,\textrm{supp}\,}}\partial p \cap {{\,\textrm{supp}\,}}\sigma$. Recalling the definition of *E*, we see that $e \in E$, and hence any edge $e' \in {{\,\textrm{supp}\,}}\partial p\smallsetminus \{ e \}$ must satisfy either $\sigma '(e') = \sigma (e') = 0$, or $e' \in E$.

Consequently, $\sigma |_{E}(\partial p) = \sigma (\partial p)$, and hence

$$\begin{aligned} d \sigma |_{E}(p) = \sigma |_{E}(\partial p) = \sigma (\partial p) = d \sigma ( p) \end{aligned}$$

as desired. This concludes the proof of (i).

To see that (ii) holds, note simply that, using (i), we have

$$\begin{aligned} d( \sigma |_{C_1(B_N)\smallsetminus E}) = d( \sigma - \sigma |_{E}) = d \sigma -d( \sigma |_{E}) = d \sigma - d \sigma = 0, \end{aligned}$$

and hence (ii) holds. This concludes the proof. $\square$

#### Lemma 5.9

Let ${\hat{\sigma }},{\hat{\sigma }}' \in \Omega ^1(B_N,G)$, and let $E_0 \subseteq C_1(B_N).$

Further, either let

$$\begin{aligned} {\left\{ \begin{array}{ll} \sigma \, {:}{=}\, \, {\hat{\sigma }}|_{E_{E_0,{\hat{\sigma }},{\hat{\sigma }}'}} + {\hat{\sigma }}'|_{C_1(B_N) \smallsetminus E_{E_0,{\hat{\sigma }},{\hat{\sigma }}'}} \\ \sigma ' \, {:}{=}\, \, {\hat{\sigma }}'. \end{array}\right. } \end{aligned}$$

or let

$$\begin{aligned} {\left\{ \begin{array}{ll} \sigma \, {:}{=}\, \, {\hat{\sigma }}|_{E_{E_0,{\hat{\sigma }},{\hat{\sigma }}'}} + {\hat{\sigma }}'|_{C_1(B_N) \smallsetminus E_{E_0,{\hat{\sigma }},{\hat{\sigma }}'}} \\ \sigma ' \, {:}{=}\, \,\hat{\sigma }'|_{E_{E_0,{\hat{\sigma }},{\hat{\sigma }}'}} + {\hat{\sigma }}|_{C_1(B_N) \smallsetminus E_{E_0,{\hat{\sigma }},{\hat{\sigma }}'}}. \end{array}\right. } \end{aligned}$$

Then $$ E_{E_0,\sigma , \sigma '} = E_{E_0,\hat{\sigma },{\hat{\sigma }}'}$$.

#### Proof

By Lemma 5.8, we have $d{\hat{\sigma }}|_{E_{E_0,\sigma ,\sigma '}} = d{\hat{\sigma }},$ and $d {\hat{\sigma }}'|_{C_1(B_N)\smallsetminus E_{E_0,{\hat{\sigma }},{\hat{\sigma }}'}} = 0$ and hence

$$\begin{aligned} d\sigma = d({\hat{\sigma }}|_{E_{E_0,{\hat{\sigma }},{\hat{\sigma }}'}} + \hat{\sigma }'|_{C_1(B_N)\smallsetminus E_{E_0,\sigma ,\sigma '}})  &   = d(\hat{\sigma }|_{E_{E_0,{\hat{\sigma }},{\hat{\sigma }}'}}) + d(\hat{\sigma }'|_{C_1(B_N)\smallsetminus E_{E_0,\sigma ,\sigma '}}) \\    &   = d{\hat{\sigma }} + 0 = d{\hat{\sigma }}. \end{aligned}$$

If $e \in {{\,\textrm{supp}\,}}{\hat{\sigma }}$ is such that $d{\hat{\sigma }}|_{\pm {{\,\textrm{supp}\,}}{\hat{\partial }} e} \ne 0,$ then $e \in E_{E_0,{\hat{\sigma }},{\hat{\sigma }}'}$, and thus $\sigma (e) = \hat{\sigma }(e),$ implying in particular that $e \in {{\,\textrm{supp}\,}}\sigma .$ Since $d\sigma = d{\hat{\sigma }},$ it follows that $d\sigma |_{\pm {{\,\textrm{supp}\,}}{\hat{\partial }} e} \ne 0,$ and hence

$$\begin{aligned} \{ e \in {{\,\textrm{supp}\,}}{\hat{\sigma }} :d{\hat{\sigma }}|_{\pm {{\,\textrm{supp}\,}}{\hat{\partial }} e} \ne 0 \} \subseteq \{ e \in {{\,\textrm{supp}\,}}\sigma :d\sigma |_{\pm {{\,\textrm{supp}\,}}{\hat{\partial }} e} \ne 0 \}. \end{aligned}$$

Analogously, we obtain

$$\begin{aligned} \{ e \in {{\,\textrm{supp}\,}}{\hat{\sigma }}' :d{\hat{\sigma }}'|_{\pm {{\,\textrm{supp}\,}}{\hat{\partial }} e} \ne 0 \} \subseteq \{ e \in {{\,\textrm{supp}\,}}\sigma ' :d\sigma '|_{\pm {{\,\textrm{supp}\,}}{\hat{\partial }} e} \ne 0 \}. \end{aligned}$$

Noting that ${\mathcal {G}}({\hat{\sigma }},{\hat{\sigma }}') = {\mathcal {G}}(\sigma ,\sigma '),$ we thus obtain

$$\begin{aligned} \begin{aligned}&E_{E_0,{\hat{\sigma }}, {\hat{\sigma }}'} = {\mathcal {C}}_{{\mathcal {G}}(\hat{\sigma },{\hat{\sigma }}')} \bigl (E_0 \cup \{ e \in {{\,\textrm{supp}\,}}{\hat{\sigma }} :d{\hat{\sigma }}|_{\pm {{\,\textrm{supp}\,}}\partial e} \ne 0 \} \cup \{ e \in {{\,\textrm{supp}\,}}{\hat{\sigma }}' :d{\hat{\sigma }}'|_{\pm {{\,\textrm{supp}\,}}\partial e} \ne 0 \} \bigr )\\&\qquad \subseteq {\mathcal {C}}_{{\mathcal {G}}( \sigma , \sigma ')} \bigl (E_0 \cup \{ e \in {{\,\textrm{supp}\,}}\sigma :d \sigma |_{\pm {{\,\textrm{supp}\,}}\partial e} \ne 0 \} \cup \\&\qquad \{ e \in {{\,\textrm{supp}\,}}\sigma ' :d \sigma '|_{\pm {{\,\textrm{supp}\,}}\partial e} \ne 0 \} \bigr ) = E_{E_0, \sigma , \sigma '}. \end{aligned} \end{aligned}$$

For the other direction, assume that $e \in {{\,\textrm{supp}\,}}\sigma$ is such that $d\sigma |_{\pm {{\,\textrm{supp}\,}}{\hat{\partial }} e} \ne 0.$ Then $\sigma (e)\ne 0$, and there must exist $p \in {\hat{\partial }} e$ such that $d\sigma (p) \ne 0$.

Since $d{\hat{\sigma }} = d\sigma$, it follows that $d{\hat{\sigma }}(p) \ne 0$. Consequently, there must exist $e' \in \partial p$ such that ${\hat{\sigma }}(e') \ne 0$. For any such edge $e'$, we have $d{\hat{\sigma }}|_{\pm {{\,\textrm{supp}\,}}{\hat{\partial }} e'} \ne 0,$ and hence $e' \in E_{E_0,{\hat{\sigma }},{\hat{\sigma }}'}.$ In particular, this implies that $\sigma (e') = {\hat{\sigma }}(e') \ne 0,$ and hence $e \in E_{E_0,{\hat{\sigma }},{\hat{\sigma }}'}.$ Consequently,

$$\begin{aligned} \{ e \in {{\,\textrm{supp}\,}}\sigma :d\sigma |_{\pm {{\,\textrm{supp}\,}}\hat{\partial }e} \ne 0 \} \subseteq E_{E_0,{\hat{\sigma }},{\hat{\sigma }}'}. \end{aligned}$$

Analogously, we also obtain

$$\begin{aligned} \{ e \in {{\,\textrm{supp}\,}}\sigma ' :d\sigma '|_{\pm {{\,\textrm{supp}\,}}\hat{\partial }e} \ne 0 \} \subseteq E_{E_0,{\hat{\sigma }},{\hat{\sigma }}'}. \end{aligned}$$

Again recalling that ${\mathcal {G}}({\hat{\sigma }},{\hat{\sigma }}') = {\mathcal {G}}(\sigma ,\sigma '),$ we thus obtain

$$\begin{aligned} \begin{aligned}&E_{E_0, \sigma , \sigma '} = {\mathcal {C}}_{{\mathcal {G}}( \sigma , \sigma ')} \bigl (E_0 \cup \{ e \in {{\,\textrm{supp}\,}}\sigma :d \sigma |_{\pm {{\,\textrm{supp}\,}}\partial e} \ne 0 \} \\&\qquad \cup \{ e \in {{\,\textrm{supp}\,}}\sigma ' :d \sigma '|_{\pm {{\,\textrm{supp}\,}}\partial e} \ne 0 \} \bigr ) \\  &\qquad \subseteq {\mathcal {C}}_{{\mathcal {G}}({\hat{\sigma }},{\hat{\sigma }}')} \bigl (E_0 \cup E_{E_0,{\hat{\sigma }},{\hat{\sigma }}'} \cup \{ e \in {{\,\textrm{supp}\,}}{\hat{\sigma }}' :d{\hat{\sigma }}'|_{\pm {{\,\textrm{supp}\,}}\partial e} \ne 0 \} \bigr ) = E_{E_0,{\hat{\sigma }}, {\hat{\sigma }}'}. \end{aligned} \end{aligned}$$

This concludes the proof. $\square$

#### Lemma 5.10

Let $\sigma , \sigma ' \in \Omega ^1(B_N,G)$, let $E_0 \subseteq C_1(B_N)$, and let $e \in C_1(B_N).$ Then $e\in E_{E_0,\sigma , \sigma '}$ if and only if one of the following holds.

(i) $$d(\sigma |_{{\mathcal {C}}_{{\mathcal {G}}(\sigma , \sigma ')}(e)}) \ne 0$$
(ii) $$d(\sigma ' |_{{\mathcal {C}}_{{\mathcal {G}}(\sigma , \sigma ')}(e)}) \ne 0$$
(iii) $${\mathcal {C}}_{{\mathcal {G}}(\sigma , \sigma ')}(e) \cap E_0 \ne \emptyset $$

#### Proof

Suppose first that $e\in E_{E_0,\sigma , \sigma '}$.

By the definition of ${\mathcal {C}}_{{\mathcal {G}}(\sigma ,\sigma ')}(e)$, there exists an edge $e' \in {\mathcal {C}}_{{\mathcal {G}}(\sigma ,\sigma ')}(e)$ such that

$$\begin{aligned} e' \in E_0 \cup \{ e'' \in {{\,\textrm{supp}\,}}\sigma :d\sigma |_{\pm {{\,\textrm{supp}\,}}{\hat{\partial }} e''} \ne 0\}\cup \{ e'' \in {{\,\textrm{supp}\,}}\sigma ' :d\sigma '|_{\pm {{\,\textrm{supp}\,}}{\hat{\partial }} e''} \ne 0\}. \end{aligned}$$

If $e' \in E_0$, then $e' \in {\mathcal {C}}_{{\mathcal {G}}(\sigma ,\sigma ')}(e) \cap E_0,$ and hence ${\mathcal {C}}_{{\mathcal {G}}(\sigma ,\sigma ')}(e) \cap E_0 \ne \emptyset$. If $e' \notin E_0$, then, by symmetry, we can assume that $e' \in \{ e'' \in {{\,\textrm{supp}\,}}\sigma :d\sigma |_{\pm {{\,\textrm{supp}\,}}\hat{\partial }e''} \ne 0 \}.$ In this case, we infer that there exists a plaquette $p \in \hat{\partial } e'$ such that $d\sigma (p) \ne 0$. Since $e' \in {\mathcal {C}}_{{\mathcal {G}}(\sigma ,\sigma ')}(e)$, we have ${{\,\textrm{supp}\,}}\sigma \cap {{\,\textrm{supp}\,}}\partial p \subseteq {\mathcal {C}}_{{\mathcal {G}}(\sigma ,\sigma ')}(e)$, and so $d(\sigma |_{{\mathcal {C}}_{{\mathcal {G}}(\sigma ,\sigma ')}(e)})(p) = d\sigma (p) \ne 0$.

For the other direction, assume first that ${\mathcal {C}}_{{\mathcal {G}}(\sigma , \sigma ')}(e) \cap E_0 \ne \emptyset .$ Then there is $e' \in E_0$ such that $e' \in {\mathcal {C}}_{{\mathcal {G}}(\sigma , \sigma ')}(e).$ Since $e' \in {\mathcal {C}}_{{\mathcal {G}}(\sigma , \sigma ')}(e)$ we must also have $e \in {\mathcal {C}}_{{\mathcal {G}}(\sigma , \sigma ')}(e'),$ which is a subset of $E_{E_0,\sigma ,\sigma '}$ since $e' \in E_0$. Next, assume instead that there is a plaquette $p \in C_2(B_N)$ such that $d(\sigma |_{{\mathcal {C}}_{{\mathcal {G}}(\sigma ,\sigma ')}(e)})(p)\ne 0$. Then there exists an edge $e' \in \partial p$ with $\sigma (e') \ne 0$ and $e' \in {\mathcal {C}}_{{\mathcal {G}}(\sigma ,\sigma ')}(e)$. Thus ${{\,\textrm{supp}\,}}\sigma \cap {{\,\textrm{supp}\,}}\partial p \subseteq {\mathcal {C}}_{{\mathcal {G}}(\sigma ,\sigma ')}(e)$, and so $d\sigma (p) = d(\sigma |_{{\mathcal {C}}_{{\mathcal {G}}(\sigma ,\sigma ')}(e)})(p) \ne 0.$ In particular, it follows that $e' \in {{\,\textrm{supp}\,}}\partial p \subseteq \{ e'' \in {{\,\textrm{supp}\,}}\sigma :d\sigma |_{\pm {{\,\textrm{supp}\,}}{\hat{\partial }} e''} \ne 0 \}.$ Consequently, we must have $e' \in \{ e'' \in {{\,\textrm{supp}\,}}\sigma :d\sigma |_{\pm {{\,\textrm{supp}\,}}\hat{\partial }e'' } \ne 0 \}$, and hence $e' \in E_{\sigma ,\sigma '}.$ Since $e' \in {\mathcal {C}}_{{\mathcal {G}}_{\sigma ,\sigma '}(e)}$, we thus have $e \in {\mathcal {C}}_{{\mathcal {G}}_{\sigma ,\sigma '}(e')} \subseteq E_{E_0,\sigma ,\sigma '}$ as desired. Using symmetry, this concludes the proof. $\square$

#### Lemma 5.11

Let $$\beta _1,\beta _2 \in [0,\infty ]$$, $$\kappa \ge 0$$, $$E_0 \subseteq C_1(B_N),$$ and $$ \sigma ,{\hat{\sigma }},{\hat{\sigma }}' \in \Omega ^1(B_N,G)$$.

Then

5.5 $$\begin{aligned} \begin{aligned}&\varphi _{\beta _1,\kappa }({\hat{\sigma }}) \varphi _{\beta _2,\kappa }(\hat{\sigma }') \cdot \mathbb {1} \bigl ( {\hat{\sigma }}|_{E_{E_0, {\hat{\sigma }}, {\hat{\sigma }}'}} + {\hat{\sigma }}'|_{C_1(B_N)\smallsetminus E_{E_0, \hat{\sigma }, {\hat{\sigma }}'}} = \sigma \bigr ) \\  &\quad = \varphi _{\beta _1,\kappa }(\sigma )\sum _{\sigma ' \in \Omega ^1(B_N,G)}\varphi _{\beta _2,\kappa }(\sigma ') \cdot \mathbb {1}\bigl ( {\hat{\sigma }} = \sigma |_{E_{E_0, \sigma , \sigma '}}+ \sigma '|_{C_1(B_N)\smallsetminus E_{E_0, \sigma , \sigma '}} \bigr ) \\  &\qquad \cdot \mathbb {1}\bigl ( {\hat{\sigma }}' = \sigma ' |_{E_{E_0, \sigma , \sigma '}} + \sigma |_{C_1(B_N)\smallsetminus E_{E_0, \sigma , \sigma '}} \bigr ). \end{aligned} \end{aligned}$$

#### Proof

By Lemma 5.2, we have ${\hat{\sigma }} |_{E_{E_0, {\hat{\sigma }}, {\hat{\sigma }}'}} \le {\hat{\sigma }}$ and ${\hat{\sigma }}' |_{E_{E_0, {\hat{\sigma }}, {\hat{\sigma }}'}} \le {\hat{\sigma }}'$ and hence, by Lemma 4.1,

5.6 $$\begin{aligned} \varphi _{\beta _1,\kappa }({\hat{\sigma }}) = \varphi _{\beta _1,\kappa }\bigl ( {\hat{\sigma }} |_{E_{E_0, {\hat{\sigma }}, {\hat{\sigma }}'}} \bigr ) \varphi _{\beta _1,\kappa }\bigl ( {\hat{\sigma }} |_{C_1(B_N)\smallsetminus E_{E_0, {\hat{\sigma }}, {\hat{\sigma }}'}} \bigr ) \end{aligned}$$

and

5.7 $$\begin{aligned} \varphi _{\beta _2,\kappa }({\hat{\sigma }}') = \varphi _{\beta _2,\kappa }\bigl ( {\hat{\sigma }}' |_{E_{E_0, {\hat{\sigma }}, {\hat{\sigma }}'}} \bigr ) \varphi _{\beta _2,\kappa }\bigl ( {\hat{\sigma }}' |_{C_1(B_N)\smallsetminus E_{E_0, {\hat{\sigma }}, {\hat{\sigma }}'}} \bigr ). \end{aligned}$$

Next, by Lemma 5.8, we have

$$\begin{aligned} d({\hat{\sigma }} |_{C_1(B_N)\smallsetminus E_{E_0, {\hat{\sigma }}, {\hat{\sigma }}'}}) = 0, \end{aligned}$$

and hence

$$\begin{aligned} \varphi _{\beta _1,\kappa } \bigl ( {\hat{\sigma }} |_{C_1(B_N)\smallsetminus E_{E_0, {\hat{\sigma }}, {\hat{\sigma }}'}}\bigr ) = \varphi _{\kappa } \bigl ( {\hat{\sigma }} |_{C_1(B_N)\smallsetminus E_{E_0, {\hat{\sigma }}, {\hat{\sigma }}'}} \bigr ) = \varphi _{\beta _2,\kappa } \bigl ( {\hat{\sigma }} |_{C_1(B_N)\smallsetminus E_{E_0, {\hat{\sigma }}, {\hat{\sigma }}'}}\bigr ). \end{aligned}$$

Since ${\hat{\sigma }}'|_{E_{E_0, {\hat{\sigma }},{\hat{\sigma }}'}}$ and ${\hat{\sigma }}|_{C_1(B_N)\smallsetminus E_{E_0, {\hat{\sigma }},{\hat{\sigma }}'}}$ have disjoint supports, it also follows that

$$\begin{aligned} {\hat{\sigma }}'|_{E_{E_0, {\hat{\sigma }},{\hat{\sigma }}'}} \le {\hat{\sigma }}'|_{E_{E_0, {\hat{\sigma }},{\hat{\sigma }}'}} + {\hat{\sigma }}|_{C_1(B_N)\smallsetminus E_{E_0, {\hat{\sigma }},{\hat{\sigma }}'}}. \end{aligned}$$

Thus, by Lemma 4.1, it follows that

5.8 $$\begin{aligned} \varphi _{\beta _2,\kappa }\bigl ({\hat{\sigma }}'|_{E_{E_0, \hat{\sigma },{\hat{\sigma }}'}}\bigr ) \varphi _{\beta _1,\kappa }\bigl ({\hat{\sigma }}|_{C_1(B_N)\smallsetminus E_{E_0, {\hat{\sigma }},{\hat{\sigma }}'}}\bigr ) = \varphi _{\beta _2,\kappa }\bigl ({\hat{\sigma }}'|_{E_{E_0, {\hat{\sigma }},{\hat{\sigma }}'}} + {\hat{\sigma }}|_{C_1(B_N)\smallsetminus E_{E_0, {\hat{\sigma }},{\hat{\sigma }}'}}\bigr ).\nonumber \\ \end{aligned}$$

By symmetry, we also have

5.9 $$\begin{aligned} \varphi _{\beta _1,\kappa }\bigl ({\hat{\sigma }}|_{E_{E_0, {\hat{\sigma }},{\hat{\sigma }}'}}\bigr ) \varphi _{\beta _2,\kappa }\bigl ({\hat{\sigma }}'|_{C_1(B_N)\smallsetminus E_{E_0, {\hat{\sigma }},{\hat{\sigma }}'}}\bigr ) = \varphi _{\beta _1,\kappa }\bigl ({\hat{\sigma }}|_{E_{E_0, {\hat{\sigma }},{\hat{\sigma }}'}} + {\hat{\sigma }}'|_{C_1(B_N)\smallsetminus E_{E_0, {\hat{\sigma }},{\hat{\sigma }}'}}\bigr ).\nonumber \\ \end{aligned}$$

Combining (5.6), (5.7), (5.8), and (5.9), it follows that

5.10 $$\begin{aligned}&\varphi _{\beta _1,\kappa }( {\hat{\sigma }}) \varphi _{\beta _2,\kappa }( {\hat{\sigma }}') \cdot \mathbb {1} \bigl ( {\hat{\sigma }}|_{E_{E_0, {\hat{\sigma }}, {\hat{\sigma }}'}} + {\hat{\sigma }}'|_{C_1(B_N)\smallsetminus E_{E_0, {\hat{\sigma }}, {\hat{\sigma }}'}} = \sigma \bigr ) \nonumber \\  &\quad = \varphi _{\beta _1,\kappa }\bigl ( {\hat{\sigma }} |_{E_{E_0, {\hat{\sigma }}, {\hat{\sigma }}'}} + {\hat{\sigma }}' |_{C_1(B_N)\smallsetminus E_{E_0, {\hat{\sigma }}, {\hat{\sigma }}'}} \bigr ) \varphi _{\beta _2,\kappa }\bigl ( {\hat{\sigma }}' |_{E_{E_0, {\hat{\sigma }}, {\hat{\sigma }}'}} + {\hat{\sigma }} |_{C_1(B_N)\smallsetminus E_{E_0, {\hat{\sigma }}, {\hat{\sigma }}'}} \bigr ) \nonumber \\  &\qquad \cdot \mathbb {1} \bigl ( {\hat{\sigma }}|_{E_{E_0, {\hat{\sigma }}, {\hat{\sigma }}'}} + {\hat{\sigma }}'|_{C_1(B_N)\smallsetminus E_{E_0, {\hat{\sigma }}, {\hat{\sigma }}'}} = \sigma \bigr ) \nonumber \\  &\quad = \varphi _{\beta _1,\kappa }(\sigma ) \varphi _{\beta _2,\kappa }\bigl ( {\hat{\sigma }}' |_{E_{E_0, {\hat{\sigma }}, {\hat{\sigma }}'}} + {\hat{\sigma }} |_{C_1(B_N)\smallsetminus E_{E_0, {\hat{\sigma }}, {\hat{\sigma }}'}} \bigr ) \cdot \mathbb {1} \bigl ( {\hat{\sigma }}|_{E_{E_0, {\hat{\sigma }}, {\hat{\sigma }}'}} + {\hat{\sigma }}'|_{C_1(B_N)\smallsetminus E_{E_0, {\hat{\sigma }}, {\hat{\sigma }}'}} = \sigma \bigr ) \nonumber \\  &\quad = \varphi _{\beta _1,\kappa }(\sigma )\sum _{\sigma ' \in \Omega ^1(B_N,G)}\varphi _{\beta _2,\kappa }(\sigma ') \cdot \mathbb {1} \bigl ( {\hat{\sigma }}' |_{E_{E_0, {\hat{\sigma }}, {\hat{\sigma }}'}} + {\hat{\sigma }} |_{C_1(B_N)\smallsetminus E_{E_0, {\hat{\sigma }}, {\hat{\sigma }}'}} = \sigma '\bigr ) \nonumber \\  &\qquad \cdot \mathbb {1} \bigl ( {\hat{\sigma }}|_{E_{E_0, {\hat{\sigma }}, {\hat{\sigma }}'}} + {\hat{\sigma }}'|_{C_1(B_N)\smallsetminus E_{E_0, {\hat{\sigma }}, {\hat{\sigma }}'}} = \sigma \bigr ). \end{aligned}$$

Now fix $\sigma ' \in \Omega ^1(B_N,G)$ and assume that

$$\begin{aligned} {\left\{ \begin{array}{ll} \sigma = {\hat{\sigma }}|_{E_{E_0, {\hat{\sigma }}, {\hat{\sigma }}'}} + {\hat{\sigma }}'|_{C_1(B_N)\smallsetminus E_{E_0, {\hat{\sigma }}, {\hat{\sigma }}'}} \\ \sigma ' = {\hat{\sigma }}' |_{E_{E_0, {\hat{\sigma }}, {\hat{\sigma }}'}} + {\hat{\sigma }} |_{C_1(B_N)\smallsetminus E_{E_0, {\hat{\sigma }}, {\hat{\sigma }}'}} . \end{array}\right. } \end{aligned}$$

By Lemma 5.9, we have $E_{E_0, {\hat{\sigma }}, {\hat{\sigma }}'} = E_{E_0, \sigma ,\sigma '}.$ Since $\sigma |_{E_{E_0, {\hat{\sigma }}, {\hat{\sigma }}'}} = {\hat{\sigma }}|_{E_{E_0, {\hat{\sigma }}, {\hat{\sigma }}'}}$ and $\sigma ' |_{C_1(B_N)\smallsetminus E_{E_0, {\hat{\sigma }}, {\hat{\sigma }}'}} = {\hat{\sigma }} |_{C_1(B_N)\smallsetminus E_{E_0, {\hat{\sigma }}, {\hat{\sigma }}'}},$ it follows that

$$\begin{aligned} {\hat{\sigma }} = \sigma |_{E_{E_0, {\hat{\sigma }}, {\hat{\sigma }}'}} + \sigma '|_{C_1(B_N)\smallsetminus E_{E_0, {\hat{\sigma }}, {\hat{\sigma }}'}} = \sigma |_{E_{E_0, \sigma , \sigma '}} + \sigma '|_{C_1(B_N)\smallsetminus E_{E_0, \sigma , \sigma '}}. \end{aligned}$$

Analogously, since $\sigma '|_{E_{E_0, {\hat{\sigma }}, {\hat{\sigma }}'}} = {\hat{\sigma }}' |_{E_{E_0, {\hat{\sigma }}, {\hat{\sigma }}'}}$ and $\sigma |_{C_1(B_N)\smallsetminus E_{E_0, {\hat{\sigma }}, {\hat{\sigma }}'}} ={\hat{\sigma }}'|_{C_1(B_N)\smallsetminus E_{E_0, {\hat{\sigma }}, {\hat{\sigma }}'}},$ it follows that

$$\begin{aligned} {\hat{\sigma }}' = \sigma '|_{E_{E_0, {\hat{\sigma }}, {\hat{\sigma }}'}} + \sigma |_{C_1(B_N)\smallsetminus E_{E_0, {\hat{\sigma }}, {\hat{\sigma }}'}} = \sigma '|_{E_{E_0, \sigma , \sigma '}} + \sigma |_{C_1(B_N)\smallsetminus E_{E_0, \sigma , \sigma '}}. \end{aligned}$$

This shows that for any $\sigma ' \in \Omega ^1(B_N,G),$ we have

5.11 $$\begin{aligned}&\mathbb {1} \bigl ( {\hat{\sigma }}' |_{E_{E_0, {\hat{\sigma }}, {\hat{\sigma }}'}} + {\hat{\sigma }} |_{C_1(B_N)\smallsetminus E_{E_0, {\hat{\sigma }}, {\hat{\sigma }}'}} = \sigma '\bigr ) \cdot \mathbb {1} \bigl ( {\hat{\sigma }}|_{E_{E_0, {\hat{\sigma }}, {\hat{\sigma }}'}} + {\hat{\sigma }}'|_{C_1(B_N)\smallsetminus E_{E_0, {\hat{\sigma }}, {\hat{\sigma }}'}} = \sigma \bigr ) \nonumber \\  &\qquad = \mathbb {1}\bigl ( {\hat{\sigma }} = \sigma |_{E_{E_0, \sigma , \sigma '}}+ \sigma '|_{C_1(B_N)\smallsetminus E_{E_0, \sigma , \sigma '}} \bigr ) \cdot \mathbb {1}\bigl ( {\hat{\sigma }}' = \sigma ' |_{E_{E_0, \sigma , \sigma '}} + \sigma |_{C_1(B_N)\smallsetminus E_{E_0, \sigma , \sigma '}} \bigr ). \end{aligned}$$

Combining (5.10) and (5.11), we obtain (5.5) as desired. $\square$

### A coupling between two ${\mathbb {Z}}_n$-models

In this section, we define a coupling between two copies of $\mu _{N,\infty ,\kappa }$, constructed to always agree on a given set $E_0 \subseteq C_1(B_N)$

#### Definition 5.12

. For $\kappa \ge 0,$ $\sigma ,\sigma ' \in \Omega ^1_0(B_N,G),$ $E_0 \subseteq C_1(B_N),$ and $E_{E_0, \sigma ,\sigma '} = {\mathcal {C}}_{{\mathcal {G}}_{\sigma ,\sigma '}}(E_0),$ we defineWe let ${\mathbb {E}}^{E_0}_{N,(\infty ,\kappa ),(\infty ,\kappa )}$ denote the corresponding expectation.

**Fig. 5:**
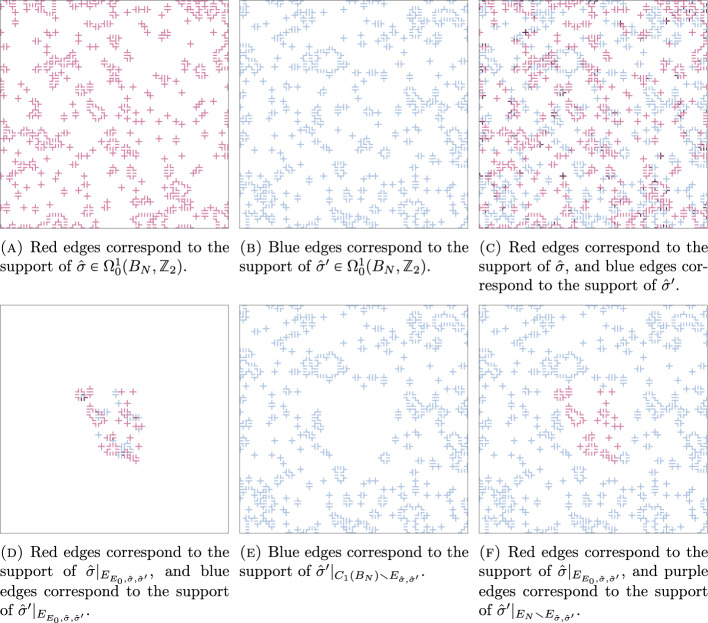
Illustration of the coupling $(\sigma , \sigma ') \sim \mu ^{E_0}_{N,(\infty ,\beta ),(\infty ,\beta )}$ defined in Definition 5.18, simulated on a 2-dimensional lattice, with $G = {\mathbb {Z}}_2$, and with $E_0 = C_1(B_{N/4}).$

#### Remark 5.13

When $\sigma ,\sigma ' \in \Omega ^1_0(B_N,G)$, then $d\sigma = d\sigma ' = 0,$ and hence the definition of $E_{E_0,\sigma ,\sigma '}$ in Definition 5.12 in consistent with (5.4).

#### Remark 5.14

By definition, if ${\hat{\sigma }}, {\hat{\sigma }}' \sim \mu _{N,\infty ,\kappa }$ are independent, and we let $\sigma \, {:}{=}\, {\hat{\sigma }}|_{E_{E_0,{\hat{\sigma }}, {\hat{\sigma }}'}} + \hat{\sigma }'|_{C_1(B_N) \smallsetminus E_{E_0, {\hat{\sigma }}, \hat{\sigma }'}}$ and $\sigma ' \, {:}{=}\, {\hat{\sigma }}'$, then $(\sigma ,\sigma ') \sim \mu ^{E_0}_{N,(\infty ,\kappa ),(\infty ,\kappa )}$.

The next result shows that the measure introduced in Definition 5.12 is indeed a coupling.

#### Proposition 5.15

Let $\kappa \ge 0$, and let $E_0 \subseteq C_1(B_N)$. Then $\mu ^{E_0}_{N,(\infty ,\kappa ),{(\infty ,\kappa })}$ is a coupling of $\mu _{N,\infty ,\kappa }$ and $\mu _{N,\infty ,\kappa }$.

#### Proof

It is immediate from the definition that if $(\sigma ,\sigma ') \sim \mu ^{E_0}_{N,(\infty ,\kappa ),(\infty ,\kappa )}$, then $\sigma ' \sim \mu _{N,\infty ,\kappa }$, and it is hence sufficient to show that $\sigma \sim \mu _{N,\infty ,\kappa }$.

To this end, fix some $\sigma \in \Omega ^1_0(B_N)$. We need to show that

$$\begin{aligned}  &   \mu _{N,\infty ,\kappa }\times \mu _{N,\infty ,\kappa } \bigl ( \bigl \{ ({\hat{\sigma }}, {\hat{\sigma }}') \in \Omega ^1_0(B_N) \times \Omega ^1_0(B_N) :{\hat{\sigma }}|_{E_{E_0,{\hat{\sigma }}, {\hat{\sigma }}'}} + {\hat{\sigma }}'|_{C_1(B_N)\smallsetminus E_{E_0, \hat{\sigma },{\hat{\sigma }}'}} \\  &   \quad = \sigma \bigr \} \bigr ) = \mu _{N,\infty ,\kappa }( \sigma ), \end{aligned}$$

or equivalently, that

5.12 $$\begin{aligned}  &   \sum _{\begin{array}{c} {\hat{\sigma }} \in \Omega ^1_0(B_N,G),\\ {\hat{\sigma }}' \in \Omega ^1_0(B_N,G) \end{array}} \varphi _{\kappa }({\hat{\sigma }}) \varphi _{\kappa }({\hat{\sigma }}') \cdot \mathbb {1} \bigl ( \hat{\sigma }|_{E_{E_0,{\hat{\sigma }},{\hat{\sigma }}'}} + \hat{\sigma }'|_{C_1(B_N)\smallsetminus E_{E_0,{\hat{\sigma }},{\hat{\sigma }}'}} = \sigma \bigr ) \nonumber \\  &   = \varphi _\kappa ( \sigma ) \sum _{ \sigma ' \in \Omega ^1_0(B_N,G)} \varphi _{\kappa } ( \sigma '). \end{aligned}$$

We now rewrite the left-hand side of (5.12) in order to see that this equality indeed holds.

To this end, note first that by Lemma 5.11, applied with $\beta _1=\beta _2=\infty$, we have

5.13 $$\begin{aligned} \begin{aligned}&\sum _{\begin{array}{c} {\hat{\sigma }} \in \Omega ^1_0(B_N,G),\\ {\hat{\sigma }}' \in \Omega ^1_0(B_N,G) \end{array}} \varphi _{\kappa }({\hat{\sigma }}) \varphi _{\kappa }({\hat{\sigma }}')\mathbb {1} \bigl ( \hat{\sigma }|_{E_{E_0, {\hat{\sigma }}, {\hat{\sigma }}'}} + \hat{\sigma }'|_{C_1(B_N)\smallsetminus E_{E_0, {\hat{\sigma }}, {\hat{\sigma }}'}} = \sigma \bigr ) \\  &\qquad = \varphi _{\kappa }( \sigma ) \sum _{\sigma ' \in \Omega ^1_0(B_N,G)} \varphi _{\kappa }(\sigma ') \cdot \sum _{\begin{array}{c} {\hat{\sigma }} \in \Omega ^1_0(B_N,G),\\ {\hat{\sigma }}' \in \Omega ^1_0(B_N,G) \end{array}} \mathbb {1}\bigl ( {\hat{\sigma }} = \sigma '|_{E_{E_0, \sigma , \sigma '}}+ \sigma |_{C_1(B_N)\smallsetminus E_{E_0, \sigma , \sigma '}} \bigr ) \\&\hspace{22em}\cdot \mathbb {1}\bigl ( {\hat{\sigma }}' = \sigma |_{E_{E_0, \sigma , \sigma '}} + \sigma ' |_{C_1(B_N)\smallsetminus E_{E_0, \sigma , \sigma '}} \bigr ). \end{aligned}\nonumber \\ \end{aligned}$$

Since $\sigma , \sigma ' \in \Omega ^1_0(B_N)$, we can apply Lemma 5.8 to see that $\sigma |_{E_{E_0, \sigma , \sigma '}} + \sigma ' |_{C_1(B_N)\smallsetminus E_{E_0, \sigma , \sigma '}} \in \Omega ^1_0(B_N,G)$ and $\sigma ' |_{E_{E_0, \sigma , \sigma '}} + \sigma |_{C_1(B_N)\smallsetminus E_{E_0, \sigma , \sigma '}} \in \Omega ^1_0(B_N,G).$

From this it follows that the double sum on the right-hand side of (5.13) is equal to 1, and hence we obtain (5.12) as desired. This completes the proof. $\square$

#### Lemma 5.16

Let $\beta ,\kappa \ge 0,$ let $E_0 \subseteq C_1(B_N),$ and let $(\sigma ,\sigma ')\in \Omega ^1_0(B_N,G) \times \Omega ^1_0(B_N,G)$ be such that $\mu ^{E_0}_{N,(\infty ,\kappa ),(\infty ,\kappa )}(\sigma , \sigma ') \ne 0$. Then $\sigma (e) = \sigma '(e)$ for all $e \in C_1(B_N) \smallsetminus E_{E_0,\sigma ,\sigma '}$.

#### Proof

Since $\mu ^{E_0}_{N,(\infty ,\kappa ),(\infty ,\kappa )}(\sigma , \sigma ') \ne 0$, by definition, there is $({\hat{\sigma }}, \hat{\sigma }') \in \Omega ^1_0(B_N,G) \times \Omega ^1_0(B_N,G)$ such that $\sigma = {\hat{\sigma }}|_{E_{E_0,{\hat{\sigma }},{\hat{\sigma }}'}} + {\hat{\sigma }}'|_{C_1(B_N) \smallsetminus E_{E_0,\hat{\sigma }, \hat{\sigma }'}}$ and $\sigma ' = {\hat{\sigma }}'$. Using Lemma 5.9, we immediately obtain the desired conclusion. $\square$

One application of the coupling introduced in Definition 5.12, which will be particularly useful to us, is the following proposition.

#### Proposition 5.17

Let $\kappa \ge 0$, and let $E_0,E_1 \subseteq C^1(B_N,{\mathbb {Z}})$ have disjoint supports. Further, let $f_0,f_1 :\Omega ^1(B_N,G) \rightarrow {\mathbb {R}}$ be such that $f_0(\sigma ) = f(\sigma |_{E_0})$ and $f_1(\sigma ) = f_1(\sigma |_{E_1})$ for all $\sigma \in \Omega ^1(B_N).$

Then5.14

We provide an upper bound on the right hand side of (5.14) in Proposition 6.4.

#### Proof of Proposition 5.17

To simplify notation, for $\sigma \in \Omega ^1(B_N,G),$ let $F(\sigma ) \, {:}{=}\, f_1(\sigma )f_2(\sigma )$. Let ${\hat{\sigma }},{\hat{\sigma }}' \sim \mu _{N,\infty ,\kappa }.$ Note that when $d{\hat{\sigma }} = d{\hat{\sigma }}' = 0$, we have $E_{E_0,{\hat{\sigma }},{\hat{\sigma }}'} = {\mathcal {C}}_{{\mathcal {G}}(\hat{\sigma },{\hat{\sigma }}') }(E_0).$

Define

$$\begin{aligned} {\left\{ \begin{array}{ll} \sigma \,{:}{=}\, {\hat{\sigma }}|_{E_{E_0,{\hat{\sigma }},{\hat{\sigma }}'}} + {\hat{\sigma }}'|_{C_1(B_N)\smallsetminus E_{E_0,{\hat{\sigma }},\hat{\sigma }'}}. \\ \sigma ' \, {:}{=}\, \, {\hat{\sigma }}'. \end{array}\right. } \end{aligned}$$

Then $(\sigma ,\sigma ') \sim \mu ^{E_0}_{N,(\infty ,\kappa ),(\infty ,\kappa )},$ and hence $\sigma ,\sigma ' \sim \mu _{N,\infty ,\kappa }$. Consequently, we have

5.15 $$\begin{aligned} {\mathbb {E}}_{N,\infty ,\kappa } \bigl [ F(\sigma ) \bigr ]&= {\mathbb {E}}^{E_0}_{N,(\infty ,\kappa ),(\infty ,\kappa )} \bigl [ F(\sigma ) \bigr ] \nonumber \\  &= {\mathbb {E}}_{N,\infty ,\kappa } \times {\mathbb {E}}_{N,\infty ,\kappa } \bigl [ F ({\hat{\sigma }}|_{E_{E_0,{\hat{\sigma }},{\hat{\sigma }}'}}\nonumber \\&\quad + \hat{\sigma }'|_{C_1(B_N)\smallsetminus E_{E_0,{\hat{\sigma }},{\hat{\sigma }}'}}) \bigr ] \nonumber \\  &= {\mathbb {E}}_{N,\infty ,\kappa } \times {\mathbb {E}}_{N,\infty ,\kappa }\bigl [ F ({\hat{\sigma }}|_{E_{E_0,\hat{\sigma },{\hat{\sigma }}'}} + {\hat{\sigma }}'|_{C_1(B_N)\smallsetminus E_{E_0,{\hat{\sigma }},{\hat{\sigma }}'}}) \cdot \nonumber \\&\quad \mathbb {1}( E_1 \cap E_{E_0,{\hat{\sigma }},{\hat{\sigma }}'}) = \emptyset ) \bigr ] \nonumber \\  &\quad + {\mathbb {E}}_{N,\infty ,\kappa }\times {\mathbb {E}}_{N,\infty ,\kappa } \bigl [ F ({\hat{\sigma }}|_{E_{E_0,{\hat{\sigma }},{\hat{\sigma }}'}} + \hat{\sigma }'|_{C_1(B_N)\smallsetminus E_{E_0,{\hat{\sigma }},{\hat{\sigma }}'}}) \cdot \nonumber \\&\quad \mathbb {1}( E_1 \cap E_{E_0,{\hat{\sigma }},{\hat{\sigma }}'}) \ne \emptyset ) \bigr ]. \end{aligned}$$

Since $E_{E_0,{\hat{\sigma }},{\hat{\sigma }}'} = {\mathcal {C}}_{{\mathcal {G}}({\hat{\sigma }},{\hat{\sigma }}')}(E_0),$ we have $( {{\,\textrm{supp}\,}}{\hat{\sigma }} \cup {{\,\textrm{supp}\,}}{\hat{\sigma }}' )\cap E_0 \subseteq E_{E_0,{\hat{\sigma }},{\hat{\sigma }}'}.$ This implies in particular that

$$\begin{aligned} ({\hat{\sigma }}|_{E_{E_0,{\hat{\sigma }}, {\hat{\sigma }}'}} + \hat{\sigma }'|_{C_1(B_N)\smallsetminus E_{E_0,{\hat{\sigma }},\hat{\sigma }'}})|_{E_0} = ({\hat{\sigma }}|_{E_{E_0,{\hat{\sigma }}, \hat{\sigma }'}})|_{E_0} + ({\hat{\sigma }}'|_{C_1(B_N)\smallsetminus E_{E_0,{\hat{\sigma }},{\hat{\sigma }}'}})|_{E_0} = {\hat{\sigma }}|_{E_0}, \end{aligned}$$

and hence

$$\begin{aligned} \begin{aligned}&f_0({\hat{\sigma }}|_{E_{E_0,{\hat{\sigma }}, {\hat{\sigma }}'}} + \hat{\sigma }'|_{C_1(B_N)\smallsetminus E_{E_0,{\hat{\sigma }},{\hat{\sigma }}'}}) = f_0({\hat{\sigma }}). \end{aligned} \end{aligned}$$

At the same time, on the event $E_1 \cap E_{E_0,{\hat{\sigma }},\hat{\sigma }'} = \emptyset ,$ for all $e \in E_1$ we have

$$\begin{aligned} \begin{aligned}&({\hat{\sigma }}|_{E_{E_0,{\hat{\sigma }},{\hat{\sigma }}'}} + \hat{\sigma }'|_{C_1(B_N)\smallsetminus E_{E_0,{\hat{\sigma }},\hat{\sigma }'}})|_{E_1} = ({\hat{\sigma }}|_{E_{E_0,{\hat{\sigma }},\hat{\sigma }'}})|_{E_1} + ({\hat{\sigma }}'|_{C_1(B_N)\smallsetminus E_{E_0,{\hat{\sigma }},{\hat{\sigma }}'}})|_{E_1}\\  &= 0 + {\hat{\sigma }}'|_{E_1}, \end{aligned} \end{aligned}$$

and hence

$$\begin{aligned} \begin{aligned}&f_1({\hat{\sigma }}|_{E_{E_0,{\hat{\sigma }},{\hat{\sigma }}'}} + \hat{\sigma }'|_{C_1(B_N)\smallsetminus E_{E_0,{\hat{\sigma }},{\hat{\sigma }}'}}) = f_1({\hat{\sigma }}'). \end{aligned} \end{aligned}$$

Consequently, on the event $E_1 \cap E_{E_0,\sigma ,\sigma '} = \emptyset ,$ we have

$$\begin{aligned} \begin{aligned}&F ({\hat{\sigma }}|_{E_{E_0,{\hat{\sigma }},{\hat{\sigma }}'}} + \hat{\sigma }'|_{C_1(B_N)\smallsetminus E_{E_0,{\hat{\sigma }},{\hat{\sigma }}'}}) =f_0 \bigl ( {\hat{\sigma }}\bigr ) f_1 \bigl ( {\hat{\sigma }}'\bigr ), \end{aligned} \end{aligned}$$

and hence

$$\begin{aligned} \begin{aligned}&{\mathbb {E}}_{N,\infty ,\kappa } \times {\mathbb {E}}_{N,\infty ,\kappa }\bigl [ F ({\hat{\sigma }}|_{E_{E_0,\hat{\sigma },{\hat{\sigma }}'}} + {\hat{\sigma }}'|_{C_1(B_N)\smallsetminus E_{E_0,{\hat{\sigma }},{\hat{\sigma }}'}}) \cdot \mathbb {1}( E_1 \cap E_{E_0,{\hat{\sigma }},{\hat{\sigma }}'} = \emptyset ) \bigr ] \\  &\quad = {\mathbb {E}}_{N,\infty ,\kappa } \times {\mathbb {E}}_{N,\infty ,\kappa }\bigl [ f_0({\hat{\sigma }}) f_1( \hat{\sigma }') \cdot \mathbb {1}( E_1 \cap E_{E_0,{\hat{\sigma }},{\hat{\sigma }}'} = \emptyset ) \bigr ] \\  &\quad = {\mathbb {E}}_{N,\infty ,\kappa } \times {\mathbb {E}}_{N,\infty ,\kappa }\bigl [ f_0({\hat{\sigma }}) f_1( \hat{\sigma }') \bigr ] - {\mathbb {E}}_{N,\infty ,\kappa } \times {\mathbb {E}}_{N,\infty ,\kappa }\bigl [ f_0({\hat{\sigma }}) f_1( \hat{\sigma }') \\  &\quad \cdot \mathbb {1}( E_1 \cap E_{E_0,{\hat{\sigma }},{\hat{\sigma }}'} \ne \emptyset ) \bigr ] \\  &\quad = {\mathbb {E}}_{N,\infty ,\kappa } \bigl [ f_0({\hat{\sigma }}) \bigr ] {\mathbb {E}}_{N,\infty ,\kappa } \bigl [ f_1( {\hat{\sigma }}') \bigr ] - {\mathbb {E}}_{N,\infty ,\kappa } \times {\mathbb {E}}_{N,\infty ,\kappa }\bigl [ f_0({\hat{\sigma }}) f_1( \hat{\sigma }') \\  &\quad \cdot \mathbb {1}( E_1 \cap E_{E_0,{\hat{\sigma }},{\hat{\sigma }}'} \ne \emptyset ) \bigr ]. \end{aligned} \end{aligned}$$

Inserting this into (5.15), we see that

$$\begin{aligned} \begin{aligned}&{\mathbb {E}}_{N,\infty ,\kappa } \bigl [ f_0(\sigma )f_1(\sigma ) \bigr ] \\  &\quad = {\mathbb {E}}_{N,\infty ,\kappa } \bigl [ f_0( {\hat{\sigma }}) \bigr ] {\mathbb {E}}_{N,\infty ,\kappa }\bigl [ f_1( {\hat{\sigma }}') \bigr ] - {\mathbb {E}}_{N,\infty ,\kappa } \times {\mathbb {E}}_{N,\infty ,\kappa }\bigl [ f_0 ( {\hat{\sigma }}) f_1( \hat{\sigma }') \\&\quad \cdot \mathbb {1}( E_1 \cap E_{E_0,{\hat{\sigma }},\hat{\sigma }'}) \ne \emptyset ) \bigr ] \\  &\qquad + {\mathbb {E}}_{N,\infty ,\kappa }\times {\mathbb {E}}_{N,\infty ,\kappa } \bigl [ F ({\hat{\sigma }}'|_{E_{E_0,{\hat{\sigma }},{\hat{\sigma }}'}} + \hat{\sigma }|_{C_1(B_N)\smallsetminus E_{E_0,{\hat{\sigma }},{\hat{\sigma }}'}}) \cdot \mathbb {1}( E_1 \cap E_{E_0,{\hat{\sigma }},{\hat{\sigma }}'} \ne \emptyset ) \bigr ]. \end{aligned} \end{aligned}$$

In particular, this implies that

$$\begin{aligned} \begin{aligned}&\Bigl | {\mathbb {E}}_{N,\infty ,\kappa } \bigl [ f_0( \sigma )f_1( \sigma ) \bigr ] - {\mathbb {E}}_{N,\infty ,\kappa } \bigl [ f_0( \hat{\sigma }) \bigr ] {\mathbb {E}}_{N,\infty ,\kappa }\bigl [ f_1 ( \hat{\sigma }') \bigr ] \Bigr | \\  &\qquad \le 2\Vert f_0\Vert _\infty \Vert f_1 \Vert _\infty \mu _{N,\infty ,\kappa } \times \mu _{N,\infty ,\kappa } \bigl ( E_1 \cap E_{E_0,{\hat{\sigma }},\hat{\sigma }'} \ne \emptyset \bigr ) \\  &\qquad \le 2\Vert f_0\Vert _\infty \Vert f_1 \Vert _\infty \, \sum _{e \in E_1} \mu _{N,\infty ,\kappa } \times \mu _{N,\infty ,\kappa } \bigl ( e \in E_{E_0,{\hat{\sigma }},{\hat{\sigma }}'} \ne \emptyset \bigr ). \end{aligned} \end{aligned}$$

To obtain the desired conclusion, we note that by Lemma 5.9, we have $E_{E_0,{\hat{\sigma }},\hat{\sigma }'} = E_{E_0,\sigma ,\sigma '}.$ This concludes the proof. $\square$

### A coupling between the Abelian Higgs model and the ${\mathbb {Z}}_n$-model

In this section, we recall the coupling between $\mu _{N,\beta ,\kappa }$ and $\mu _{N,\infty ,\kappa }$ introduced in [18].

#### Definition 5.18

. For $\beta ,\kappa \ge 0,$ $\sigma \in \Omega ^1(B_N,G),$ and $\sigma ' \in \Omega ^1_0(B_N,G),$ let let

5.16 $$\begin{aligned} E_{\sigma , \sigma '} \, {:}{=}\, E_{\emptyset ,\sigma , \sigma '}= {\mathcal {C}}_{{\mathcal {G}}(\sigma , \sigma ')}\bigl ( \{ e \in {{\,\textrm{supp}\,}}\sigma :d \sigma |_{\pm {{\,\textrm{supp}\,}}{\hat{\partial }} e} \ne 0 \} \bigr ). \end{aligned}$$

and define

$$\begin{aligned} \mu _{N, (\beta ,\kappa ),(\infty ,\kappa )}(\sigma , \sigma ')&\, {:}{=}\, \mu _{N, \beta ,\kappa } \times \mu _{N,\infty , \kappa }\bigl (\bigl \{(\hat{\sigma }, \hat{\sigma }')\in \Omega ^1(B_N,G) \times \Omega ^1_0(B_N,G) :\\ \sigma \,&= \, \hat{\sigma }|_{E_{{\hat{\sigma }},\hat{\sigma }'}} + {\hat{\sigma }}'|_{C_1(B_N) \smallsetminus E_{\hat{\sigma },{\hat{\sigma }}'}} \text { and } \sigma '=\hat{\sigma }' \bigr \} \bigr ). \end{aligned}$$

We let ${\mathbb {E}}_{N,(\beta ,\kappa ),(\infty ,\kappa )}$ denote the corresponding expectation.

**Fig. 6:**
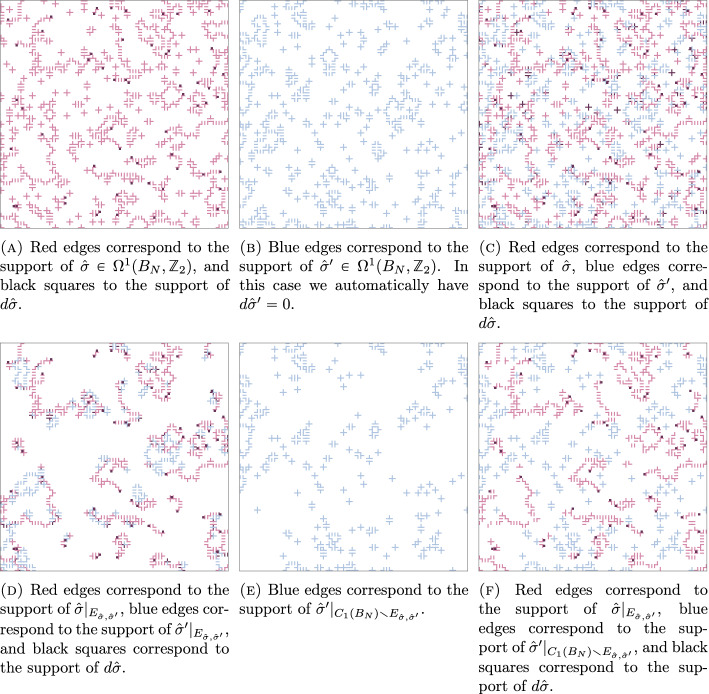
Illustration of the coupling $(\sigma , \sigma ') \sim \mu _{N,(\beta ,\kappa ),(\infty ,\kappa )}$ defined in Definition 5.18 (simulated on a 2-dimensional lattice, with $G = {\mathbb {Z}}_2$)

#### Remark 5.19

If [18], the measure $\mu _{N,(\beta ,\kappa ),(\infty ,\kappa )}$ in Definition 5.18 above was defined slightly differently, but using the Proposition 5.20 below, one easily shows that they are equivalent.

The next result shows that this is indeed a coupling.

#### Proposition 5.20

Let $\beta ,\kappa \ge 0$. Then $\mu _{N,(\beta ,\kappa ),{(\infty ,\kappa })}$ is a coupling of $\mu _{N,\beta ,\kappa }$ and $\mu _{N,\infty ,\kappa }$.

#### Proof

It is immediate from the definition that if $(\sigma ,\sigma ') \sim \mu _{N,(\beta ,\kappa ),(\infty ,\kappa )}$, then $\sigma ' \sim \mu _{N,\infty ,\kappa }$, and it is hence sufficient to show that $\sigma \sim \mu _{N,\beta ,\kappa }$. This is exactly equivalent to, for each $\sigma \in \Omega ^1(B_N,G)$, showing that

$$\begin{aligned}  &   \mu _{N,\beta ,\kappa }\times \mu _{N,\infty ,\kappa } \bigl ( \bigl \{ ({\hat{\sigma }}, {\hat{\sigma }}') \in \Omega ^1(B_N) \times \Omega ^1_0(B_N) :{\hat{\sigma }}|_{E_{ {\hat{\sigma }}, {\hat{\sigma }}'}} + \hat{\sigma }'|_{C_1(B_N)\smallsetminus E_{ {\hat{\sigma }}, {\hat{\sigma }}'}} = \sigma \bigr \} \bigr ) \\  &   \quad = \mu _{N,\beta ,\kappa }( \sigma ). \end{aligned}$$

or, equivalently, that

5.17 $$\begin{aligned}  &   \sum _{\begin{array}{c} {\hat{\sigma }} \in \Omega ^1(B_N,G),\\ {\hat{\sigma }}' \in \Omega ^1_0(B_N,G) \end{array}} \varphi _{\beta ,\kappa }({\hat{\sigma }}) \varphi _{\kappa }({\hat{\sigma }}') \cdot \mathbb {1} \bigl ( \hat{\sigma }|_{E_{{\hat{\sigma }},{\hat{\sigma }}'}} + \hat{\sigma }'|_{C_1(B_N)\smallsetminus E_{{\hat{\sigma }},{\hat{\sigma }}'}} \nonumber \\    &   \quad = \sigma \bigr ) = \varphi _{\beta ,\kappa }( \sigma ) \sum _{ \sigma ' \in \Omega ^1_0(B_N,G)} \varphi _{\kappa } ( \sigma '). \end{aligned}$$

We now show that (5.17) holds. To this end, fix some $\sigma \in \Omega ^1(B_N,G)$.

By Lemma 5.11, applied with $\beta _1= \beta$ and $\beta _2=\infty$, we have

5.18 $$\begin{aligned}&\sum _{\begin{array}{c} {\hat{\sigma }} \in \Omega ^1(B_N,G),\\ {\hat{\sigma }}' \in \Omega ^1_0(B_N,G) \end{array}} \varphi _{\beta ,\kappa }(\sigma ) \varphi _{\kappa }(\sigma ')\mathbb {1} \bigl ( {\hat{\sigma }}|_{E_{\hat{\sigma },{\hat{\sigma }}'}} + {\hat{\sigma }}'|_{C_1(B_N)\smallsetminus E_{ {\hat{\sigma }}, {\hat{\sigma }}'}} = \sigma \bigr ) \nonumber \\  &\qquad = \varphi _{\kappa }( \sigma ) \sum _{\sigma ' \in \Omega ^1(B_N,G)} \varphi _{\beta ,\kappa }(\sigma ') \sum _{\begin{array}{c} {\hat{\sigma }} \in \Omega ^1(B_N,G),\\ {\hat{\sigma }}' \in \Omega ^1_0(B_N,G) \end{array}} \mathbb {1}\bigl ( {\hat{\sigma }} = \sigma |_{E_{ \sigma , \sigma '}}+ \sigma '|_{C_1(B_N)\smallsetminus E_{ \sigma , \sigma '}} \bigr ) \nonumber \\&\hspace{22em}\cdot \mathbb {1}\bigl ( {\hat{\sigma }}' = \sigma ' |_{E_{ \sigma , \sigma '}} + \sigma |_{C_1(B_N)\smallsetminus E_{ \sigma , \sigma '}} \bigr ) \nonumber \\  &\qquad = \varphi _{\kappa }( \sigma ) \sum _{\sigma ' \in \Omega ^1(B_N,G)} \varphi _{\beta ,\kappa }(\sigma ') \mathbb {1}\bigl ( \sigma ' |_{E_{ \sigma , \sigma '}} + \sigma |_{C_1(B_N)\smallsetminus E_{ \sigma , \sigma '}} \in \Omega _0^1(B_N,G) \bigr ) \nonumber \\  &\qquad = \varphi _{\kappa }( \sigma ) \sum _{\sigma ' \in \Omega ^1(B_N,G)} \varphi _{\beta ,\kappa }(\sigma ') \mathbb {1}\Bigl ( d\bigl ( \sigma ' |_{E_{ \sigma , \sigma '}} + \sigma |_{C_1(B_N)\smallsetminus E_{ \sigma , \sigma '}}\bigr ) =0 \Bigr ). \end{aligned}$$

By Lemma 5.8, we have

$$\begin{aligned} d\bigl ( \sigma ' |_{E_{ \sigma , \sigma '}} + \sigma |_{C_1(B_N)\smallsetminus E_{ \sigma , \sigma '}} \bigr )  &   = d\bigl ( \sigma ' |_{E_{ \sigma , \sigma '}} \bigr )+ d\bigl (\sigma |_{C_1(B_N)\smallsetminus E_{ \sigma , \sigma '}} \bigr ) = d\bigl ( \sigma ' |_{E_{ \sigma , \sigma '}} \bigr ) +0\nonumber \\  &   = d\sigma ', \end{aligned}$$

and hence

5.19 $$\begin{aligned} \begin{aligned}&\varphi _{\kappa }( \sigma ) \sum _{\sigma ' \in \Omega ^1(B_N,G)} \varphi _{\beta ,\kappa }(\sigma ') \mathbb {1}\Bigl ( d\bigl ( \sigma ' |_{E_{ \sigma , \sigma '}} + \sigma |_{C_1(B_N)\smallsetminus E_{ \sigma , \sigma '}}\bigr ) =0 \Bigr ) \\  &\qquad = \varphi _{\kappa }( \sigma ) \sum _{\sigma ' \in \Omega ^1(B_N,G)} \varphi _{\beta ,\kappa }(\sigma ') \mathbb {1}( d\sigma ' =0 ) = \varphi _{\kappa }( \sigma ) \sum _{\sigma ' \in \Omega ^1_0(B_N,G)} \varphi _{\beta ,\kappa }(\sigma '). \end{aligned}\nonumber \\ \end{aligned}$$

Combining (5.18) and (5.19), we obtain (5.17) as desired. This concludes the proof. $\square$

#### Lemma 5.21

Let $\beta ,\kappa \ge 0,$ let $E_0 \subseteq C_1(B_N),$ and let $(\sigma ,\sigma ')\in \Omega ^1(B_N,G) \times \Omega ^1_0(B_N,G)$ be such that $\mu _{N,(\beta ,\kappa ),(\infty ,\kappa )}(\sigma , \sigma ') \ne 0$. Then $\sigma (e) = \sigma '(e)$ for all $e \in C_1(B_N) \smallsetminus E_{\sigma ,\sigma '}$.

#### Proof

Since $\mu _{N,(\beta ,\kappa ),(\infty ,\kappa )}(\sigma , \sigma ') \ne 0$, by definition, there is $({\hat{\sigma }}, {\hat{\sigma }}') \in \Omega ^1(B_N,G) \times \Omega ^1_0(B_N,G)$ such that $\sigma = \hat{\sigma }|_{E_{{\hat{\sigma }},{\hat{\sigma }}'}} + \hat{\sigma }'|_{C_1(B_N) \smallsetminus E_{\hat{\sigma }, \hat{\sigma }'}}$ and $\sigma ' = {\hat{\sigma }}'$. Using Lemma 5.9, we immediately obtain the desired conclusion. $\square$

## Distribution of Vortices and Edge Configurations

In this section, we use the edge graph defined in Sect. 5.1 to give upper bounds on several useful events. Throughout this section, constants $K_1,K_2,\dots , K_{15}$ will be introduced. We use distinct names for these to make it possible to find explicit upper bounds, but stress that under the assumptions of the main results these are all bounded from above, and will thus not affect the decay rate of the upper bounds obtained throughout this section.

For $E_0 \subseteq C_1(B_N)$ and $e \in C_1(B_N)$, we define

$$\begin{aligned} \begin{aligned} {{\,\textrm{dist}\,}}_1 (e,E_0)\,&{:}{=}\, \frac{1}{2}\min \Bigl \{ \bigl |{\mathcal {C}}_{{\mathcal {G}}_{{\hat{\sigma }},{\hat{\sigma }}'}}(e) \bigr | :\sigma ,\sigma ' \in \Omega ^1(B_N,G),\, {\mathcal {C}}_{{\mathcal {G}}_{{\hat{\sigma }},{\hat{\sigma }}'}}(e)\cap E_0 \ne \emptyset \Bigr \} \\  &= \, \frac{1}{2}\min \Bigl \{ \bigl |{\mathcal {C}}_{{\mathcal {G}}_{\hat{\sigma }}}(e) \bigr | :\sigma \in \Omega ^1(B_N,G),\, {\mathcal {C}}_{{\mathcal {G}}_{{\hat{\sigma }}}}(e)\cap E_0 \ne \emptyset \Bigr \},\quad e \in C_1(B_N), \end{aligned} \end{aligned}$$

and

$$\begin{aligned} {{\,\textrm{dist}\,}}_0 (e,E_0)  &   {:}{=}\, \frac{1}{2}\min \Bigl \{\bigl |{\mathcal {C}}_{{\mathcal {G}}_{{\hat{\sigma }},{\hat{\sigma }}'}}(e) \bigr | :\sigma ,\sigma ' \in \Omega ^1_0(B_N,G),\, {\mathcal {C}}_{{\mathcal {G}}_{{\hat{\sigma }},{\hat{\sigma }}'}}(e)\cap E_0 \ne \emptyset \Bigr \},\\  &   \quad e \in C_1(B_N). \end{aligned}$$

Note that, by Lemma 2.9, if $e \notin E,$ then ${{\,\textrm{dist}\,}}_0(e,E) \ge 8.$ We extend this definition to sets ${E \subseteq C_1(B_N)}$ by letting ${{\,\textrm{dist}\,}}_1(E,E_0) \, {:}{=}\, \min _{e \in E} {{\,\textrm{dist}\,}}_1(e,E_0)$ and ${{\,\textrm{dist}\,}}_0(E,E_0) \, {:}{=}\, \min _{e \in E} {{\,\textrm{dist}\,}}_0(e,E_0).$

In this section, we will state and prove the following three propositions.

### Proposition 6.1

Let $\beta ,\kappa _1,\kappa _2 \in [0,\infty ]$ be such that $18^2\bigl ( \alpha _0(\kappa _1) + \alpha _0(\kappa _2) + \alpha _0(\kappa _1)\alpha _0(\kappa _2)\bigr ) <1$, let $e \in C_1(B_N)$ be such that ${{\,\textrm{dist}\,}}_0\bigl (e,\partial C_1(B_N)\bigr ) \ge 8,$ and let $M \ge 1$ and $M' \ge 0.$

Then6.1where6.2

### Remark 6.2

If $\kappa _1 = \kappa _2 = :\kappa$, then the assumption on $\kappa _1$ and $\kappa _2$ in Proposition (6.1) is equivalent to [A].

### Proposition 6.3

Let $\beta ,\kappa \ge 0$ be such that [A] holds, and assume that $p \in C_2(B_N)$ is such that ${{\,\textrm{dist}\,}}_0({{\,\textrm{supp}\,}}\partial e,\partial C_1(B_N))) \ge 8.$ Then6.3where

6.4 $$\begin{aligned} K_2 \, {:}{=}\, \, 4 \bigl ( 18^{2} + 18 \alpha _0(\kappa ) \bigl (1-18^2 \alpha _0(\kappa )\bigr )^{-1} \bigr ) \frac{\alpha _1(\beta )^6}{\alpha _0(\beta )^6}. \end{aligned}$$

### Proposition 6.4

Let $\kappa \ge 0$ be such that [A] holds, let $E_0 \subseteq C_1(B_N)$ be non-empty, and let $e \in C_1(B_N)$ be such that ${{\,\textrm{dist}\,}}_0(e,\partial C_1(B_N))) \ge 8.$

Then

6.5 $$\begin{aligned}  &   \mu ^{E_0}_{N,(\infty ,\kappa ),(\infty ,\kappa )} \bigl ( \bigr \{ (\sigma ,\sigma ') \in \Omega ^1_0(B_N,G) \times \Omega ^1_0(B_N,G) :e \in E_{E_0,\sigma , \sigma '} \bigr \} \bigr )\nonumber \\    &   \le K_3 \Bigl ( K_4 \alpha _0(\kappa ) \Bigr )^{{{\,\textrm{dist}\,}}_0(e,E_0)} \end{aligned}$$

where

6.6 $$\begin{aligned} K_3 \, {:}{=}\, \, 18^{-3}(1 - 18^2 \bigl (2 + \alpha _0(\kappa ) \bigr )\alpha _0(\kappa ))^{-1}, \quad \text {and} \quad K_4 \, {:}{=}\, 18^{2} \bigl (2 + \alpha _0(\kappa ) \bigr ). \end{aligned}$$

### Proposition 6.5

Let $\beta ,\kappa \ge 0$ be such that [A] hold, let $e \in C_1(B_N)$ be such that for all $p \in {\hat{\partial }} e$ we have ${{{\,\textrm{dist}\,}}_0({{\,\textrm{supp}\,}}\partial p,\partial C_1(B_N))\ge 8},$ and let $E_0 \, {:}{=}\, \{ e' \in C_1(B_N) :\hat{\partial }e' \cap {\hat{\partial }} e \ne \emptyset \}.$ Thenwhere

6.7 $$\begin{aligned} K_5 \, {:}{=}\, \, (18^{-3} + 18^{-1}) (1-18^2 \alpha _0(\kappa ))^{-1}. \end{aligned}$$

Before we give proofs of the above propositions, we introduce some additional notation and prove two useful lemmas. To this end, we first define a graph $\bar{{\mathcal {G}}}$ as follows. Fix some $g \in G\smallsetminus \{ 0 \}$ and define ${\bar{\sigma }} \in \Omega ^1(B_N,G)$ by letting $\sigma (e) = g$ for all $e \in C_1(B_N)^+$. Let $\bar{{\mathcal {G}}} \, {:}{=}\, {\mathcal {G}}(\bar{\sigma },0)$ and note that $\bar{{\mathcal {G}}}$ does not depend on the choice of *g*. Note also that if $\sigma ,\sigma ' \in \Omega ^1(B_N,G)$, then ${\mathcal {G}}(\sigma , \sigma ')$ is a subgraph of $\bar{{\mathcal {G}}}$.

### Lemma 6.6

[See also Lemma 7.15 and Lemma 7.16 in [18]] Let $e \in C_1^+(B_N)$, and let $m \ge 1.$ Then

### Proof

Since the case $m = 1$ is trivial, we can assume that $m \ge 2.$

Fix some set $E \subseteq C_1^+(B_N)$ such that $e \in E,$ $|E| = m,$ and $\bar{{\mathcal {G}}}|_{E}$ is connected.

Since the graph $\bar{{\mathcal {G}}}|_{E}$ is connected, it has a spanning tree. Let ${\mathcal {T}}$ be such a spanning tree. By definition, ${\mathcal {T}}$ must contain exactly $m-1$ edges. Since any spanning tree is connected, ${\mathcal {T}}$ must have a spanning walk which uses each edge in ${\mathcal {T}}$ exactly twice, and starts and ends at the same vertex. This walk must have length $2(m-1) = 2m-2$. By removing one of the edges adjacent to the vertex *e*, we obtain a spanning walk of $\bar{{\mathcal {G}}}|_{E}$ which has length $2\,m-3$, starts at the vertex *e* and visits every vertex of $\bar{{\mathcal {G}}}|_{E}$ at least once.

Since for each $e' \in E_N$, we have $\bigl |\{ e'' \in C_1(B_N)\smallsetminus \{ e' \} :{\hat{\partial }} e'' \cap \hat{\partial }e' \ne \emptyset \}\bigr |= 6 \cdot 3 = 18,$ there can exists at most $18^{2m-3}$ walks in $\bar{{\mathcal {G}}}$ which starts at *e* and has length $2m-3,$ and hence the desired conclusion follows. $\square$

### Lemma 6.7

Let $\kappa _1,\kappa _2 \ge 0$, and let $E \subseteq C_1^+(B_N).$ Then6.8

### Proof

If $\hat{{\hat{\sigma }}} \in \Omega ^1(B_N,G)$ and $e' \notin {{\,\textrm{supp}\,}}\hat{{\hat{\sigma }}}$, then, for any $\kappa \ge 0$, we have $\varphi _\kappa \bigl (\hat{{\hat{\sigma }}}(e')\bigr ) = \varphi _\kappa (0)=1$. Also, if $\hat{{\hat{\sigma }}}, \hat{{\hat{\sigma }}}' \in \Omega ^1(B_N,G)$ and $e' \in ({{\,\textrm{supp}\,}}\hat{{\hat{\sigma }}} \cup {{\,\textrm{supp}\,}}\hat{{\hat{\sigma }}}')^+ = E$, then either $\hat{{\hat{\sigma }}}(e') \ne 0$ and $\hat{{\hat{\sigma }}}'(e') = 0$, $\hat{{\hat{\sigma }}}(e')=0$ and $\hat{{\hat{\sigma }}}'(e') \ne 0$, or $\hat{{\hat{\sigma }}}(e'),\hat{{\hat{\sigma }}}'(e') \ne 0$.

Combining these observations, we find that

$$\begin{aligned} \begin{aligned}&\sum _{\begin{array}{c} \hat{{\hat{\sigma }}} \in \Omega ^1(B_N,G),\, \hat{{\hat{\sigma }}}' \in \Omega ^1(B_N,G) :\\ ({{\,\textrm{supp}\,}}\hat{{\hat{\sigma }}} \cup {{\,\textrm{supp}\,}}\hat{{\hat{\sigma }}}')^+ = E \end{array}} \prod _{e' \in C_1(B_N)^+} \varphi _{\kappa _1}\bigl (\hat{{\hat{\sigma }}}(e')\bigr )^2 \prod _{e'' \in C_1(B_N)^+} \varphi _{\kappa _2}\bigl (\hat{{\hat{\sigma }}}'(e'')\bigr ) \\  &\quad \le \prod _{e' \in E} \biggl \{ \varphi _{\kappa _1}(0)^2 \bigg (\sum _{\hat{{\hat{\sigma }}}'(e') \in G \smallsetminus \{0\}} \varphi _{\kappa _2}(\hat{{\hat{\sigma }}}'(e'))^2\bigg ) + \biggl (\sum _{\hat{{\hat{\sigma }}}(e') \in G \smallsetminus \{0\}} \varphi _{\kappa _1}\bigl (\hat{{\hat{\sigma }}}(e')\bigr )^2 \biggr ) \varphi _{\kappa _2}(0)^2\\&\qquad +\bigg (\sum _{\hat{{\hat{\sigma }}}(e') \in G \smallsetminus \{0\}} \varphi _{\kappa _1}\bigl (\hat{{\hat{\sigma }}}(e')\bigr )^2 \bigg ) \bigg (\sum _{\hat{{\hat{\sigma }}}'(e') \in G \smallsetminus \{0\}} \varphi _{\kappa _2}\bigl (\hat{{\hat{\sigma }}}'(e')\bigr )^2\bigg ) \biggr \} \\&\quad = \prod _{e' \in E} \bigl (\alpha _0(\kappa _1) + \alpha _0(\kappa _2) + \alpha _0(\kappa _1)\alpha _0(\kappa _2)\bigr ) = \bigl (\alpha _0(\kappa _1) + \alpha _0(\kappa _2) + \alpha _0(\kappa _1)\alpha _0(\kappa _2)\bigr )^{|E|}. \end{aligned} \end{aligned}$$

This concludes the proof. $\square$

### Proof of Proposition 6.1

Since ${\mathcal {C}}_{{\mathcal {G}}({\hat{\sigma }},{\hat{\sigma }}')}(e)$ is symmetric, induces a connected subgraph in $\bar{{\mathcal {G}}}$, and contains *e* if it non-empty, we have6.9Given $({\hat{\sigma }},{\hat{\sigma }}') \!\in \! \Omega ^1(B_N,G) \!\times \! \Omega ^1_0(B_N,G)$, if we let $\hat{{\hat{\sigma }}} \, {:}{=}\, \hat{\sigma }|_{{\mathcal {C}}_{{\mathcal {G}}({\hat{\sigma }},{\hat{\sigma }}')}(e)}$ and $\hat{{\hat{\sigma }}}' \, {:}{=}\, \hat{\sigma }'|_{{\mathcal {C}}_{{\mathcal {G}}({\hat{\sigma }},{\hat{\sigma }}')}(e)}$, then the following statements hold.

1. By Lemma 5.8, we have $\hat{{\hat{\sigma }}}' \in \Omega ^1_0(B_N,G)$.
2. If $\bigl | {{\,\textrm{supp}\,}}d(\hat{\sigma }|_{{\mathcal {C}}_{{\mathcal {G}}({\hat{\sigma }}, \hat{\sigma }')}(e)})\bigr | \ge 2\,M'$, then, by definition, $\bigl | {{\,\textrm{supp}\,}}d\hat{{\hat{\sigma }}} \bigr | \ge 2\,M'.$
3. If $|{\mathcal {C}}_{{\mathcal {G}}({\hat{\sigma }},{\hat{\sigma }}')}(e) |\ge 2$, then we have $e \in {{\,\textrm{supp}\,}}{\hat{\sigma }} \cup {{\,\textrm{supp}\,}}{\hat{\sigma }}',$ and thus $({\mathcal {C}}_{{\mathcal {G}}({\hat{\sigma }},{\hat{\sigma }}')}(e))^+ = ({{\,\textrm{supp}\,}}\hat{{\hat{\sigma }}} \cup {{\,\textrm{supp}\,}}\hat{{\hat{\sigma }}}')^+$. Consequently, $({\mathcal {C}}_{{\mathcal {G}}({\hat{\sigma }},\hat{\sigma }')}(e))^+ = E$ if and only if $({{\,\textrm{supp}\,}}\hat{{\hat{\sigma }}} \cup {{\,\textrm{supp}\,}}\hat{{\hat{\sigma }}}')^+ = E.$

As a consequence,  (6.9) can be bounded from above byFor any $\hat{{\hat{\sigma }}}$, and $\hat{{\hat{\sigma }}}'$ as in the sum above, by Lemma 5.2, we have

$$\begin{aligned} \begin{aligned}&\mu _{N,\beta ,\kappa _1} \times \mu _{N,\infty ,\kappa _2} \bigl ( \{ ({\hat{\sigma }},{\hat{\sigma }}') \in \Omega ^1(B_N,G) \times \Omega ^1_0(B_N,G) :{\hat{\sigma }}|_{{\mathcal {C}}_{{\mathcal {G}}(\hat{\sigma }, {\hat{\sigma }}')}(e)} \\  &\quad = \hat{{\hat{\sigma }}} \text { and } \hat{\sigma }'|_{{\mathcal {C}}_{{\mathcal {G}}({\hat{\sigma }}, {\hat{\sigma }}'}(e)} = \hat{{\hat{\sigma }}}'\} \bigr ) \\  &\qquad {\le } \mu _{N,\beta ,\kappa _1} {\times } \mu _{N,\infty ,\kappa _2} \bigl ( \{ ({\hat{\sigma }},{\hat{\sigma }}') \in \Omega ^1(B_N,G) {\times } \Omega ^1_0(B_N,G) :\hat{{\hat{\sigma }}} \le {\hat{\sigma }} \text { and } \hat{{\hat{\sigma }}}' \le {\hat{\sigma }}' \} \bigr ) \\  &\qquad = \mu _{N,\beta ,\kappa _1} \bigl ( \{ {\hat{\sigma }} \in \Omega ^1(B_N,G) :\hat{{\hat{\sigma }}} \le {\hat{\sigma }} \} \bigr ) \mu _{N,\infty ,\kappa _2} \bigl ( \{ {\hat{\sigma }}' \in \Omega ^1_0(B_N,G) :\hat{{\hat{\sigma }}}' \le {\hat{\sigma }}' \} \bigr )\\&\qquad \le \varphi _{\beta ,\kappa _1}( \hat{{\hat{\sigma }}}) \varphi _{\infty ,\kappa _2} (\hat{{\hat{\sigma }}}'), \end{aligned} \end{aligned}$$

where the last inequality follows by applying Proposition 4.2 twice.

Taken together, the above equations thus show that6.10where

$$\begin{aligned} J_{\beta , \kappa _1,\kappa _2}(E) \, {:}{=}\, \sum _{\begin{array}{c} \hat{{\hat{\sigma }}} \in \Omega ^1(B_N,G),\, \hat{{\hat{\sigma }}}' \in \Omega ^1_0(B_N,G) :\\ \begin{array}{c} ({{\,\textrm{supp}\,}}\hat{{\hat{\sigma }}} \cup {{\,\textrm{supp}\,}}\hat{{\hat{\sigma }}}')^+ = E,\\ \bigl | {{\,\textrm{supp}\,}}d \sigma \bigr | \ge 2M' \end{array} \end{array}} \varphi _{\beta ,\kappa _1} ( \hat{{\hat{\sigma }}} )\, \varphi _{\infty ,\kappa _2} ( \hat{{\hat{\sigma }}}' ). \end{aligned}$$

Fix some set $E \subseteq C_1^+(B_N).$ Then

$$\begin{aligned} \begin{aligned}&J_{\beta , \kappa _1,\kappa _2}(E) = \sum _{\begin{array}{c} \hat{{\hat{\sigma }}} \in \Omega ^1(B_N,G),\, \hat{{\hat{\sigma }}}' \in \Omega ^1_0(B_N,G) :\\ \begin{array}{c} ({{\,\textrm{supp}\,}}\hat{{\hat{\sigma }}} \cup {{\,\textrm{supp}\,}}\hat{{\hat{\sigma }}}')^+ = E,\\ |{{\,\textrm{supp}\,}}d \hat{{\hat{\sigma }}} | \ge 2M' \end{array} \end{array}} \varphi _{\kappa _1}\bigl (\hat{{\hat{\sigma }}}\bigr ) \varphi _{\kappa _2}\bigl (\hat{{\hat{\sigma }}}'\bigr ) \prod _{p \in C_2(B_N)}\varphi _\beta \bigl (d\hat{{\hat{\sigma }}}(p) \bigr ). \end{aligned} \end{aligned}$$

Now recall that for any $r \ge 0$ and $g \in G$, we have $\varphi _r(0) = 1$ and $\varphi _r(g) = e^{r \Re (\rho (g) - 1)} \in (0,1]$. If $g \ne 0$, then $\varphi _r(g) < 1$ and hence $\varphi _\beta (g)^2 \le \alpha _1(\beta ) < 1$.

If $\hat{{\hat{\sigma }}},$ $\hat{{\hat{\sigma }}}'$ and *E* are as above, then we must be in one of the following three cases.

1. If $$|({{\,\textrm{supp}\,}}d\hat{{\hat{\sigma }}})^+| \ge 6$$, then
  $$\begin{aligned} \prod _{p \in C_2(B_N)}\varphi _\beta \bigl (d\hat{{\hat{\sigma }}}(p) \bigr ) = \prod _{p \in C_2(B_N,G)^+}\varphi _\beta \bigl ((d\hat{{\hat{\sigma }}})_p \bigr )^2 \le \alpha _1(\beta )^{\max (M',6)}. \end{aligned}$$
2. If $|({{\,\textrm{supp}\,}}d\hat{{\hat{\sigma }}})^+| \in \{ 1,2,3,4,5 \}$, by Lemma 2.8, $\hat{{\hat{\sigma }}}$ must support a vortex with support at the boundary of $B_N$, and hence we must have $|E| \ge {{\,\textrm{dist}\,}}_1(e,\partial C_1(B_N)).$ At the same time, by definition, we also have $\prod _{p \in C_1(B_N,G)}\varphi _\beta \bigl (d\hat{{\hat{\sigma }}}(p) \bigr ) \le \alpha _1(\beta ).$
3. If $|({{\,\textrm{supp}\,}}d\hat{{\hat{\sigma }}})^+| = 0,$ then $\hat{{\hat{\sigma }}} \in \Omega ^1_0(B_N,G).$ Since $|{{\,\textrm{supp}\,}}(\hat{{\hat{\sigma }}})^+ \cup {{\,\textrm{supp}\,}}(\hat{{\hat{\sigma }}}')^+| > 0$, it follows from Lemma 2.9 that $|E| \ge \min (M,8,{{\,\textrm{dist}\,}}_0(e, \partial C_1(B_N))).$ Moreover, we have $\prod _{p \in C_1(B_N,G)}\varphi _\beta \bigl (d\hat{{\hat{\sigma }}}(p) \bigr ) = 1.$

Consequently, we have

$$\begin{aligned} \begin{aligned} J_{\beta , \kappa _1,\kappa _2}(E)&\le \bigl ( \alpha _1(\beta )^{\max (6,M')} + \mathbb {1}_{M'\in \{ 1,2,3,4,5 \},\,|E| \ge {{\,\textrm{dist}\,}}_1(e,\partial C_1(B_N))} \cdot \alpha _1(\beta )\\&\quad + \, \mathbb {1}_{M'=0,\, |E| \ge \max (M,\min (8,{{\,\textrm{dist}\,}}_0(e, \partial C_1(B_N))))} \bigr )\\&\quad \sum _{\begin{array}{c} \hat{{\hat{\sigma }}} \in \Omega ^1(B_N,G),\, \hat{{\hat{\sigma }}}' \in \Omega ^1_0(B_N,G) :\\ \begin{array}{c} ({{\,\textrm{supp}\,}}\hat{{\hat{\sigma }}} \cup {{\,\textrm{supp}\,}}\hat{{\hat{\sigma }}}')^+ = E, \\ |{{\,\textrm{supp}\,}}d\hat{{\hat{\sigma }}}| \ge 2M' \end{array} \end{array}} \!\!\!\! \varphi _{\kappa _1}\bigl (\hat{{\hat{\sigma }}}\bigr ) \varphi _{\kappa _2}\bigl (\hat{{\hat{\sigma }}}'\bigr ). \end{aligned} \end{aligned}$$

By dropping the condition $\bigl |{{\,\textrm{supp}\,}}d\hat{{\hat{\sigma }}}\bigr | \ge 2\,M'$, and replacing the condition $\hat{{\hat{\sigma }}}' \in \Omega ^1_0(B_N,G)$ with the condition that $\hat{{\hat{\sigma }}}' \in \Omega ^1(B_N,G)$, we make the sum larger.

Hence

$$\begin{aligned} \begin{aligned}&\sum _{\begin{array}{c} \hat{{\hat{\sigma }}} \in \Omega ^1(B_N,G),\, \hat{{\hat{\sigma }}}' \in \Omega ^1_0(B_N,G) :\\ \begin{array}{c} ({{\,\textrm{supp}\,}}\hat{{\hat{\sigma }}} \cup {{\,\textrm{supp}\,}}\hat{{\hat{\sigma }}}')^+ = E, \\ |{{\,\textrm{supp}\,}}d\hat{{\hat{\sigma }}}| \ge 2M' \end{array} \end{array}} \varphi _{\kappa _1}\bigl (\hat{{\hat{\sigma }}}\bigr ) \varphi _{\kappa _2}\bigl (\hat{{\hat{\sigma }}}'\bigr ) \le \sum _{\begin{array}{c} \hat{{\hat{\sigma }}} \in \Omega ^1(B_N,G),\, \hat{{\hat{\sigma }}}' \in \Omega ^1(B_N,G) :\\ ({{\,\textrm{supp}\,}}\hat{{\hat{\sigma }}} \cup {{\,\textrm{supp}\,}}\hat{{\hat{\sigma }}}')^+ = E \end{array}} \varphi _{\kappa _1}\bigl (\hat{{\hat{\sigma }}}\bigr ) \varphi _{\kappa _2}\bigl (\hat{{\hat{\sigma }}}'\bigr ). \end{aligned} \end{aligned}$$

Using Lemma 6.7, we thus obtain6.11Combining (6.10) and (6.11) and applying Lemma 6.6, we now finally obtainComputing the above geometric sums, we obtain (6.3) as desired. $\square$

### Proof of Proposition 6.3

If ${\hat{\sigma }} \in \Omega ^1(B_N,G)$ satisfies $d {\hat{\sigma }}(p) \ne 0$, then there must exist $e \in \partial p$ such that $\sigma (e) \ne 0.$ For any such *e*,  we must have $|({\mathcal {C}}_{{\mathcal {G}}({\hat{\sigma }},0)}(e))^+| \ge 1$. Moreover, since $\sigma (e) \ne 0$, for any $e' \in \partial p$ such that $\sigma (e') \ne 0$, by definition, we have $e' \in {\mathcal {C}}_{{\mathcal {G}}({\hat{\sigma }},0)}(e).$ Consequently, we must have $d({\hat{\sigma }}|_{{\mathcal {C}}_{{\mathcal {G}}(\hat{\sigma },0)}(e)})(p) = d{\hat{\sigma }}(p) \ne 0.$ Using Lemma 2.8, it follows that $|({{\,\textrm{supp}\,}}d({\hat{\sigma }}|_{{\mathcal {C}}_{{\mathcal {G}}(\hat{\sigma },0)}(e)}))^+|\ge 6.$ Combining these observations with a union bound, it follows thatApplying Proposition 6.1 with $\kappa _1 = \kappa ,$ $\kappa _2 = \infty ,$ $M = 1,$ and $M' = 6,$ we obtain (6.3) as desired. $\square$

### Proof of Proposition 6.4

Recall first that by the definition of $\mu ^{E_0}_{N,(\infty ,\kappa ),(\infty ,\kappa )}$, using Lemma 5.9, we have

$$\begin{aligned} \begin{aligned}&\mu ^{E_0}_{N,(\infty ,\kappa ),(\infty ,\kappa )} \bigl ( \big \{ ( \sigma , \sigma ') \in \Omega ^1_0(B_N,G) \times \Omega ^1_0(B_N,G) :e \in E_{E_0,\sigma , \sigma '}\big \}\bigr ) \\  &\qquad = \mu _{N,\infty ,\kappa } \times \mu _{N,\infty ,\kappa } \bigl ( \big \{ ({\hat{\sigma }},{\hat{\sigma }}') \in \Omega ^1_0(B_N,G) \times \Omega ^1_0(B_N,G) :e \in E_{{\hat{\sigma }},{\hat{\sigma }}'} \big \}\bigr ). \end{aligned} \end{aligned}$$

Next, since ${\hat{\sigma }},{\hat{\sigma }}' \in \Omega ^1_0(B_N,G),$ we have $d{\hat{\sigma }} = d{\hat{\sigma }}' = 0.$ Consequently,

$$\begin{aligned} e \in E_{E_0,{\hat{\sigma }},{\hat{\sigma }}'} \Leftrightarrow e \in {\mathcal {C}}_{{\mathcal {G}}({\hat{\sigma }},{\hat{\sigma }}')}(E_0) \Leftrightarrow E_0 \cap {\mathcal {C}}_{{\mathcal {G}}({\hat{\sigma }},\hat{\sigma }')}(e) \ne \emptyset . \end{aligned}$$

Finally, note that if $E_0 \cap {\mathcal {C}}_{{\mathcal {G}}(\hat{\sigma },{\hat{\sigma }}')}(e) \ne \emptyset$, then, by definition, we must have $|({\mathcal {C}}_{{\mathcal {G}}({\hat{\sigma }},\hat{\sigma }')}(e))^+ | \ge {{\,\textrm{dist}\,}}_0(e,E_0).$

Combining these observations, it follows that

$$\begin{aligned} \begin{aligned}&\mu ^{E_0}_{N,(\infty ,\kappa ),(\infty ,\kappa )} \bigl ( \big \{ ( \sigma , \sigma ') \in \Omega ^1_0(B_N,G) \times \Omega ^1_0(B_N,G) :e \in E_{E_0,\sigma , \sigma '}\big \}\bigr ) \\&\qquad \le \mu _{N,\infty ,\kappa } \times \mu _{N,\infty ,\kappa } \bigl ( \big \{ ({\hat{\sigma }},{\hat{\sigma }}') \in \Omega ^1_0(B_N,G) \times \Omega ^1_0(B_N,G) :|{\mathcal {C}}_{{\mathcal {G}}({\hat{\sigma }},\hat{\sigma }')}(e) |\\&\qquad \ge {{\,\textrm{dist}\,}}_0(e,E_0) \big \}\bigr ). \end{aligned} \end{aligned}$$

Applying Proposition 6.1 with $\kappa _1 = \kappa _2 = \kappa ,$ $\beta = \infty ,$ $M = {{\,\textrm{dist}\,}}_0(e,E_0),$ and $M' = 0$, we obtain (6.5) as desired. $\square$

### Proof of Proposition 6.5

Without loss of generality, we can assume that $e \in C_1(B_N)^+$. To simplify notation, let

$$\begin{aligned} {\mathcal {E}} \, {:}{=}\, \bigl \{ \sigma \in \Omega ^1(B_N,G) :|{\mathcal {C}}_{{\mathcal {G}}(\sigma )}(E_0)| \ge 2M, \text { and }\bigl | {{\,\textrm{supp}\,}}d\bigl ( \sigma |_{ {\mathcal {C}}_{{\mathcal {G}}(\sigma )}(E_0)} \bigr ) \bigr | \ge 2M' \bigr \}. \end{aligned}$$

Now note that ${\mathcal {C}}_{{\mathcal {G}}({\hat{\sigma }},\hat{\sigma }')}(E_0)$ is symmetric, and that the set ${\mathcal {C}}_{{\mathcal {G}}({\hat{\sigma }},{\hat{\sigma }}')}(E_0) \cup \{ e,-e \}$ induces a connected set in $\bar{{\mathcal {G}}}$. Consequently, we haveGiven $\sigma \in \Omega ^1(B_N,G)$, if we let $\hat{{\hat{\sigma }}} \, {:}{=}\, \sigma |_{{\mathcal {C}}_{{\mathcal {G}}(\sigma )}(E_0)}$, then the following statements hold.

1. If $\bigl | {{\,\textrm{supp}\,}}d( \sigma |_{{\mathcal {C}}_{{\mathcal {G}}( \sigma , 0)}(E_0)})\bigr | \ge 2\,M'$, then, by definition, $\bigl | {{\,\textrm{supp}\,}}d\hat{{\hat{\sigma }}} \bigr | \ge 2\,M'.$
2. If $d( \sigma |_{{\mathcal {C}}_{{\mathcal {G}}( \sigma , 0)}(E_0)})\ne 0$, then $E_0 \cap {{\,\textrm{supp}\,}}\sigma \ne \emptyset$ and thus $({\mathcal {C}}_{{\mathcal {G}}(\sigma )}(E_0))^+ = ({{\,\textrm{supp}\,}}\hat{{\hat{\sigma }}} )^+$.

As a consequence, the expression in the previous equation is bounded from above by6.12For any $\hat{{\hat{\sigma }}}$ as in the sum above, by applying first Lemma 5.2, and then Proposition 4.2, we have

$$\begin{aligned} \begin{aligned}&\mu _{N,\beta ,\kappa } \bigl ( \{ {\hat{\sigma }} \in \Omega ^1(B_N,G) :{\hat{\sigma }}|_{{\mathcal {C}}_{{\mathcal {G}}({\hat{\sigma }}, 0)}(E_0)} = \hat{{\hat{\sigma }}} \} \bigr ) \le \mu _{N,\beta ,\kappa } \bigl ( \{ {\hat{\sigma }} \in \Omega ^1(B_N,G) :\hat{{\hat{\sigma }}} \le \hat{\sigma }\} \bigr ) \\&\quad \le \varphi _{\beta ,\kappa }( \hat{{\hat{\sigma }}}). \end{aligned} \end{aligned}$$

Taken together, the above equations show that6.13where

$$\begin{aligned} J_{\beta , \kappa }(E) \, {:}{=}\, \sum _{\begin{array}{c} \hat{{\hat{\sigma }}} \in \Omega ^1(B_N,G):\\ \begin{array}{c} ({{\,\textrm{supp}\,}}\hat{{\hat{\sigma }}})^+ = {{\,\textrm{supp}\,}}E,\\ |{{\,\textrm{supp}\,}}d \hat{{\hat{\sigma }}} |\ge 2M' \end{array} \end{array}} \varphi _{\beta ,\kappa } ( \hat{{\hat{\sigma }}} ). \end{aligned}$$

Now recall that

$$\begin{aligned} \begin{aligned}&\varphi _{\beta ,\kappa }( \hat{{\hat{\sigma }}}) = \prod _{e' \in C_1(B_N)} \varphi _\kappa \bigl (\hat{{\hat{\sigma }}}(e')\bigr ) \prod _{p \in C_2(B_N)}\varphi _\beta \bigl (d\hat{{\hat{\sigma }}}(p) \bigr ). \end{aligned} \end{aligned}$$

Also, recall that for any $r \ge 0$ and $g \in G$, we have $\varphi _r(0) = 1$ and $\varphi _r(g) = e^{r \Re (\rho (g) - 1)} \in (0,1]$. If $g \ne 0$, then $\varphi _r(g) < 1$ and hence $\varphi _\beta (g)^2 \le \alpha _1(\beta ) < 1$.

If $\hat{{\hat{\sigma }}}$ is as above, then $|{{\,\textrm{supp}\,}}d\hat{{\hat{\sigma }}}| \ge 2\,M'$, and hence

$$\begin{aligned} \prod _{p \in C_2(B_N,G)^+}\varphi _\beta \bigl ((d\hat{{\hat{\sigma }}})_p \bigr )^2 \le \alpha _1(\beta )^{M'}. \end{aligned}$$

Consequently, if *E* is as above, then

$$\begin{aligned} \begin{aligned}&J_{\beta , \kappa }(E) \le \alpha _1(\beta )^{M'} \sum _{\begin{array}{c} \hat{{\hat{\sigma }}} \in \Omega ^1(B_N,G) :\\ \begin{array}{c} ({{\,\textrm{supp}\,}}\hat{{\hat{\sigma }}})^+ = E, \\ |{{\,\textrm{supp}\,}}d\hat{{\hat{\sigma }}}| \ge 2M' \end{array} \end{array}} \prod _{e' \in C_1(B_N)^+} \varphi _\kappa \bigl (\hat{{\hat{\sigma }}}(e')\bigr )^2. \end{aligned} \end{aligned}$$

By dropping the condition $\bigl |{{\,\textrm{supp}\,}}d\hat{{\hat{\sigma }}}\bigr | \ge 2\,M'$ we make the sum larger. Hence

$$\begin{aligned} \begin{aligned}&J_{\beta , \kappa }(E) \le \alpha _1(\beta )^{M'} \sum _{\begin{array}{c} \hat{{\hat{\sigma }}} \in \Omega ^1(B_N,G) :\\ ({{\,\textrm{supp}\,}}\hat{{\hat{\sigma }}})^+ = {{\,\textrm{supp}\,}}W \end{array}} \prod _{e' \in C_1(B_N)^+} \varphi _\kappa \bigl (\hat{{\hat{\sigma }}}(e')\bigr )^2. \end{aligned} \end{aligned}$$

If $\hat{{\hat{\sigma }}} \in \Omega ^1(B_N,G)$ and $e' \notin {{\,\textrm{supp}\,}}\hat{{\hat{\sigma }}}$, then $\varphi _\kappa \bigl (\hat{{\hat{\sigma }}}(e')\bigr ) = \varphi _\kappa (0)=1$. Also, if $\hat{{\hat{\sigma }}} \in \Omega ^1(B_N,G)$ and $e' \in ({{\,\textrm{supp}\,}}\hat{{\hat{\sigma }}})^+$, then $\hat{{\hat{\sigma }}}(e') \ne 0.$

Using this observation, we obtain

6.14 $$\begin{aligned} \begin{aligned}&\sum _{\begin{array}{c} \hat{{\hat{\sigma }}} \in \Omega ^1(B_N,G) :\\ ({{\,\textrm{supp}\,}}\hat{{\hat{\sigma }}} ')^+ = E \end{array}} \prod _{e' \in C_1(B_N)^+} \varphi _\kappa \bigl (\hat{{\hat{\sigma }}}(e')\bigr )^2 \le \prod _{e' \in E } \sum _{\hat{{\hat{\sigma }}}'(e') \in G \smallsetminus \{0\}} \varphi _\kappa (\hat{{\hat{\sigma }}}'(e'))^2 = \prod _{e' \in E } \alpha _0(\kappa ) \\&\quad = \alpha _0(\kappa )^{|E|}, \end{aligned}\nonumber \\ \end{aligned}$$

We thus have

6.15 $$\begin{aligned}&J_{\beta , \kappa }(E) \le \alpha _1(\beta )^{M'} \alpha _0(\kappa ) ^{|E|}. \end{aligned}$$

Now note that, by Lemma 6.6, for any $m \ge M,$ we haveCombining this with (6.13) and (6.11), we thus find that

$$\begin{aligned} \mu _{N,\beta ,\kappa } ( {\mathcal {E}}) \le \sum _{m=M}^\infty (18^{2m-3} + 18^{2(m+1)-3}) \alpha _0(\kappa )^m \alpha _1(\beta )^{M'}. \end{aligned}$$

Computing the above geometric sum, we obtain (6.3). $\square$

## A First Version of Our Main Result

In this section, we present a first application of the coupling introduced in Sect. 5.4, by giving a first version of Theorem 10.1. This result provides an upper bound on $\langle L_\gamma (\sigma ,\phi ) \rangle$ which is good when the probability is small that there is a cluster in ${\mathcal {G}}({\hat{\sigma }},{\hat{\sigma }}')$ which both intersects ${{\,\textrm{supp}\,}}\gamma$ and supports a vortex. We later present a strengthening of this result in Proposition 10.18.

### Proposition 7.1

Let $\beta ,\kappa \ge 0$ be such that [A] holds, and let $\gamma$ be a path with finite support. Then

7.1 $$\begin{aligned} \Bigl | \bigl \langle L_\gamma (\sigma ,\phi ) \bigr \rangle _{\beta ,\kappa ,\infty } - \bigl \langle L_\gamma (\sigma ,\phi )\bigr \rangle _{\infty ,\kappa ,\infty } \Bigr | \le 2 K_4 \bigl ( 1 + K_3 K_4 \alpha _0(\kappa )\bigr )|{{\,\textrm{supp}\,}}\gamma |\alpha _0(\kappa ) \alpha _1(\beta )^{6},\nonumber \\ \end{aligned}$$

where $K_3$ and $K_4$ are defined by (6.6).

### Proof

Let $N \ge 1$ be large enough so that ${{\,\textrm{supp}\,}}\gamma \subseteq C_1(B_N)$ and ${{\,\textrm{dist}\,}}_0({{\,\textrm{supp}\,}}\gamma ,\partial C_1(B_N))) \ge 8.$

Then, by definition, we have

$$\begin{aligned} \begin{aligned} {\mathbb {E}}_{N,\beta ,\kappa } \bigl [L_\gamma (\sigma ) \bigr ]&= {\mathbb {E}}_{N,(\beta ,\kappa ),(\infty ,\kappa )} \bigl [ L_\gamma (\sigma ) \bigr ] = \mu _{N,\beta ,\kappa } \times \mu _{N,\infty ,\kappa } \bigl [ L_\gamma ({\hat{\sigma }}|_{E_{\hat{\sigma },{\hat{\sigma }}'}}\\&\quad \ + {\hat{\sigma }}'|_{C_1(B_N)\smallsetminus E_{{\hat{\sigma }},{\hat{\sigma }}'}}) \bigr ]. \end{aligned} \end{aligned}$$

On the event ${{\,\textrm{supp}\,}}\gamma \cap E_{{\hat{\sigma }},{\hat{\sigma }}'} = \emptyset ,$

we have

$$\begin{aligned} \begin{aligned}&L_\gamma ({\hat{\sigma }}|_{E_{{\hat{\sigma }},{\hat{\sigma }}'}} + \hat{\sigma }'|_{C_1(B_N)\smallsetminus E_{{\hat{\sigma }},{\hat{\sigma }}'}})&= L_\gamma ( 0 + {\hat{\sigma }}'|_{C_1(B_N)\smallsetminus E_{\hat{\sigma },{\hat{\sigma }}'}}) =L_\gamma ({\hat{\sigma }}'|_{E_{{\hat{\sigma }},\hat{\sigma }'}} + {\hat{\sigma }}'|_{C_1(B_N)\smallsetminus E_{\hat{\sigma },{\hat{\sigma }}'}}) \\  &\quad = L_\gamma ({\hat{\sigma }}'). \end{aligned} \end{aligned}$$

As a consequence,

$$\begin{aligned} \begin{aligned}&\mu _{N,\beta ,\kappa } \times \mu _{N,\infty ,\kappa } \bigl [ L_\gamma ({\hat{\sigma }}|_{E_{{\hat{\sigma }},{\hat{\sigma }}'}} + \hat{\sigma }'|_{C_1(B_N)\smallsetminus E_{{\hat{\sigma }},{\hat{\sigma }}'}}) \bigr ] \\  &\quad = \mu _{N,\beta ,\kappa } \times \mu _{N,\infty ,\kappa } \bigl [ L_\gamma ( {\hat{\sigma }}') \cdot \mathbb {1}_{{{\,\textrm{supp}\,}}\gamma \cap E_{\hat{\sigma },{\hat{\sigma }}'}= \emptyset }\bigr ] \\  &\qquad + \mu _{N,\beta ,\kappa } \times \mu _{N,\infty ,\kappa } \bigl [ L_\gamma ({\hat{\sigma }}|_{E_{{\hat{\sigma }},{\hat{\sigma }}'}} + \hat{\sigma }'|_{C_1(B_N)\smallsetminus E_{{\hat{\sigma }},{\hat{\sigma }}'}}) \cdot \mathbb {1}_{{{\,\textrm{supp}\,}}\gamma \cap E_{{\hat{\sigma }},\hat{\sigma }'}\ne \emptyset } \bigr ] \\  &\quad = \mu _{N,\beta ,\kappa } \times \mu _{N,\infty ,\kappa } \bigl [ L_\gamma ( {\hat{\sigma }}') \bigr ] - \mu _{N,\beta ,\kappa } \times \mu _{N,\infty ,\kappa } \bigl [ L_\gamma ( {\hat{\sigma }}') \cdot \mathbb {1}_{{{\,\textrm{supp}\,}}\gamma \cap E_{{\hat{\sigma }},{\hat{\sigma }}'}\ne \emptyset }\bigr ] \\  &\qquad + \mu _{N,\beta ,\kappa } \times \mu _{N,\infty ,\kappa } \bigl [ L_\gamma ({\hat{\sigma }}|_{E_{{\hat{\sigma }},{\hat{\sigma }}'}} + \hat{\sigma }'|_{C_1(B_N)\smallsetminus E_{{\hat{\sigma }},{\hat{\sigma }}'}}) \cdot \mathbb {1}_{{{\,\textrm{supp}\,}}\gamma \cap E_{{\hat{\sigma }},{\hat{\sigma }}'} \ne \emptyset } \bigr ] \end{aligned} \end{aligned}$$

Since $\rho$ is unitary and $L_\gamma (\sigma ) = \rho (\sigma (\gamma ))$ for any $\sigma \in \Omega ^1(B_N,G),$ it follows thatBy Lemma 5.10, if $e \in C_1(B_N)$, $\hat{\sigma }\in \Omega ^1(B_N,G)$ and ${\hat{\sigma }}' \in \Omega ^1_0(B_N,G)$, then $e \in E_{{\hat{\sigma }},{\hat{\sigma }}'}$ if and only if $d({\hat{\sigma }}|_{{\mathcal {C}}_{{\mathcal {G}}(\hat{\sigma },{\hat{\sigma }}')}(e)})\ne 0.$ On the other hand, if $d(\hat{\sigma }|_{{\mathcal {C}}_{{\mathcal {G}}({\hat{\sigma }},\hat{\sigma }')}(e)})\ne 0,$ then we must have $e \in {{\,\textrm{supp}\,}}\hat{\sigma }|_{{\mathcal {C}}_{{\mathcal {G}}({\hat{\sigma }},{\hat{\sigma }}')}(e)}$, implying in particular that $-e \in {\mathcal {C}}_{{\mathcal {G}}(\hat{\sigma },{\hat{\sigma }}')}(e)$, and hence $|{\mathcal {C}}_{{\mathcal {G}}({\hat{\sigma }},{\hat{\sigma }}')}(e)| \ge 2.$ Applying Proposition 6.1 with $M = M' = 1$ and $\kappa _1=\kappa _2 = \kappa ,$ we thus obtainCombining the above equations and letting $N \rightarrow \infty$ (using Proposition 2.19 and Corollary 2.17), we obtain (7.1) as desired. $\square$

## A Decomposition of the Coupled Spin Configuration

The main result in this section is the following proposition, which gives a decomposition of $\sigma \, {:}{=}\, \hat{\sigma }|_{E_{\sigma ,\sigma '}} + {\hat{\sigma }}'|_{C_1(B_N)\smallsetminus E_{{\hat{\sigma }},{\hat{\sigma }}'}}$ in terms of decompositions of $\hat{\sigma }$ and ${\hat{\sigma }}'.$

### Proposition 8.1

Let ${\hat{\sigma }} \in \Omega ^1(B_N,G)$ and ${\hat{\sigma }}' \in \Omega ^1_0(B_N,G),$ let ${\hat{\Sigma }}$ be a decomposition of ${\hat{\sigma }}$ and ${\hat{\Sigma }}'$ be a decomposition of $\hat{\sigma }'$ (these are guaranteed to exist by Lemma 2.6), and defineandThen $\Sigma \cup \Sigma '$ is a decomposition of $\sigma \, {:}{=}\, {\hat{\sigma }}|_{E_{\sigma ,\sigma '}} + \hat{\sigma }'|_{C_1(B_N)\smallsetminus E_{{\hat{\sigma }},{\hat{\sigma }}'}}.$

### Proof

We need to show that ((i))–((v)) of Lemma 2.6 holds, i.e. that

(i) If , then  is non-trivial and irreducible,
(ii) If , then ,
(iii) If , then  and  have disjoint supports,
(iv), and
(v) if , then  and  have disjoint supports,

We now show that (i)–(v) holds.

(i) Since ${\hat{\Sigma }}$ and ${\hat{\Sigma }}'$ are decompositions of ${\hat{\sigma }}$ and ${\hat{\sigma }}'$ respectively, (i) holds with ${\hat{\Sigma }} \cup \hat{\Sigma }'$ replaced with $\Sigma \cup \Sigma '.$ Since $\Sigma \cup \Sigma ' \subseteq {\hat{\Sigma }} \cup {\hat{\Sigma }}'$, the desired conclusion follows.
(ii) Fix some . By the definition of $\Sigma$, we have  and hence . At the same time, since $\Sigma \subseteq {\hat{\Sigma }}$ and ${\hat{\Sigma }}$ is a decomposition of ${\hat{\sigma }}$, we have  Finally, note that, by Lemma 5.9, we have $E_{{\hat{\sigma }},\hat{\sigma }'} = E_{\sigma ,{\hat{\sigma }}'}.$ By applying Lemma 5.2 twice, we obtain  and hence  Since proof in the case  is analogous, we omit it here.
(iii) Since ${\hat{\Sigma }}$ is a decomposition of ${\hat{\sigma }}$, for any distinct ,  and  have disjoint supports. Analogously, since ${\hat{\Sigma }}'$ is a decomposition of $\hat{\sigma }'$, for any distinct ,  and  have disjoint supports. Finally, if  and , then, since ,  and the sets $E_{\sigma ,\sigma '}$ and $C_1(B_N)\smallsetminus E_{\sigma ,\sigma '}$ are disjoint, it follows that  and  have disjoint supports. This concludes the proof of (iii).
(iv) Since ${\hat{\Sigma }}$ is a spin decomposition of $\hat{\sigma }$, each  is non-trivial and irreducible. Consequently, using Lemma 5.6, it follows that for each , we have either  or  and hence  Completely analogously, we find that  Combining the previous equations and using the definition of $\sigma$, we obtain (iv).
(v) If , then, since $\Sigma ' \subseteq {\hat{\Sigma }}'$ and ${\hat{\Sigma }}'$ is a decomposition of ${\hat{\sigma }}'$, we have . Since ${\hat{\sigma }}' \in \Omega ^1_0(B_N,G)$, we have $d\hat{\sigma }' = 0,$ and hence  Consequently, the desired conclusion will follow if we can show that (v) holds with $\Sigma \cup \Sigma '$ replaced with $\Sigma .$ To see that this holds, let . Then, since $\Sigma \subseteq {\hat{\Sigma }}$, we also have  Since ${\hat{\Sigma }}$ is a decomposition of ${\hat{\sigma }},$ the 2-forms  and  must have disjoint support. This concludes the proof of (v).

$$\square $$

## Disturbing 1-Forms

The main purpose of this section is to introduce the following definition.

### Definition 9.1

Let $\sigma \in \Omega ^1(B_N,G)$, and let $\gamma \in C^1(B_N)$ be a path. If there is no path ${\hat{\gamma }} \in C^1(B_N)$ with $\partial {\hat{\gamma }} = - \partial \gamma$ and 1-form  such that

(i)
(ii),
(iii),
(iv) Any vortex $\nu$ in  is a minimal vortex centered around an edge in $\gamma - \gamma _c$ (see (3.8) for a definition of $\gamma _c$), and
(v) If $d\sigma (p) = d\sigma (p')$ for all $p,p' \in {\hat{\partial }} e,$ then  for all $p \in {\hat{\partial }} e,$

then we say that $\sigma$ *disturbs* $\gamma$.

Note that if $\gamma \in C^1(B_N)$ is a generalized loop and $\sigma \in \Omega ^1(B_N,G),$ then we can pick ${\hat{\gamma }} = 0$ in Definition 9.1, and hence, in this case, (ii) automatically holds.

The main reason for introducing the previous definition is Lemma 9.2 below. To simplify the notation in this lemma, we define

9.1 $$\begin{aligned} \gamma '[e] \, {:}{=}\, (\gamma - \gamma _c)[e] \cdot \mathbb {1}\bigl ( \exists p,p' \in {\hat{\partial }} e :d\sigma (p) \ne d\sigma (p') \bigr ),\quad e \in C_1^+(B_N). \end{aligned}$$

### Lemma 9.2

Let $\sigma \in \Omega ^1(B_N,G)$ and let $\gamma \in C^1(B_N)$ be a path. For each $e \in \gamma$, fix one plaquette $p_e \in {\hat{\partial }} e.$

Then, if $\sigma$ does not disturb $\gamma$, we have

$$\begin{aligned} \sigma (\gamma ) = \sum _{e \in (\gamma -\gamma _c)- \gamma '} d\sigma (p_e). \end{aligned}$$

### Proof

Assume that $\sigma$ does not disturb $\gamma$. Then, by definition, there is ${\hat{\gamma }}$ and  which satisfies (i)–(iv) of Definition 9.1.

To simplify notation, define  ThenSince $\partial (\gamma + {\hat{\gamma }}) = \partial \gamma + \partial {\hat{\gamma }} = \partial \gamma - \partial \gamma = 0,$ $\gamma + {\hat{\gamma }}$ is a generalized loop. Let *B* be a cube of width $|{{\,\textrm{supp}\,}}(\gamma + {\hat{\gamma }})|$ which contains $\gamma + {\hat{\gamma }}$. Since $\gamma + \hat{\gamma }\subseteq C^1(B_N)$, such a cube exists. Next, let *q* be an oriented surface inside *B* such that $\gamma + {\hat{\gamma }}$ is the boundary of *q*. The existence of such a surface is guaranteed by Lemma 2.15.

By Lemma 2.6, there is a set $\Omega \subseteq \Omega _2^0(B_N,G)$ which is a decomposition of $d{\bar{\sigma }}$. Fix such a set $\Omega$, and note that, by definition, each $\omega \in \Omega$ is a vortex in ${\bar{\sigma }}$. Let $\Omega ^q$ be the set of all $\omega \in \Omega$ with $\omega (q) \ne 0$. Then, by the discrete Stokes’ theorem, we have

$$\begin{aligned} {\bar{\sigma }}(\gamma + {\hat{\gamma }}) = d{\bar{\sigma }}(q) = \sum _{\omega \in \Omega ^q} \omega (q). \end{aligned}$$

Now fix some $\omega \in \Omega ^q$. Since $\omega (q) \ne 0$, by (iv) and Lemma 2.12, there must exist $e \, {:}{=}\, \frac{\partial }{\partial x^j}\big |_a \in \Omega _1^+(B_N)$ and $g \in G\smallsetminus \{ 0\}$ such that $\gamma [e] = 1$ and $\omega = d(g \mathbb {1}_{a}\, d x_j)$. Then, by definition, we have $\omega (p_e) = g$, and since $\omega \le d{\bar{\sigma }}$ and $g \ne 0$, it follows that $d{\bar{\sigma }}(p_e) = \omega (p_e) = g$. Since *q* is an oriented surface with boundary $\gamma$, we thus have

$$\begin{aligned} \omega (q) = d(g\mathbb {1}_{a}\, dx_j)(q) = (g\mathbb {1}_{a}\, dx_j)(\gamma ) = g = \omega (p_e) = d{\bar{\sigma }}(p_e). \end{aligned}$$

DefineThen, since minimal vortices around distinct edges in $\gamma -\gamma _c$ have disjoint supports, it follows that

$$\begin{aligned} \sum _{\omega \in \Omega ^q} \omega (q) = \sum _{e \in \gamma _5} d{\bar{\sigma }}(p_e). \end{aligned}$$

Since, by assumption, we have , and , it follows from Lemma 2.3 (iv) that $d{\bar{\sigma }} \le d\sigma$.

Using the definition of $\gamma _5$, it follows that for any $e \in \gamma _5$, we have $d{\bar{\sigma }}(p_e) = d\sigma (p_e).$

Consequently,

$$\begin{aligned} \sum _{e \in \gamma _5} d{\bar{\sigma }}(p_e) = \sum _{e \in \gamma _5} d\sigma (p_e). \end{aligned}$$

Now note that by the definition of $\gamma ',$ we have

$$\begin{aligned} \bigl ( (\gamma -\gamma _c) - \gamma ')[e]  &   = (\gamma -\gamma _c)[e] \cdot \mathbb {1} \bigl ( d\sigma (p) = d\sigma (p') \text { for all } p,p' \in {\hat{\partial }} e \bigr ),\\  &   \qquad e \in C_1^+(B_N). \end{aligned}$$

Since $d{\bar{\sigma }} \le d\sigma$, it follows that if $e \in \gamma _5$ then $e \in (\gamma -\gamma _c) - \gamma '.$ Finally, we note that if $e \in (\gamma -\gamma _c) - \gamma ',$ then , and hence $d{\bar{\sigma }}(p_e)= d\sigma (p_e)=0.$ As a consequence,

$$\begin{aligned} \sum _{e \in \gamma _5} d\sigma (p_e) = \sum _{e \in (\gamma -\gamma _c) - \gamma '} d\sigma (p_e). \end{aligned}$$

By combining the previous equations, we obtain the desired conclusion. $\square$

## Proof of the Main Result

In this section, we will first give a proof of the following result, which is more general that Theorem 1.1, and then show how this proof, with very small adjustments, implies Theorem 1.1.

### Theorem 10.1

Let $G = {\mathbb {Z}}_n$ for some $n \ge 2$, let $\beta ,\kappa \ge 0$ satisfy [A], let $\gamma$ be a path, and let $\gamma _0 \in C^1(B_N)$ be any path with $\partial \gamma _0 = -\partial \gamma .$ Then

10.1 $$\begin{aligned} \begin{aligned}&\Bigl | \bigl \langle L_\gamma (\sigma ,\phi ) \bigr \rangle _{\beta ,\kappa ,\infty } - \Theta _{\beta ,\kappa }(\gamma ) H_\kappa (\gamma ) \Bigr | \\&\qquad \le K_6 \Biggl ( \alpha _2(\beta ,\kappa ) + \sqrt{\frac{\max (1,|{{\,\textrm{supp}\,}}\gamma _c|)}{|{{\,\textrm{supp}\,}}\gamma |}} \Biggr )^{|{{\,\textrm{supp}\,}}(\gamma -\gamma _c)|/(|{{\,\textrm{supp}\,}}(\gamma -\gamma _c)|+2|{{\,\textrm{supp}\,}}\gamma | )}, \end{aligned}\nonumber \\ \end{aligned}$$

where10.2where $$K_2$$ is given by (6.4), where $$K_3$$ and $$K_4$$ are given by (6.6), $$ K_5$$ is given by (6.7), $$K_7$$ is given by (10.21), $$K_8$$ is given by (10.22), and $$K_{10}$$ is given by (10.24).

### Remark 10.2

Using the equations in the beginning of Sect. 10.9, together with (10.39), one easily shows that if $G = {\mathbb {Z}}_2$, then

$$\begin{aligned} \begin{aligned} K_6&= 2^{2|{{\,\textrm{supp}\,}}\gamma |/(2|{{\,\textrm{supp}\,}}\gamma |+|{{\,\textrm{supp}\,}}(\gamma -\gamma _c)| )} \\&\quad \ \cdot \Biggr [ 2K_3 K_4 ^8\alpha _0(\kappa )^7 \sum _{e \in \gamma } \bigl ( K_4 \alpha _0(\kappa ) \bigr )^{\max (0,{{\,\textrm{dist}\,}}_0(e,\gamma _0)-8)} \\&\quad \ + K_2 + K_3 K_4^2 + \sqrt{ K_7 } + \sqrt{ K_8 } + \sqrt{K_{10} } + \sqrt{12 K_2} + 1 \Biggr ]^{|{{\,\textrm{supp}\,}}(\gamma -\gamma _c)|/(|{{\,\textrm{supp}\,}}(\gamma -\gamma _c)| + 2|{{\,\textrm{supp}\,}}\gamma |)}, \end{aligned} \end{aligned}$$

### A first application of the coupling

In this section, we split the expected value we are interested in into two parts, later corresponding to the two functions $\Theta _{\beta ,\kappa }(\gamma )$ and $H_\kappa (\gamma )$ in Theorem 10.1. In order to do this, we first define three useful events;

10.3 $$\begin{aligned}  &   {\mathcal {E}}_1 \, {:}{=}\, \bigl \{ ({\hat{\sigma }},{\hat{\sigma }}' ) \in \Omega ^1(B_N,G) \times \Omega ^1_0(B_N,G) :\exists \text { irreducible } {\bar{\sigma }} \le {\hat{\sigma }}'|_{E_{{\hat{\sigma }},\hat{\sigma }'}} \nonumber \\  &   \quad \text { that disturbs } \gamma \bigr \}, \end{aligned}$$

10.4 $$\begin{aligned}  &   {\mathcal {E}}_2 \, {:}{=}\, \bigl \{ ({\hat{\sigma }},{\hat{\sigma }}' ) \in \Omega ^1(B_N,G) \times \Omega ^1_0(B_N,G) :\exists \text { irreducible }{\bar{\sigma }} \le {\hat{\sigma }}|_{E_{{\hat{\sigma }},\hat{\sigma }'}}\nonumber \\    &   \quad \text { that disturbs } \gamma \bigr \}, \end{aligned}$$

and

10.5 $$\begin{aligned} \begin{aligned}&{\mathcal {E}}_3 \, {:}{=}\, \bigl \{ \sigma \in \Omega ^1(B_N,G) :\exists e \in \gamma ,\, {\tilde{\sigma }} \le \sigma ,\, {\tilde{\sigma }}' \le \sigma -{\tilde{\sigma }} \text { s.t. } d{\tilde{\sigma }}|_{\pm {{\,\textrm{supp}\,}}{\hat{\partial }} e} \ne 0 \\&\qquad \text { and } d\tilde{\sigma }'|_{\pm {{\,\textrm{supp}\,}}{\hat{\partial }} e} \ne 0 \bigr \}. \end{aligned} \end{aligned}$$

We provide upper bounds of the probabilities of these events occurring in Sect. 10.4.

Using this notation, we have the following result, which is the main result of this section.

#### Proposition 10.3

Let $\beta ,\kappa \ge 0$, and let $\gamma \in C^1(B_N)$ be a path. For each $e \in \gamma$, let $p_e \in {\hat{\partial }} e.$ Then

$$\begin{aligned} \begin{aligned}&\biggl | {\mathbb {E}}_{N,\beta ,\kappa }\bigl [ L_\gamma (\sigma ) \bigr ] - {\mathbb {E}}_{N,\infty ,\kappa } \bigl [ L_\gamma (\sigma ) \bigr ] \, {\mathbb {E}}_{N,\beta ,\kappa } \Bigl [ \, \prod _{e \in (\gamma - \gamma _c)- \gamma '} \rho \bigl (d \sigma (p_e)\bigr ) \Bigr ] \biggr |\\&\qquad \le 2\mu _{N,(\beta ,\kappa ),(\infty ,\kappa )} ( {\mathcal {E}}_1) + 2\mu _{N,(\beta ,\kappa ),(\infty ,\kappa )} ( {\mathcal {E}}_2) + 2\mu _{N,(\beta ,\kappa ),(\infty ,\kappa )} ( {\mathcal {E}}_3). \end{aligned} \end{aligned}$$

#### Proof

Let ${\hat{\sigma }} \in \Omega ^1(B_N,G)$ and ${\hat{\sigma }}' \in \Omega ^1_0(B_N,G)$, and let

$$\begin{aligned} \sigma \, {:}{=}\, {\hat{\sigma }}|_{E_{{\hat{\sigma }},{\hat{\sigma }}'}} + \hat{\sigma }'|_{C_1(B_N) \smallsetminus E_{{\hat{\sigma }},{\hat{\sigma }}'}}. \end{aligned}$$

Let ${\hat{\Sigma }}$ be a decomposition of ${\hat{\sigma }},$

let ${\hat{\Sigma }}'$ be a decomposition of ${\hat{\sigma }}',$ and defineandNote that these sets depend on ${\hat{\sigma }}$ and ${\hat{\sigma }}'$. Note also that if $({\hat{\Sigma }}' \smallsetminus \Sigma ') \cap \hat{\Sigma }_{bad}' \ne \emptyset ,$ then $({\hat{\sigma }},{\hat{\sigma }}') \in {\mathcal {E}}_1$, and if $\Sigma \cap {\hat{\Sigma }}_{bad} = \emptyset ,$ then $({\hat{\sigma }},{\hat{\sigma }}') \in {\mathcal {E}}_2$. By Proposition 8.1, $\Sigma \cup \Sigma '$ is a decomposition of $\sigma .$ This implies in particular that the 1-forms in $\Sigma \cup \Sigma '$ have disjoint supports, and hence10.6If $({\hat{\sigma }},{\hat{\sigma }}') \notin {\mathcal {E}}_1,$ then $(\hat{\Sigma }' \smallsetminus \Sigma ') \cap {\hat{\Sigma }}_{bad}' = \emptyset .$ Since  for all  and hence, using Lemma 9.2, it follows that, on this event, we haveand henceIn particular, this shows that10.7Next, note that since the 1-forms in $\Sigma$ have disjoint supports, we haveFor  defineIf $({\hat{\sigma }},{\hat{\sigma }}') \notin {\mathcal {E}}_2,$ then we have $\Sigma \cap {\hat{\Sigma }}_{bad} = \emptyset .$ Consequently, for any  we can apply Lemma 9.2 to obtainIf , then . Consequently, if $({\hat{\sigma }},{\hat{\sigma }}') \notin {\mathcal {E}}_2,$ thenWe now make a few observations.

- If  satisfies  for some  and $p \in \hat{\partial }e$, then, since ${\hat{\Sigma }}$ is a decomposition of ${\hat{\sigma }}$, we must have  for all
- If  then, since $\hat{\Sigma }$ is a decomposition of ${\hat{\sigma }}$, we must have  Consequently, if  for some $p \in C_2(B_N)$, then
- If  are distinct and  then either  or  for all $p \in {\hat{\partial }} e.$

DefineCombining these observations, it follows thatCombining the previous equations, it follows that if $(\hat{\sigma },{\hat{\sigma }}') \notin {\mathcal {E}}_2$, we haveand hence10.8We now argue that if $\gamma '_{{\hat{\sigma }}} \ne \gamma ''',$ then the event ${\mathcal {E}}_3$ must happen. To this end, first assume that $e \in \gamma _{{\hat{\sigma }}}'.$ Then there is $p,p' \in {\hat{\partial }} e$ with ${\hat{\sigma }} (p) \ne {\hat{\sigma }}(p').$ Without loss of generality, we can assume that ${\hat{\sigma }}(p) \ne 0$. Since ${\hat{\Sigma }}$ is a decomposition of ${\hat{\sigma }}$, there is  with  such that  Since  we must have either  or  Using the assumption that $d\hat{\sigma }(p) \ne 0,$ it follows that , and hence $e \in \gamma '''.$

Now, instead assume that $e \in \gamma '''-\gamma '.$ Then, since $e \in \gamma '''$, there must exist $p,p' \in {\hat{\partial }} e$ and  such that  Without loss of generality, we can assume that  Since  we have  and hence, since  it follows that 

Since  we must have either  or  Since $e \in \gamma ',$ we have $d{\hat{\sigma }}(p) = d{\hat{\sigma }}(p'),$ and hence, since  and  we conclude that 

Since $d{\hat{\sigma }}(p') \ne 0$ and  there must exist  such that 

To sum up, we have showed that if $e \in \gamma '''- \gamma ,$ then there are distinct  such that  and  Since  are distinct, using Lemma 2.8 we conclude that if $\gamma '_{\hat{\sigma }} \ne \gamma '''$, then ${\mathcal {E}}_3$ holds.

Consequently,

10.9 $$\begin{aligned} {\mathbb {E}}_{N,(\beta ,\kappa ),(\infty ,\kappa )}\Biggl ( \biggl | \prod _{e \in (\gamma -\gamma _c)- \gamma '''} \rho \bigl (d{\hat{\sigma }}(p_e)\bigr ) - \prod _{e \in (\gamma -\gamma _c)- \gamma '} \rho \bigl (d{\hat{\sigma }}(p_e)\bigr ) \biggr | \Biggr ) \le 2 \mu _{N,\beta ,\kappa }( {\mathcal {E}}_3).\nonumber \\ \end{aligned}$$

Combining (10.6), (10.7),  (10.8), and 10.9 we obtainThis concludes the proof. $\square$

### A resampling trick

Recall that given a path $\gamma$ and $\sigma \in \Omega ^1(B_N,G),$ we have let

$$\begin{aligned} \gamma '[e] = (\gamma -\gamma _c)[e] \cdot \mathbb {1}\bigl ( \exists p,p' \in {\hat{\partial }} e :d\sigma (p) \ne d\sigma (p') \bigr ),\quad e \in C_1(B_N), \end{aligned}$$

In this section, we describe a resampling trick, first introduced (in a different setting) in [9].

#### Proposition 10.4

[Proposition 10.1 in [18]]. Let $\beta ,\kappa \ge 0$, and let $\gamma \in C^1(B_N)$ be a path such that ${{\,\textrm{dist}\,}}_0(\gamma ,\partial C_1(B_N))\ge 8$. For each $e \in \gamma$, fix one plaquette $p_e \in {\hat{\partial }} e$. Then

10.10 $$\begin{aligned} {\mathbb {E}}_{N,\beta , \kappa }\biggl [ \rho \Bigl ( \sum _{e \in (\gamma -\gamma _c) - \gamma '} d\sigma (p_e) \Bigr ) \biggr ] = {\mathbb {E}}_{N,\beta , \kappa } \Bigl [ \, \prod _{ e \in (\gamma -\gamma _c) - \gamma '} \theta _{\beta ,\kappa } \bigl ( \sigma (e) - d\sigma (p_e) \bigr ) \Bigr ].\nonumber \\ \end{aligned}$$

For a proof of Proposition 10.4, we refer the reader to [18, Proposition 10.1]. In [18, Proposition 10.1], $\gamma$ is assumed to be a generalized loop rather than a path as in Proposition 10.4. However, since the proofs in the two cases are identical, we do not include a proof here.

### A second application of the coupling

In this section, we take the next step towards the proof of Theorem 10.1, by giving an upper bound on the distance between the right hand side of (10.10) and $\Theta '_{N,\beta ,\kappa }(\gamma ).$ To this end, using the notation of Sect. 5, we now introduce a few additional useful events which will be used to express the upper bound in the Proposition 10.5 below.

Given an edge $e \in C_1(B_N)$, let

10.11 $$\begin{aligned}  &   {\mathcal {E}}_4(e) \, {:}{=}\, \bigl \{ (\sigma ,\sigma ')\in \Omega ^1(B_N,G)\times \Omega ^1_0(B_N,G) :e \in E_{\sigma ,\sigma '} \text { and }\sigma '(e) \ne 0 \bigr \}, \end{aligned}$$

10.12 $$\begin{aligned}  &   {\mathcal {E}}_5(e) \, {:}{=}\, \bigl \{ (\sigma ,\sigma ') \in \Omega ^1(B_N,G) \times \Omega ^1_0(B_N,G) :\exists e' \in E_{\sigma ,\sigma '} \text { s.t. } {\hat{\partial }} e' \cap {\hat{\partial }} e \ne \emptyset \nonumber \\  &   \qquad \text { and } \exists g \in G\smallsetminus \{ 0 \} \text { s.t. } \sigma (e) - d\sigma (p) = g \, \forall p \in {\hat{\partial }} e \bigr \}, \end{aligned}$$

10.13 $$\begin{aligned}  &   {\mathcal {E}}_6(e) \, {:}{=}\, \bigl \{ \sigma ' \in \Omega ^1_0(B_N,G) :\sigma '(e) \ne 0 \bigr \}, \end{aligned}$$

10.14 $$\begin{aligned}  &   {\mathcal {E}}_7(e) \, {:}{=}\, \bigl \{ (\sigma ,\sigma ') \in \Omega ^1(B_N,G) \times \Omega ^1_0(B_N,G) :\exists p,p' \in {\hat{\partial }} e \text { s.t. } d\sigma (p) \ne d\sigma (p') \}.\nonumber \\ \end{aligned}$$

#### Proposition 10.5

Let $\beta ,\kappa \ge 0$, and let $\gamma \in C^1(B_N)$ be a path such that for all $e \in \gamma$, $p \in {\hat{\partial }} e$ and $p' \in \partial C_2(B_N),$ we have ${{\,\textrm{supp}\,}}\partial p \cap {{\,\textrm{supp}\,}}{\hat{\partial }} p' = \emptyset .$

Then

10.15 $$\begin{aligned} \begin{aligned}&\biggl | {\mathbb {E}}_{N,\beta , \kappa } \Bigl [ \, \prod _{ e \in (\gamma -\gamma _c) - \gamma '} \theta _{\beta ,\kappa } \bigl ( \sigma (e) - d\sigma (p_e) \bigr ) \Bigr ] - \Theta _{N,\beta ,\kappa }(\gamma ) \biggr | \\&\quad \le \, 2\sqrt{2 \alpha _4(\beta ,\kappa ) \sum _{e \in \gamma } \mu _{N,(\beta ,\kappa ),(\infty ,\kappa )}\bigl ({\mathcal {E}}_5(e)\bigr )} + 4\sqrt{2 \alpha _4(\beta ,\kappa ) \sum _{e \in \gamma } \mu _{N,(\beta ,\kappa ),(\infty ,\kappa )}\bigl ({\mathcal {E}}_4(e)\bigr )} \\  &\qquad + 2\sqrt{2 \alpha _4(\beta ,\kappa ) \sum _{e \in \gamma _c} \mu _{N,\infty ,\kappa }\bigl ({\mathcal {E}}_6(e)\bigr )} + 2\sqrt{2|{{\,\textrm{supp}\,}}\gamma _c| \alpha _3(\beta ,\kappa )} \\  &\qquad + 2\sqrt{2 \alpha _3(\beta ,\kappa ) \sum _{e \in \gamma } \mu _{N,(\beta ,\kappa ),(\infty ,\kappa )} \bigl ({\mathcal {E}}_7(e)\bigr )}. \end{aligned} \end{aligned}$$

For the proof of Proposition 10.5 we need a two lemmas from [18], which we now recall.

#### Lemma 10.6

[Lemma 11.2 in [18]]. Assume that $z_1,z_2,z_1',z_2' \in {\mathbb {C}}$ are such that $|z_1|,|z_2|,|z_1'|,|z_2'| \le 1$. Then

$$\begin{aligned} |z_1z_2-z_1'z_2'| \le |z_1-z_1'| + |z_2-z_2'|. \end{aligned}$$

#### Lemma 10.7

[Lemma 11.3 in [18]]. Let $a,b>0$. Assume that $A \subseteq C_1(B_N)$ is a random set with ${\mathbb {E}}[|A|] \le a$, and that

(i) $X_e \in {\mathbb {C}}$ and $|X_e| \le 1$ for all $e \in C_1(B_N)$, and
(ii) there exists a $c \in [-1,1]$ such that $|X_e-c| \le b$ for all $e \in C_1(B_N)$.

Then

$$\begin{aligned} {\mathbb {E}}\biggl [ \Bigl | \prod _{e \in A} c - \prod _{e \in A} X_e \Bigr | \biggr ] \le 2\sqrt{2ab}. \end{aligned}$$

#### Proof of Proposition 10.5

Recall the coupling $(\sigma ,\sigma ') \sim \mu _{N,(\beta ,\kappa ),(\infty ,\kappa )}$ between $\sigma \sim \mu _{N,\beta ,\kappa }$ and $\sigma ' \sim \mu _{N,\infty ,\kappa }$ described in Sect. 5.4, and the set $E_{\sigma ,\sigma '}$ defined in (5.16). Since $\mu _{N,(\beta ,\kappa ),(\infty ,\kappa )}$ is a coupling of $\mu _{N,\beta ,\kappa }$ and $\mu _{N,\infty ,\kappa }$, we have

$$\begin{aligned} \begin{aligned}&{\mathbb {E}}_{N,\beta , \kappa } \Bigl [\, \prod _{ e \in (\gamma -\gamma _c) - \gamma '} \theta _{\beta ,\kappa } \bigl ( \sigma (e) - d\sigma (p_e) \bigr ) \bigr ] - {\mathbb {E}}_{N,\infty ,\kappa } \Bigl [ \, \prod _{e \in \gamma } \theta _{\beta ,\kappa }\bigl (\sigma (e)\bigr ) \Bigr ] \\&\qquad = {\mathbb {E}}_{N,(\beta ,\kappa ),(\infty ,\kappa )} \Bigl [\, \prod _{ e \in (\gamma -\gamma _c) - \gamma '} \theta _{\beta ,\kappa }\bigl ( \sigma (e) - d\sigma (p_e) \bigr ) - \prod _{e \in \gamma } \theta _{\beta ,\kappa }\bigl (\sigma '(e)\bigr ) \Bigr ]. \end{aligned} \end{aligned}$$

Given $$(\sigma ,\sigma ') \in \Omega ^1(B_N,G) \times \Omega ^1_0(B_N,G)$$, define

$$\begin{aligned} \gamma ''[e] \, {:}{=}\, (\gamma -\gamma _c)[e] \cdot \mathbb {1} \bigl (\exists e' \in E_{\sigma ,\sigma '} :{\hat{\partial }} e' \cap {\hat{\partial }} e \ne \emptyset \bigr ), \quad e \in C_1(B_N)^+. \end{aligned}$$

In other words, $\gamma '$ is the indicator function for all edges is $\gamma -\gamma _c$ that is adjacent to some edge in $E_{\sigma ,\sigma '}$. By Lemma 5.21, if $e \in (\gamma -\gamma _c) - \gamma ''$, then $\sigma (e') = \sigma '(e')$ whenever $e' \in \partial p$ for some $p \in {\hat{\partial }} e$, and hence $d\sigma (p_e) = d\sigma '(p_e) = 0$. In particular, this implies that if $e \in (\gamma -\gamma _c) - \gamma '',$ then

10.16 $$\begin{aligned} \sigma (e)- d\sigma (p_e) = \sigma '(e)- d\sigma '(p_e) = \sigma '(e)- 0 = \sigma '(e). \end{aligned}$$

By the definition of $\gamma '$, if $e' \in \gamma '$ then there exists $p' \in {\hat{\partial }} e'$ and $e'' \in \partial p'$ such that $e'' \in \{ e''' \in {{\,\textrm{supp}\,}}{\sigma } :d\sigma |_{\pm {{\,\textrm{supp}\,}}{\hat{\partial }} e'''} \ne 0 \} \subseteq E_{\sigma , \sigma '}$. Consequently, there is $e'' \in E_{\sigma , \sigma '}$ such that ${\hat{\partial }} e'' \cap {\hat{\partial }} e \ne \emptyset .$

Hence, if $e \in \gamma '$ then $e\in \gamma '',$ and it follows that

$$\begin{aligned} \begin{aligned}&\prod _{ e \in (\gamma -\gamma _c) - \gamma '} \theta _{\beta ,\kappa } \bigl ( \sigma (e)- d\sigma (p_e) \bigr ) = \prod _{ e \in (\gamma -\gamma _c) - \gamma ''} \theta _{\beta ,\kappa } \bigl (\sigma (e)- d\sigma (p_e) \bigr ) \\&\qquad \prod _{ e \in \gamma '' - \gamma '} \theta _{\beta ,\kappa } \bigl ( \sigma (e)- d\sigma (p_e) \bigr ) \\  &\qquad \overset{(10.16)}{=} \prod _{ e \in (\gamma -\gamma _c) - \gamma '} \theta _{\beta ,\kappa }\bigl (\sigma '(e)\bigr ) \prod _{ e \in \gamma '' - \gamma '} \theta _{\beta ,\kappa } \bigl (\sigma (e)- d\sigma (p_e) \bigr ). \end{aligned} \end{aligned}$$

Consequently, using Lemma 5.21, we have

$$\begin{aligned}&\prod _{ e \in (\gamma -\gamma _c) - \gamma '} \theta _{\beta ,\kappa } \bigl ( \sigma (e)- d\sigma (p_e) \bigr ) - \prod _{ e \in \gamma } \theta _{\beta ,\kappa } \bigl ( \sigma '(e) \bigr ) \\&\quad = \prod _{ e \in (\gamma -\gamma _c) - \gamma ''} \theta _{\beta ,\kappa } \bigl (\sigma (e)\bigr ) \prod _{ e \in \gamma '' - \gamma '} \theta _{\beta ,\kappa } \bigl ( \sigma (e)- d\sigma (p_e) \bigr ) \\&\qquad - \prod _{ e \in (\gamma -\gamma _c) - \gamma '' } \theta _{\beta ,\kappa } \bigl (\sigma '(e)\bigr ) \prod _{ e \in \gamma '' - \gamma ' } \theta _{\beta ,\kappa } (\sigma '(e)) \prod _{ e \in \gamma _c + \gamma ' } \theta _{\beta ,\kappa } (\sigma '(e)) \\&\quad = \prod _{ e \in (\gamma -\gamma _c) - \gamma ''} \theta _{\beta ,\kappa } (\sigma '(e)) \biggl ( \, \prod _{ e \in \gamma '' - \gamma '} \theta _{\beta ,\kappa } \bigl ( \sigma (e)- d\sigma (p_e) \bigr ) - \prod _{ e \in \gamma '' - \gamma ' } \theta _{\beta ,\kappa } (\sigma '(e)) \biggr ) \\&\qquad + \prod _{ e \in (\gamma -\gamma _c) - \gamma ' } \theta _{\beta ,\kappa } \bigl (\sigma '(e)\bigr ) \Bigl ( 1-\prod _{ e \in \gamma _c } \theta _{\beta ,\kappa }(\sigma '(e))\Bigr ) \\&\qquad + \prod _{ e \in (\gamma -\gamma _c) - \gamma ' } \theta _{\beta ,\kappa } \bigl (\sigma '(e)\bigr ) \prod _{ e \in \gamma _c } \theta _{\beta ,\kappa }\bigl (\sigma '(e)\bigr ) \Bigl ( 1-\prod _{ e \in \gamma ' } \theta _{\beta ,\kappa }\bigl (\sigma '(e)\bigr )\Bigr ). \end{aligned}$$

Combining the above equations, we find that

$$\begin{aligned}&{\mathbb {E}}_{N,\beta , \kappa } \Bigl [\, \prod _{ e \in (\gamma -\gamma _c) - \gamma '} \theta _{\beta ,\kappa } \bigl ( \sigma (e) - d\sigma (p_e) \bigr ) \bigr ] - {\mathbb {E}}_{N,\infty ,\kappa } \Bigl [ \, \prod _{ e \in \gamma } \theta _{\beta ,\kappa } \bigl (\sigma (e)\bigr ) \Bigr ] \\  &\quad = {\mathbb {E}}_{N,(\beta ,\kappa ),(\infty ,\kappa )} \biggl [\, \prod _{ e \in (\gamma -\gamma _c) - \gamma ''} \theta _{\beta ,\kappa } (\sigma '(e)) \biggl ( \, \prod _{ e \in \gamma '' - \gamma '} \theta _{\beta ,\kappa } \bigl ( \sigma (e)- d\sigma (p_e) \bigr ) \\&\qquad - \prod _{ e \in \gamma '' - \gamma ' } \theta _{\beta ,\kappa } (\sigma '(e)) \biggr )\biggr ] \\  &\qquad + {\mathbb {E}}_{N,(\beta ,\kappa ),(\infty ,\kappa )} \biggl [\, \prod _{ e \in (\gamma -\gamma _c) - \gamma ' } \theta _{\beta ,\kappa } (\sigma '(e)) \Bigl ( 1-\prod _{ e \in \gamma _c } \theta _{\beta ,\kappa }(\sigma '(e))\Bigr ) \biggr ] \\&\qquad + {\mathbb {E}}_{N,(\beta ,\kappa ),(\infty ,\kappa )} \biggl [\,\prod _{ e \in (\gamma -\gamma _c) - \gamma '} \theta _{\beta ,\kappa } (\sigma '_e) \prod _{ e \in \gamma _c } \theta _{\beta ,\kappa }(\sigma '(e)) \Bigl ( 1-\prod _{ e \in \gamma ' } \theta _{\beta ,\kappa }(\sigma '(e))\Bigr )\bigg ]. \end{aligned}$$

Now note thatand similarly, thatandConsequently, by applying the triangle inequality and noting that, since $\rho$ is unitary, we have $|\theta _{\beta ,\kappa }(g)| \le 1$ for all $g \in G$, we obtain10.17We now use Lemma 10.7 to obtain upper bounds for each of the terms on the right-hand side of the previous equation.

#### Claim 10.8

10.18 $$\begin{aligned} {\mathbb {E}}_{N,(\beta ,\kappa ),(\infty ,\kappa )} \Bigl [ \bigl | \{ e \in \gamma '' :\sigma '(e) \ne 0 \} \bigr | \Bigr ] \le \sum _{e \in \gamma } \mu _{N,(\beta ,\kappa ),(\infty ,\kappa )} \bigl ( {\mathcal {E}}_4(e) \bigr ). \end{aligned}$$

#### Proof of claim

For any $e \in \gamma ''$, by definition, there is at least one $e' \in E_{\sigma ,\sigma '}$ such that ${{\hat{\partial }} e' \cap \hat{\partial }e \ne \emptyset .}$ Consequently, by the definition of $E_{\sigma ,\sigma '},$ if $\sigma '(e) \ne 0$, we $e \in E_{\sigma ,\sigma '}$.

From this it follows that

$$\begin{aligned} \begin{aligned}&{\mathbb {E}}_{N,(\beta ,\kappa ),(\infty ,\kappa )} \Bigl [ \bigl | \{ e \in \gamma '' :\sigma '(e) \ne 0 \} \bigr | \Bigr ] \\  &\qquad = \sum _{e \in \gamma ''} \mu _{N,(\beta ,\kappa ),(\infty ,\kappa )} \Bigl ( \bigl \{ (\sigma ,\sigma ')\in \Omega ^1(B_N,G)\times \Omega ^1_0(B_N,G) :\sigma '(e) \ne 0 \bigr \} \Bigr ) \\  &\qquad = \sum _{e \in \gamma ''} \mu _{N,(\beta ,\kappa ),(\infty ,\kappa )} \Bigl ( \bigl \{ (\sigma ,\sigma ')\in \Omega ^1(B_N,G)\times \Omega ^1_0(B_N,G) :e \in E_{\sigma ,\sigma '} \\&\qquad \text { and }\sigma '(e) \ne 0 \bigr \} \Bigr ) \\  &\qquad = \sum _{e \in \gamma ''} \mu _{N,(\beta ,\kappa ),(\infty ,\kappa )} \bigl ( {\mathcal {E}}_4(e) \bigr ) \le \sum _{e \in \gamma } \mu _{N,(\beta ,\kappa ),(\infty ,\kappa )} \bigl ( {\mathcal {E}}_4(e) \bigr ), \end{aligned} \end{aligned}$$

which is the desired conclusion.

#### Claim 10.9

10.19 $$\begin{aligned} {\mathbb {E}}_{N,(\beta ,\kappa ),(\infty ,\kappa )}\Bigl [ \bigl | \{ e \in \gamma '' - \gamma ' :\sigma (e) - d\sigma (p_e) \ne 0 \} \bigr |\Bigr ] \le \sum _{e \in \gamma }\mu _{N,(\beta ,\kappa ),(\infty ,\kappa )}\bigl ( {\mathcal {E}}_5(e)\bigr ).\nonumber \\ \end{aligned}$$

#### Proof of claim

Note fist that by definition, for any $e \in \gamma '' - \gamma ',$ we have

$$\begin{aligned} \bigl \{ (\sigma ,\sigma ') \in \Omega ^1(B_N,G) \times \Omega ^1_0(B_N,G) :\sigma (e) - d\sigma (p_e) \ne 0 \bigr \} = {\mathcal {E}}_5(e). \end{aligned}$$

Consequently,

$$\begin{aligned} \begin{aligned}&{\mathbb {E}}_{N,(\beta ,\kappa ),(\infty ,\kappa )}\Bigl [ \bigl | \{ e \in \gamma '' - \gamma ' :\sigma (e) - d\sigma (p_e) \ne 0 \} \bigr |\Bigr ] \\  &\qquad = \sum _{e \in \gamma '' - \gamma '}\mu _{N,(\beta ,\kappa ),(\infty ,\kappa )}\Bigl ( \bigl \{ (\sigma ,\sigma ') \in \Omega ^1(B_N,G) \times \Omega ^1_0(B_N,G) :\\  &\qquad \sigma (e) - d\sigma (p_e) \ne 0 \bigr \}\Bigr ) \\  &\qquad = \sum _{e \in \gamma '' - \gamma '}\mu _{N,(\beta ,\kappa ),(\infty ,\kappa )}\Bigl ( {\mathcal {E}}_5^4\Bigr ) \le \sum _{e \in \gamma }\mu _{N,(\beta ,\kappa ),(\infty ,\kappa )}\bigl ( {\mathcal {E}}_5(e)\bigr ), \end{aligned} \end{aligned}$$

which is the desired conclusion. $\square$

Next, by definition, we have

$$\begin{aligned} {\mathbb {E}}_{N,(\beta ,\kappa ),(\infty ,\kappa )} \Bigl [ \bigl | \{ e \in \gamma _c :\sigma '(e) \ne 0 \} \bigr | \Bigr ] = \sum _{e \in \gamma _c} \mu _{N,\infty ,\kappa } \bigl ( {\mathcal {E}}_6(e) \bigr ) \end{aligned}$$

and

$$\begin{aligned} \begin{aligned}&{\mathbb {E}}_{N,\beta ,\kappa }\bigl [|{{\,\textrm{supp}\,}}\gamma '|\bigr ] \\  &\qquad = \sum _{e \in \gamma \smallsetminus \gamma _c} \mu _{N,(\beta ,\kappa ),(\infty ,\kappa )} \Bigl ( \bigl \{ (\sigma ,\sigma ') \in \Omega ^1(B_N,G)\times \Omega ^1_0(B_N,G) :\exists p,p' \in {\hat{\partial }} e\\&\qquad \text { s.t. } d\sigma (p) \ne d\sigma (p') \bigr \} \Bigr ) \\  &\qquad = \sum _{e \in \gamma \smallsetminus \gamma _c} \mu _{N,(\beta ,\kappa ),(\infty ,\kappa )} \bigl ( {\mathcal {E}}_7(e)\bigr ) \le \sum _{e \in \gamma } \mu _{N, (\beta ,\kappa ),(\infty ,\kappa )} \bigl ( {\mathcal {E}}_7(e) \bigr ). \end{aligned} \end{aligned}$$

Finally, since for any $e \in \gamma '$ we also have $e \in \gamma ''$, we have

$$\begin{aligned} {\mathbb {E}}_{N,(\beta ,\kappa ),(\infty ,\kappa )}\Bigl [\bigl |\{e \in \gamma ' :\sigma '(e) \ne 0\}\bigr |\Bigr ] \le {\mathbb {E}}_{N,(\beta ,\kappa ),(\infty ,\kappa )}\Bigl [\bigl |\{e \in \gamma '' :\sigma '(e) \ne 0\}\bigr |\Bigr ], \end{aligned}$$

the right-hand side of which we have given an upper bound for in (10.18),

Applying Lemma 10.7 to the terms in (10.17), we thus obtain

$$\begin{aligned} \begin{aligned}&\biggl | {\mathbb {E}}_{N,\beta , \kappa } \Bigl [\, \prod _{ e \in (\gamma -\gamma _c) - \gamma '} \theta _{\beta ,\kappa } \bigl ( \sigma (e) - d\sigma (p_e) \bigr ) \bigr ] - {\mathbb {E}}_{N,\infty ,\kappa } \Bigl [ \, \prod _{ e \in \gamma } \theta _{\beta ,\kappa } \bigl (\sigma (e)\bigr ) \Bigr ] \biggr | \\&\quad \le 2\sqrt{2 \max _{g \in G} \bigl |\theta _{\beta ,\kappa }(g) - \theta _{\beta ,\kappa }(0)\bigr | \sum _{e \in \gamma } \mu _{N,(\beta ,\kappa ),(\infty ,\kappa )}\bigl ({\mathcal {E}}_5(e)\bigr )} \\  &\qquad + 4\sqrt{2 \max _{g \in G} \bigl |\theta _{\beta ,\kappa }(g) - \theta _{\beta ,\kappa }(0)\bigr | \sum _{e \in \gamma } \mu _{N,(\beta ,\kappa ),(\infty ,\kappa )}\bigl ({\mathcal {E}}_4(e)\bigr )} \\  &\qquad + 2\sqrt{2 \max _{g \in G} \bigl |\theta _{\beta ,\kappa }(g) - \theta _{\beta ,\kappa }(0)\bigr | \sum _{e \in \gamma _c} \mu _{N,\infty ,\kappa }\bigl ({\mathcal {E}}_6(e)\bigr )} \\  &\qquad + 2\sqrt{2|{{\,\textrm{supp}\,}}\gamma _c| \, \bigl |1 - \theta _{\beta ,\kappa }(0) \bigr |} + 2\sqrt{2 \bigl | 1 - \theta _{\beta ,\kappa }(0)| \sum _{e \in \gamma } \mu _{N,(\beta ,\kappa ),(\infty ,\kappa )} \bigl ({\mathcal {E}}_7(e)\bigr )}. \end{aligned} \end{aligned}$$

Recalling the definitions of $\alpha _3(\beta ,\kappa )$ and $\alpha _4(\beta ,\kappa )$, we obtain the desired conclusion. $\square$

### Upper bounds on events

In this section we provide upper bounds on the events ${\mathcal {E}}_1,$ ${\mathcal {E}}_2,$ and ${\mathcal {E}}_3,$ defined in Sect. 10.1, and the events ${\mathcal {E}}_4(e),$ ${\mathcal {E}}_5(e),$ ${\mathcal {E}}_6(e),$ and ${\mathcal {E}}_7(e),$ from Sect. 10.3.

#### Proposition 10.10

Let $\beta ,\kappa \ge 0$ be such that  [A] hold, let $\gamma \in C^1(B_N)$ be a path with ${{\,\textrm{dist}\,}}_0\bigl ({{\,\textrm{supp}\,}}\gamma ,\partial C_1(B_N)\bigr ) \ge 8$, let $\gamma _0 \in C^1(B_N)$ be any path such that $\partial \gamma _0 = -\partial \gamma ,$ and let ${\mathcal {E}}_1$ be given by (10.3). Then

$$\begin{aligned} \begin{aligned} \mu _{N,(\beta ,\kappa ),(\infty ,\kappa )}( {\mathcal {E}}_1)&\le \mathbb {1}(\partial \gamma \ne 0) \Bigl ( K_3 K_4^8\alpha _0(\kappa )^8\alpha _1(\beta )^6 \sum _{e \in \gamma } \bigl ( K_4 \alpha _0(\kappa ) \bigr )^{\max (0,{{\,\textrm{dist}\,}}_0(e,{{\,\textrm{supp}\,}}\gamma _0 )-8)} \\  &\quad + K_3 |{{\,\textrm{supp}\,}}\gamma |\bigl ( K_4 \alpha _0(\kappa ) \bigr )^{{{\,\textrm{dist}\,}}_1({{\,\textrm{supp}\,}}\gamma ,\partial C_1(B_N))}\Bigr ), \end{aligned} \end{aligned}$$

where $K_3$ and $K_4$ are given in (6.6).

#### Proposition 10.11

Let $\beta ,\kappa \ge 0$ be such that [A] hold, let $\gamma \in C^1(B_N)$ be a path such that $e \in \gamma$ and $p \in {\hat{\partial }} e$ we have ${{\,\textrm{dist}\,}}_0({{\,\textrm{supp}\,}}\partial p,\partial C_1(B_N))) \ge 8,$ let $\gamma _0 \in C^1(B_N)$ be any path such that $\partial \gamma _0 = -\partial \gamma ,$ and let ${\mathcal {E}}_2$ be given by (10.4). Then

$$\begin{aligned} \begin{aligned}&\mu _{N,(\beta ,\kappa ),(\infty ,\kappa )}( {\mathcal {E}}_2) \\  &\quad \le \mathbb {1}(\partial \gamma \ne 0)\cdot K_3 K_4^8\alpha _0(\kappa )^8\alpha _1(\beta )^6 \sum _{e \in \gamma } \bigl ( K_4 \alpha _0(\kappa ) \bigr )^{\max (0,{{\,\textrm{dist}\,}}_0(e,{{\,\textrm{supp}\,}}\gamma _0)-8)} \\  &\qquad + K_2 |{{\,\textrm{supp}\,}}\gamma _c|\alpha _2(\beta ,\kappa )^{6} + K_3 K_4^2 |{{\,\textrm{supp}\,}}\gamma | \alpha _0(\kappa )^2 \alpha _1(\beta )^{7} \\  &\qquad + 4K_3 |{{\,\textrm{supp}\,}}\gamma |\bigl ( K_4 \alpha _0(\kappa ) \bigr )^{{{\,\textrm{dist}\,}}_1({{\,\textrm{supp}\,}}\gamma ,\partial C_1(B_N))}, \end{aligned} \end{aligned}$$

where $K_2$ is defined in (6.4), and $K_3$ and $K_4$ are given in (6.6).

#### Proposition 10.12

Let $\beta ,\kappa \ge 0$ be such that [A] hold, let $\gamma \in C^1(B_N)$ be a path such for all $e \in \gamma$ and $p \in {\hat{\partial }} e$ we have ${{\,\textrm{dist}\,}}_0({{\,\textrm{supp}\,}}\partial p,\partial C_1(B_N)) \ge 8,$ and let ${\mathcal {E}}_3$ be defined by (10.5). Then

10.20 $$\begin{aligned} \mu _{N,\beta ,\kappa }({\mathcal {E}}_3) \le 18^4 K_5 |{{\,\textrm{supp}\,}}\gamma | \alpha _0(\kappa )^2 \alpha _1(\beta )^{12} \end{aligned}$$

where $K_5$ is given by (6.7).

#### Proposition 10.13

[Proposition 7.10 in [18]]. Let $\beta ,\kappa \ge 0$ be such that [A] holds, let $e \in C_1(B_N)$ be such that the support of ${\hat{\partial }} e$ contains no boundary plaquettes of $B_N,$ and let ${\mathcal {E}}_4(e)$ be given by (10.11).

Then

$$\begin{aligned} \mu _{N,(\beta ,\kappa ),(\infty ,\kappa )}\bigl ( {\mathcal {E}}_4(e) \bigr ) \le K_7 \alpha _0(\kappa )^9\alpha _1(\beta )^6 + K_3 \bigl ( K_4 \alpha _0(\kappa ) \bigr )^{{{\,\textrm{dist}\,}}_1(e,\partial C_1(B_N))}, \end{aligned}$$

where10.21and $K_3$ and $K_4$ are given by (6.6).

#### Proposition 10.14

[Proposition 7.12 in [18]]. Let $\beta ,\kappa \ge 0$ be such that [A] holds. Next, let $e \in C_1(B_N)$ be such that the support of $\hat{\partial }e$ contains no boundary plaquettes of $B_N,$ and let ${\mathcal {E}}_5(e)$ be given by (10.12).

Then

$$\begin{aligned} \mu _{N,(\beta ,\kappa ),(\infty ,\kappa )}\bigl ( {\mathcal {E}}_5(e) \bigr )  &   \le K_8 \alpha _1(\beta )^6 \alpha _0(\kappa )^6 \max \bigl ( \alpha _0(\kappa ), \alpha _1(\beta )^6\bigr )\\    &   \quad + K_9 \bigl ( K_4 \alpha _0(\kappa ) \bigr )^{{{\,\textrm{dist}\,}}_1(e,\partial C_1(B_N))}, \end{aligned}$$

where10.22and $K_3$ and $K_4$ are given by (6.6).

#### Proposition 10.15

[Proposition 7.14 in [18]]. Let $\beta ,\kappa \ge 0$ be such that [A] holds, let $e \in C_1(B_N)$ be such that ${{\,\textrm{dist}\,}}_0({{\,\textrm{supp}\,}}\partial p,\partial C_1(B_N)) \ge 8$ for all $p \in {\hat{\partial }} e,$ and let ${\mathcal {E}}_6(e)$ be given by (10.13).

Then

10.23 $$\begin{aligned} \mu _{N,\infty ,\kappa } \bigl ( {\mathcal {E}}_6(e) \bigr ) \le K_{10} \alpha _0(\kappa )^8, \end{aligned}$$

where

10.24 $$\begin{aligned} K_{10} \, {:}{=}\, 18^{13} \bigl (1-18^{2} \alpha _0(\kappa )\bigr )^{-1}. \end{aligned}$$

#### Proposition 10.16

Let $\beta ,\kappa \ge 0$ be such that [A] holds, let $e \in C_1(B_N)$ be such that for all $p \in {\hat{\partial }} e$, the support of $\partial p$ contains no boundary edges of $B_N,$ and let ${\mathcal {E}}_7(e)$ be given by (10.14). Then

$$\begin{aligned} \mu _{N,(\beta ,\kappa ),(\infty ,\kappa )}\bigl ( {\mathcal {E}}_7(e) \bigr ) \le 6 K_2 \, \alpha _2(\beta ,\kappa )^6, \end{aligned}$$

where $K_2$ is given by (6.4).

Before we provide proofs of Propositions 10.10, 10.11, 10.12, and  10.16, we state and prove the following lemma, which will be useful in these proofs.

#### Lemma 10.17

Let $\gamma \in C^1(B_N)$ be a path with $\partial \gamma \ne 0$ and ${{\,\textrm{dist}\,}}_0({{\,\textrm{supp}\,}}\gamma ,\partial C_1(B_N))) \ge 8,$ let $\sigma \in \Omega ^1_0(B_N,G)$ be such that the support of any path ${\hat{\gamma }} \in C^1(B_N)$ with $\partial {\hat{\gamma }} = - \partial \gamma$ intersects ${{\,\textrm{supp}\,}}\sigma .$ Then, for any $e \in {{\,\textrm{supp}\,}}\sigma$ and any path $\gamma _0 \in C^1(B_N)$ such that $\partial \gamma _0 = -\partial \gamma ,$ we have

$$\begin{aligned} \bigl |({{\,\textrm{supp}\,}}\sigma )^+ \bigr | \ge \max \bigl ({{\,\textrm{dist}\,}}_0(e,{{\,\textrm{supp}\,}}\gamma _0),8\bigr ). \end{aligned}$$

#### Proof

Since $\partial \gamma \ne 0$ and the support of any path $\hat{\gamma }\in C^1(B_N)$ with $\partial {\hat{\gamma }} = - \partial \gamma$ intersects ${{\,\textrm{supp}\,}}\sigma ,$ we must have ${{\,\textrm{supp}\,}}\sigma \ne \emptyset .$ Since $\sigma \in \Omega ^1_0(B_N,G),$ it thus follows from Lemma 2.9 that

$$\begin{aligned} \bigl |({{\,\textrm{supp}\,}}\sigma )^+ \bigr | \ge 8. \end{aligned}$$

Using the definition of $\gamma _0,$ the desired conclusion now follows. $\square$

#### Proof of Proposition 10.10

Assume first that $({\hat{\sigma }}, {\hat{\sigma }}') \in {\mathcal {E}}_1.$ Then there exists an irreducible 1-form  that disturbs $\gamma .$

By Lemma 5.2, we have $\hat{\sigma }'|_{E_{{\hat{\sigma }},{\hat{\sigma }}'}} \le {\hat{\sigma }}'$, and hence, since , it follows from Lemma 2.3 (iii) that  Since ${\hat{\sigma }}' \in \Omega ^1_0(B_N,G)$, we have $d{\hat{\sigma }}' = 0,$ and hence we conclude that 

Since  disturbs $\gamma ,$ the set  must be non-empty (since otherwise, we could let ${\hat{\gamma }} = -\gamma$ and  in Definition 9.1). Fix some edge 

Since  we have  Using the definition of $E_{{\hat{\sigma }},{\hat{\sigma }}'}$, we conclude that . Since  is irreducible and  Lemma 5.3 implies that  and hence $d\bigl ( {\hat{\sigma }}|_{ {\mathcal {C}}_{{\mathcal {G}}({\hat{\sigma }},{\hat{\sigma }}')}(e)} \bigr )\ne 0$. Since  is irreducible, satisfies , and disturbs $\gamma$, the support of any path ${\hat{\gamma }} \in C^1(B_N)$ with $\partial {\hat{\gamma }} = -\partial \gamma$ must intersect the support of . This implies in particular that we must have $\partial \gamma \ne 0.$ Applying Lemma 10.17, we thus obtain  and consequently $\bigl |{\mathcal {G}}_{{\mathcal {G}}({\hat{\sigma }},{\hat{\sigma }}')}(e) \bigr | \ge 2\max \bigl ({{\,\textrm{dist}\,}}_0(e,{{\,\textrm{supp}\,}}\gamma _0),8 \bigr )$. To sum up, we have showed thatApplying Proposition 6.1 with $M = \max \bigl ({{\,\textrm{dist}\,}}_0(e,{{\,\textrm{supp}\,}}\gamma _0),8\bigr )$ and $M' = 1$ for each $e \in \gamma$, we obtain (10.10) as desired. $\square$

#### Proof of Proposition 10.11

Assume first that $({\hat{\sigma }}, {\hat{\sigma }}') \in {\mathcal {E}}_2.$ Then there exists an irreducible 1-form  that disturbs $\gamma$. Since  disturbs $\gamma ,$ the set  must be non-empty (since otherwise, we could let ${\hat{\gamma }} = -\gamma$ and  in Definition 9.1). Fix some edge 

By definition, we must have . Using the definition of $E_{{\hat{\sigma }},{\hat{\sigma }}'}$, we see that  Since  and  is irreducible, it follows from Lemma 5.3 that  and hence $d\bigl ( {\hat{\sigma }}|_{ {\mathcal {C}}_{{\mathcal {G}}({\hat{\sigma }},{\hat{\sigma }}')}(e)} \bigr )\ne 0.$

Since  is irreducible and disturbs $\gamma$, we must be in one of the following four cases.

1. $\partial \gamma \ne 0,$ and all paths ${\hat{\gamma }} \in C^1(B_N)$ with $\partial {\hat{\gamma }} = - \partial \gamma$ intersects the support of . In this case, by Lemma 10.17, we must have  and consequently, $\bigl |{\mathcal {G}}_{{\mathcal {G}}({\hat{\sigma }},{\hat{\sigma }}')}(e) \bigr | \ge 2\max \bigl ({{\,\textrm{dist}\,}}_0(e,{{\,\textrm{supp}\,}}\gamma _0),8 \bigr ).$
contains a minimal vortex centered around some edge $e' \in \gamma _c.$ Since  by definition, and ${\hat{\sigma }}|_{E_{{\hat{\sigma }},{\hat{\sigma }}'}} \le {\hat{\sigma }}$ by Lemma 5.2, is follows from Lemma 2.11, applied twice, that $\hat{\sigma }$ also contains a minimal vortex centered around some edge $e' \in \gamma _c.$
In this case, by the same argument as above, we must have both ${\bigl | {{\,\textrm{supp}\,}}d{\hat{\sigma }}|_{{\mathcal {C}}_{{\mathcal {G}}({\hat{\sigma }},\hat{\sigma }')}(e)} \ge 2 \cdot 7 }$ and $|{\mathcal {C}}_{{\mathcal {G}}({\hat{\sigma }},{\hat{\sigma }}')}(e)| \ge 2 \cdot 2.$
supports a vortex $\nu$ with support at the boundary of $B_N.$ In this case, by the same argument as above, we must have $\bigl | {\mathcal {C}}_{{\mathcal {G}}({\hat{\sigma }},{\hat{\sigma }}')}(e) \bigr | \ge 2 \cdot {{\,\textrm{dist}\,}}_1(e,\partial C_1(B_N)).$

Consequently, we have showed thatBy first applying union bounds to all terms, and then using Proposition 6.1 to upper bound the first, third, and fourth term and Proposition 6.3 to upper bound the second term, we obtain

$$\begin{aligned} \begin{aligned}&\mu _{N,(\beta ,\kappa ),(\infty ,\kappa )}( {\mathcal {E}}_2) \\  &\quad \le \mathbb {1}(\partial \gamma \ne 0)\cdot K_3 K_4^8 \alpha _0(\kappa )^8\alpha _1(\beta )^6 \sum _{e \in \gamma } \bigl ( K_4 \alpha _0(\kappa ) \bigr )^{\max (0,{{\,\textrm{dist}\,}}_0(e,{{\,\textrm{supp}\,}}\gamma _0)-8)} \\  &\qquad + K_3 |{{\,\textrm{supp}\,}}\gamma |\bigl ( K_4 \alpha _0(\kappa ) \bigr )^{{{\,\textrm{dist}\,}}_0({{\,\textrm{supp}\,}}\gamma ,\partial C_1(B_N))} + K_2 |{{\,\textrm{supp}\,}}\gamma _c|\alpha _2(\beta ,\kappa )^{6} \\  &\qquad +K_3 K_4^2 |{{\,\textrm{supp}\,}}\gamma | \alpha _0(\kappa )^2 \alpha _1(\beta )^{7} \\  &\qquad + K_3 |{{\,\textrm{supp}\,}}\gamma |\bigl ( K_4 \alpha _0(\kappa ) \bigr )^{{{\,\textrm{dist}\,}}_1({{\,\textrm{supp}\,}}\gamma ,\partial C_1(B_N))} \alpha _1(\beta )^{6} \\  &\qquad + 2K_3|{{\,\textrm{supp}\,}}\gamma |\bigl ( K_4 \alpha _0(\kappa ) \bigr )^{{{\,\textrm{dist}\,}}_1({{\,\textrm{supp}\,}}\gamma ,\partial C_1(B_N))}. \end{aligned} \end{aligned}$$

Simplifying this expression, and noting that by definition, we have $\alpha _1(\beta )\le 1$, we obtain (10.11) as desired. This concludes the proof. $\square$

#### Proof of Proposition 10.12

Assume first that $\sigma \in {\mathcal {E}}_3,$ and let $\Sigma$ be a decomposition of $\sigma .$ Further, let $e \in \gamma ,$ $p,p' \in {\hat{\partial }} e,$ and  be such that   and  Without loss of generality we can assume that $e \in C_1(B_N)^+.$ By Lemma 2.8, we must then have  and  and hence 

Define $$E_e \, {:}{=}\, \bigl \{ e' \in C_1(B_N) :{\hat{\partial }} e' \cap {\hat{\partial }} e \ne \emptyset \bigr \}.$$

Since , there must exist $e' \in \partial p$ such that . Since , it follows that $\sigma (e') \ne 0$. Since $e' \in \partial p \subseteq E_e$, it follows that ${\mathcal {C}}_{{\mathcal {G}}(\sigma ,0)}(E_e)$ is non-empty. Moreover, since  is irreducible, using Lemma  5.5, it follows that  Completely analogously, we also obtain  Since , by Lemma (2.8), we must have  and hence $|{{\,\textrm{supp}\,}}d\sigma |_{{\mathcal {C}}_{{\mathcal {G}}(\sigma ,0)}(E_e)} )^+|\ge 2\cdot 6 = 12.$ Using Lemma 2.7, it follows that $\bigl |({\mathcal {C}}_{{\mathcal {G}}(\sigma ,0)}(E_e) )^+\bigr |\ge 12/6=2.$

Combining these observations and using a union bound, we see thatApplying Proposition 6.5 with $M = 2$ and $M' = 12,$ we obtain (10.20) as desired. $\square$

#### Proof of Proposition 10.16

Recall that

$$\begin{aligned} \begin{aligned}&{\mathcal {E}}_7(e) = \bigl \{ (\sigma ,\sigma ') \in \Omega ^1(B_N,G) \times \Omega ^1_0(B_N,G) :\exists p,p' \in {\hat{\partial }} e \text { s.t. } d\sigma (p) \ne d\sigma (p') \}. \end{aligned} \end{aligned}$$

On this event, there must exist some $p \in {\hat{\partial }} e$ with $d\sigma (p) \ne 0.$ Since $|{\hat{\partial }} e| = 6,$ together with a union bound, the desired conclusion follows from Proposition 6.3. $\square$

### A second version of our main result

In this section, we prove a second version of our main result, by giving a refinement of Proposition 7.1. While the error term in Proposition 7.1 corresponds to the probability of the event that no cluster in ${\mathcal {G}}({\hat{\sigma }},{\hat{\sigma }}')$ both intersects $\gamma$ and at the same time supports a vortex, the error term in Proposition 10.18 below essentially corresponds to the probability that no cluster in ${\mathcal {G}}({\hat{\sigma }},{\hat{\sigma }}')$ both intersects $\gamma$ and at the same time supports a non-minimal vortex.

#### Proposition 10.18

Let $\beta ,\kappa \ge 0$ be such that [A] hold, let $\gamma \in C^1(B_N)$ be a path such that ${{\,\textrm{dist}\,}}_0({{\,\textrm{supp}\,}}\gamma ,\partial C_1(B_N))\ge 8$ and such that for each $e \in \gamma$ the support of ${\hat{\partial }} e$ contains no boundary plaquettes of $B_N,$ and let $\gamma _0 \in C^1(B_N)$ be any path such that $\partial \gamma _0 = -\partial \gamma .$

Then

10.25 $$\begin{aligned} \begin{aligned}&\Bigl |{\mathbb {E}}_{N,\beta ,\kappa } \bigl [L_\gamma (\sigma )\bigr ]-{\mathbb {E}}_{N,\infty ,\kappa } \bigl [L_\gamma (\sigma )\bigr ] \,\Theta _{N,\beta ,\kappa }(\gamma ) \Bigr | \\  &\quad \le 2K_{11} |{{\,\textrm{supp}\,}}\gamma |\alpha _2(\beta ,\kappa )^6 + 2K_{12} \sqrt{2|{{\,\textrm{supp}\,}}\gamma | \alpha _2(\beta ,\kappa )^6}, \end{aligned}\nonumber \\ \end{aligned}$$

where

10.26 $$\begin{aligned} K_{11}&\, {:}{=}\, \, \mathbb {1}(\partial \gamma \ne 0) \cdot \frac{2K_3 K_4^8\alpha _0(\kappa )^8\alpha _1(\beta )^6 \sum _{e \in \gamma } \bigl ( K_4 \alpha _0(\kappa ) \bigr )^{\max (0,{{\,\textrm{dist}\,}}_0(e,{{\,\textrm{supp}\,}}\gamma _0)-8)}}{|{{\,\textrm{supp}\,}}\gamma |\alpha _2(\beta ,\kappa )^6}\nonumber \\&\quad + \, \frac{4K_3 \bigl ( K_4 \alpha _0(\kappa ) \bigr )^{{{\,\textrm{dist}\,}}_1({{\,\textrm{supp}\,}}\gamma ,\partial C_1(B_N))}}{\alpha _2(\beta ,\kappa )^6} \nonumber \\&\quad + \, \frac{ K_2 \, |{{\,\textrm{supp}\,}}\gamma _c|}{|{{\,\textrm{supp}\,}}\gamma | } + \frac{K_3 K_4^2\, \alpha _0(\kappa )^2 \alpha _1(\beta )^{7}}{\alpha _2(\beta ,\kappa )^6} + \frac{18^4 K_5 \alpha _0(\kappa )^2 \alpha _1(\beta )^{12}}{2\alpha _2(\beta ,\kappa )^6} \nonumber \\&\quad + \, \sqrt{\frac{2 K_8 \, \alpha _0(\kappa )^6\alpha _1(\beta )^6 \alpha _4(\beta ,\kappa ) \max \bigl ( \alpha _0(\kappa ), \alpha _1(\beta )^6\bigr )}{|{{\,\textrm{supp}\,}}\gamma | \alpha _2(\beta ,\kappa )^{12}}}\nonumber \\&\quad + \sqrt{\frac{2 K_9 \, \alpha _4(\beta ,\kappa ) \bigl ( K_3 \alpha _0(\kappa ) \bigr )^{{{\,\textrm{dist}\,}}_1({{\,\textrm{supp}\,}}\gamma ,\partial C_1(B_N))} }{|{{\,\textrm{supp}\,}}\gamma | \alpha _2(\beta ,\kappa )^{12}}} \nonumber \\&\quad + \, 2\sqrt{\frac{2K_7 \, \alpha _0(\kappa )^9\alpha _1(\beta )^6\alpha _4(\beta ,\kappa ) }{|{{\,\textrm{supp}\,}}\gamma | \alpha _2(\beta ,\kappa )^{12}}} \nonumber \\&\quad + 2\sqrt{\frac{2 K_3\, \alpha _4(\beta ,\kappa ) \bigl ( K_4 \alpha _0(\kappa ) \bigr )^{{{\,\textrm{dist}\,}}_1({{\,\textrm{supp}\,}}\gamma ,\partial C_1(B_N))}}{|{{\,\textrm{supp}\,}}\gamma | \alpha _2(\beta ,\kappa )^{12}}} \nonumber \\&\quad + \, \sqrt{\frac{12 K_2 \, \alpha _2(\beta ,\kappa )^6 \alpha _3(\beta ,\kappa )}{|{{\,\textrm{supp}\,}}\gamma | \alpha _2(\beta ,\kappa )^{12}} }, \end{aligned}$$

10.27 $$\begin{aligned} K_{12}&{:}{=}\, \sqrt{\frac{K_{10}\, |{{\,\textrm{supp}\,}}\gamma _c|\alpha _0(\kappa )^8 \alpha _4(\beta ,\kappa )}{|{{\,\textrm{supp}\,}}\gamma |\alpha _2(\beta ,\kappa )^6}} + \sqrt{\frac{|{{\,\textrm{supp}\,}}\gamma _c|\alpha _3(\beta ,\kappa )}{|{{\,\textrm{supp}\,}}\gamma |\alpha _2(\beta ,\kappa )^6} }. \end{aligned}$$

$K_2$ is given by (6.4), $K_3$ and $K_4$ are given by (6.6), $K_5$ is given by (6.7), $K_7$ is given by (10.21), $K_8$ and $K_9$ are given by (10.22), and $K_{10}$ is given by (10.24).

#### Proof

By using first the definition of $\Theta _{N,\beta ,\kappa }(\gamma ),$ and then the triangle inequality, we see that

$$\begin{aligned}&\Bigl |{\mathbb {E}}_{N,\beta ,\kappa } \bigl [L_\gamma (\sigma )\bigr ]-{\mathbb {E}}_{N,\infty ,\kappa } \bigl [L_\gamma (\sigma )\bigr ] \Theta _{N,\beta ,\kappa }(\gamma )\Bigr | \\  &\quad = \Bigl |{\mathbb {E}}_{N,\beta ,\kappa } \bigl [L_\gamma (\sigma )\bigr ]- {\mathbb {E}}_{N,\infty ,\kappa } \bigl [ L_\gamma (\sigma ) \bigr ] {\mathbb {E}}_{N,\infty ,\kappa } \Bigl [ \, \prod _{e \in \gamma } \theta _{\beta ,\kappa }(\sigma _e) \Bigr ] \Bigr | \\  &\quad \le \biggl | {\mathbb {E}}_{N,\beta ,\kappa } \bigl [ L_\gamma (\sigma ) \bigr ] - {\mathbb {E}}_{N,\infty ,\kappa } \bigl [ L_\gamma (\sigma ) \bigr ] \, {\mathbb {E}}_{N,\beta ,\kappa } \Bigl [ \, \prod _{e \in (\gamma -\gamma _c)- \gamma '} \rho \bigl (d\sigma (p_e)\bigr ) \Bigr ] \biggr | \\  &\qquad + \Bigl | {\mathbb {E}}_{N,\infty ,\kappa } \bigl [ L_\gamma (\sigma ) \bigr ] \Bigr | \cdot \biggl | {\mathbb {E}}_{N,\beta ,\kappa } \Bigl [ \, \prod _{e \in (\gamma -\gamma _c)- \gamma '} \rho \bigl (d\sigma (p_e)\bigr ) \Bigr ] \\  &\qquad - {\mathbb {E}}_{N,\beta , \kappa } \Bigl [ \, \prod _{ e \in (\gamma -\gamma _c) - \gamma '} \theta _{\beta ,\kappa } \bigl ( \sigma (e) - d\sigma (p_e) \bigr ) \Bigr ]\biggr |\\  &\qquad + \Bigl | {\mathbb {E}}_{N,\infty ,\kappa } \bigl [ L_\gamma (\sigma ) \bigr ] \Bigr | \cdot \biggl | {\mathbb {E}}_{N,\beta , \kappa } \Bigl [ \, \prod _{ e \in (\gamma -\gamma _c) - \gamma '} \theta _{\beta ,\kappa } \bigl ( \sigma (e) - d\sigma (p_e) \bigr ) \Bigr ] \\  &\qquad - {\mathbb {E}}_{N,\infty ,\kappa } \Bigl [ \, \prod _{e \in \gamma } \theta _{\beta ,\kappa }\bigl (\sigma (e)\bigr ) \Bigr ] \biggr |. \end{aligned}$$

Since $|L_\gamma (\sigma )| \le 1,$ we can apply Proposition 10.3, Proposition 10.4 and Proposition 10.5 in order to obtain

$$\begin{aligned}&\Bigl |{\mathbb {E}}_{N,\beta ,\kappa } \bigl [L_\gamma (\sigma )\bigr ]- {\mathbb {E}}_{N,\infty ,\kappa } \bigl [ L_\gamma (\sigma ) \bigr ] {\mathbb {E}}_{N,\infty ,\kappa } \Bigl [ \, \prod _{e \in \gamma } \theta _{\beta ,\kappa }(\sigma _e) \Bigr ] \Bigr | \\  &\quad \le 2\mu _{N,(\beta ,\kappa ),(\infty ,\kappa )} ( {\mathcal {E}}_1) + 2\mu _{N,(\beta ,\kappa ),(\infty ,\kappa )} ( {\mathcal {E}}_2) + 2\mu _{N,\beta ,\kappa }({\mathcal {E}}_3) \\  &\qquad + 2\sqrt{2 \alpha _4(\beta ,\kappa ) \sum _{e \in \gamma } \mu _{N,(\beta ,\kappa ),(\infty ,\kappa )}\bigl ({\mathcal {E}}_5(e)\bigr )} + 4\sqrt{2 \alpha _4(\beta ,\kappa ) \sum _{e \in \gamma } \mu _{N,(\beta ,\kappa ),(\infty ,\kappa )}\bigl ({\mathcal {E}}_4(e)\bigr )} \\  &\qquad + 2\sqrt{2 \alpha _4(\beta ,\kappa ) \sum _{e \in \gamma _c} \mu _{N,\infty ,\kappa }\bigl ({\mathcal {E}}_6(e)\bigr )} + 2\sqrt{2|{{\,\textrm{supp}\,}}\gamma _c| \alpha _3(\beta ,\kappa )} \\  &\qquad + 2\sqrt{2 \alpha _3(\beta ,\kappa ) \sum _{e \in \gamma } \mu _{N,(\beta ,\kappa ),(\infty ,\kappa )} \bigl ({\mathcal {E}}_7(e)\bigr )}. \end{aligned}$$

Inserting the upper bounds from Proposition 10.10, Proposition 10.11, Proposition 10.12, Proposition 10.13, Proposition 10.14, Proposition 10.15, and Proposition 10.16, and using the inequality $\sqrt{a+b} \le \sqrt{a} + \sqrt{b},$ we obtain (10.25) as desired. $\square$

### An upper bound

The following result generalizes [9, Lemma 7.12] and [17, Lemma 3.3], and is completely analogous to Lemma 12.3 in [18].

#### Proposition 10.19

[Lemma 12.3 in [18]]. Let $\beta ,\kappa \ge 0$, and let $\gamma \in C^1(B_N)$ be a path such that no edge in $\gamma$ is in the boundary of $B_N.$ Then

$$\begin{aligned} \Bigl |{\mathbb {E}}_{N,\beta ,\kappa } \bigl [ L_\gamma (\sigma ) \bigr ]\Bigr | \le \exp \bigl ( - |{{\,\textrm{supp}\,}}(\gamma -\gamma _c)| \, \alpha _5(\beta ,\kappa ) \bigr ). \end{aligned}$$

#### Remark 10.20

In Lemma 12.3 in [18], $\gamma$ is assumed to be a generalized loop, rather than a path. However, since the proof is identical in the two cases, we do not include a proof here.

### A proof of Theorem 10.1

In this section, we give a proof of Theorem 10.1. Before we give this proof, we recall the following lemma from [18].

#### Lemma 10.21

[Lemma 8.2 in [18]]. Let $\beta ,\kappa \ge 0$, and for each $g \in G$, let $j_g >0$ be given. Further, let $j \, {:}{=}\, \sum _{g \in G} j_g$. Then

$$\begin{aligned} \Bigl | \prod _{g \in G} \theta _{\beta ,\kappa }(g)^{j_g} \Bigr | \le e^{-j \alpha _5(\beta ,\kappa )}. \end{aligned}$$

#### Proof of Theorem 10.1

Let *N* be sufficiently large, so that ${{\,\textrm{dist}\,}}_0({{\,\textrm{supp}\,}}\gamma ,\partial C_1(B_N))\ge 8,$ and so that for each $e \in \gamma ,$ the support of ${\hat{\partial }} e$ contains no boundary plaquettes of $B_N.$

Then the assumptions of Proposition 10.18 holds, and hence

$$\begin{aligned} \begin{aligned}&\Bigl |{\mathbb {E}}_{N,\beta ,\kappa } \bigl [L_\gamma (\sigma )\bigr ]- {\mathbb {E}}_{N,\infty ,\kappa } \bigl [ L_\gamma (\sigma ) \bigr ] \Theta _{N,\beta ,\kappa }(\gamma ) \Bigr | \\  &\quad \le B \cdot 2|{{\,\textrm{supp}\,}}\gamma |\alpha _5(\beta ,\kappa ) + B' \cdot 2\sqrt{2|{{\,\textrm{supp}\,}}\gamma | \alpha _5(\beta ,\kappa )} \end{aligned} \end{aligned}$$

where

10.28 $$\begin{aligned} B {:}{=}\, K_{11}\alpha _2(\beta ,\kappa )^6/\alpha _5(\beta ,\kappa ) \quad \text {and} \quad B' {:}{=}K_{12} \sqrt{\alpha _2(\beta ,\kappa )^6/\alpha _5(\beta ,\kappa )}, \end{aligned}$$

where $K_{11}$ and $K_{12}$ are given in (10.26) and (10.26) respectively.

Using that for $x>0$, we have $x \le e^x,$ and $2\sqrt{x}\le e^x$, it follows that

10.29 $$\begin{aligned} \Bigl |{\mathbb {E}}_{N,\beta ,\kappa } \bigl [L_\gamma (\sigma )\bigr ]-{\mathbb {E}}_{N,\infty ,\kappa } \bigl [L_\gamma (\sigma )\bigr ] \Theta _{N,\beta ,\kappa } (\gamma ) \Bigr | \le (B+B') e^{2 |{{\,\textrm{supp}\,}}\gamma | \alpha _5(\beta ,\kappa ) }.\nonumber \\ \end{aligned}$$

Now recall that, by Proposition 10.19, we have 

By using the triangle inequality and applying Lemma 10.21, it follows that10.30Combining (10.29) and (10.30), we obtain

10.31 $$\begin{aligned} \begin{aligned}&\Bigl |{\mathbb {E}}_{N,\beta ,\kappa }[ L_\gamma (\sigma )] - {\mathbb {E}}_{N,\infty ,\kappa }[ L_\gamma (\sigma )] \Theta _{N,\beta ,\kappa }(\gamma ) \Bigr |^{1 + 2|{{\,\textrm{supp}\,}}\gamma |/|{{\,\textrm{supp}\,}}(\gamma -\gamma _c)|} \\  &\quad \le 2^{2|{{\,\textrm{supp}\,}}\gamma |/|{{\,\textrm{supp}\,}}(\gamma -\gamma _c)|}(B+B'). \end{aligned} \end{aligned}$$

Recalling Proposition 2.16 and Proposition 2.19, and letting $N \rightarrow \infty$, the desired conclusion thus follows from (10.31) after simplification. $\square$

### Simplifications for rectangular paths and $G = {\mathbb {Z}}_2$

The purpose of this section is to establish the tools we need in order make the small adjustments to the proof of Theorem 10.1 needed to instead obtain Theorem 1.1.

In order to simplify notations, for $\beta ,\kappa \ge 0$ and a path $\gamma ,$ we define

$$\begin{aligned} \Theta '_{N,\beta ,\kappa }(\gamma ) {:}{=}e^{-2|{{\,\textrm{supp}\,}}\gamma |e^{-24\beta -4\kappa }\bigl (1+(e^{8\kappa }-1)|{{\,\textrm{supp}\,}}\gamma |^{-1}\sum _{e \in \gamma }{\mathbb {E}}_{N,\infty ,\kappa }[\mathbb {1}_{\sigma (e)=1}]\bigr )}. \end{aligned}$$

The main result in this section is the following proposition.

#### Proposition 10.22

Let $\beta ,\kappa \ge 0$ be such that [A] and $6\beta >\kappa$ both hold, let $\gamma$ be a path along the boundary of a rectangle with side lengths $\ell _1$ and $\ell _2$ which is such that ${{\,\textrm{dist}\,}}_0({{\,\textrm{supp}\,}}\gamma ,\partial C_1(B_N))\ge 8,$ and let $G = {\mathbb {Z}}_2.$

Then10.32where10.3310.34$K_1$ is given by (6.2), $K_3$ and $K_4$ are given by (6.6).

The second result which we will state and prove in this section is the following proposition, which will be used to simplify the error term in the proof of Theorem 1.1.

#### Proposition 10.23

Let $\beta ,\kappa \ge 0$ be such that [A] holds, and let $\gamma$ be an open path along the boundary of a rectangle with side lengths $\ell _1$ and $\ell _2.$ Then there is a path $\gamma _0 \in C^1(B_N)$ such that $\partial \gamma _0 = -\partial \gamma$ andwhere $K_4$ is given by (6.6).

Before we give a proofs of Proposition 10.22 and Proposition 10.23, we will state and prove a few useful lemmas. For these lemmas, it will be useful to note that when $G = {\mathbb {Z}}_2$ and $\rho (G) = \{1,-1\}$, then

10.35 $$\begin{aligned} \theta _{\beta ,\kappa }(0) = \frac{1-e^{-24\beta -4\kappa }}{1+e^{-24\beta -4\kappa }} \quad \text {and} \quad \theta _{\beta ,\kappa }(1) = \frac{1-e^{-24\beta +4\kappa }}{1+e^{-24\beta +4\kappa }}. \end{aligned}$$

From this, it in particular follows that when $6\beta > \kappa ,$ then $\theta _{\beta ,\kappa }(0),\theta _{\beta ,\kappa } >0.$ Next, we recall from Section 12.2 in [18], that when $G = {\mathbb {Z}}_2$ and $\rho (G) = \{1,-1 \},$ we have

10.36 $$\begin{aligned} \begin{aligned}&\alpha _0(r) = \alpha _1(r) = \varphi _r(1)^2 = e^{-4r}, \qquad \alpha _2(\beta ,\kappa ) = e^{-4(\beta +\kappa /6)}, \\&\alpha _3(\beta ,\kappa ) = 1 - \theta _{\beta ,\kappa }(0) = \frac{ 2e^{-24\beta -4\kappa }}{1 + e^{-24\beta -4\kappa }}, \qquad \alpha _5(\beta ,\kappa ) = 1 - \theta _{\beta ,\kappa }(0) \\  &\qquad = \frac{ 2e^{-24\beta -4\kappa }}{1 + e^{-24\beta -4\kappa }}, \\&\alpha _4(\beta ,\kappa ) = \theta _{\beta ,\kappa }(0) - \theta _{\beta ,\kappa }(1) = \frac{2 e^{-24\beta } (e^{4\kappa }-e^{-4\kappa })}{(1 + e^{-24\beta -4\kappa })(1 + e^{-24\beta +4\kappa })}. \end{aligned} \end{aligned}$$

#### Proof of Proposition 10.23

Choose $\gamma _0$ so that $\gamma + \gamma _0$ is a generalized loop along the boundary of a rectangle with side lengths $\ell _1,\ell _2 \ge 2.$

**Fig. 7:**
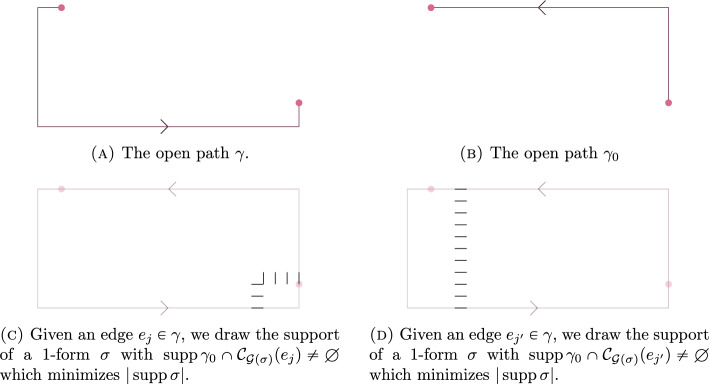
In the figures above, we illustrate the setting of the proof of Proposition 10.23. Note in particular that for the edge $e_j$ in 7a, we have ${{\,\textrm{dist}\,}}_1(e_j,{{\,\textrm{supp}\,}}\gamma _0) = |{{\,\textrm{supp}\,}}\gamma |-j+2,$ and for the edge $e_{j'}$ in 7b, we have ${{\,\textrm{dist}\,}}_1(e_{j'},{{\,\textrm{supp}\,}}\gamma _0) = \min (\ell _1,\ell _2)+1$

Let $e_1, e_2, \dots , e_{|{{\,\textrm{supp}\,}}\gamma |}$ be the edges in $\gamma ,$ labelled according to their order in the path $\gamma .$ Then, for any $j \in \{ 1,2, \dots , |{{\,\textrm{supp}\,}}\gamma |\},$ one verifies that (see Fig. 7)Using this inequality, we obtainEvaluating the geometric sum above, we obtain the desired conclusion. $\square$

We now proceed to the proof of Proposition 10.22. Before we give this proof, we will state and prove a few lemmas. To simplify the notation in these lemmas, when $6\beta > \kappa$ and $\sigma \sim \mu _{N,\infty ,\kappa },$ we define the following random variable.

10.37 $$\begin{aligned} \Upsilon _{\beta ,\kappa }(\gamma ) \, {:}{=}\, |{{\,\textrm{supp}\,}}\gamma |^{-1}\sum _{e \in \gamma } \log \theta _{\beta ,\kappa }\bigl (\sigma (e)\bigr ). \end{aligned}$$

#### Lemma 10.24

Let $\beta ,\kappa \ge 0$ be such that [A] and $6\beta > \kappa$ both hold, let $\gamma$ be path along a rectangle with side lengths $\ell _1$ and $\ell _2$ and such that ${{\,\textrm{dist}\,}}_0(\gamma ,\partial C_1(B_N))\ge 8,$ and let $G = {\mathbb {Z}}_2.$

Then, for any $\varepsilon > 0,$ we havewhere $K_{13}$ is given by (10.33).

#### Remark 10.25

The idea of the proof of Lemma 10.24 is essentially to use the weak law of large numbers for correlated random variables with exponential decay. For this to approach to work, we need the loop to be "smooth" enough for the sum of the covariances of all pairs of edges in $\gamma$ to be finite. The reason for working with rectangular loops is that in this case, it is relatively easy to show that this holds. However, with small modifications, the conclusion of this lemma holds for more general classes of loops as well, as long as the path $\gamma$ do not have too many corners.

#### Proof of Lemma 10.24

Fix some $$\varepsilon > 0.$$

By Chebyshev’s inequality, we haveBy combining Proposition 5.17, applied with $f_0 = \log \theta _{\beta ,\kappa }\bigl ( \sigma (e)\bigr )$ and $f_1 = \log \theta _{\beta ,\kappa }\bigl ( \sigma (e')\bigr ),$ and Proposition 6.4, it follows thatSince $0 \le \theta _{\beta ,\kappa }(1) \le \theta _{\beta ,\kappa }(0) \le 1$ for all $\beta ,\kappa \ge 0,$ we have

$$\begin{aligned} \begin{aligned}&\bigl \Vert \log \theta _{\beta ,\kappa } \bigr \Vert _\infty \le \bigl | \log \theta _{\beta ,\kappa }(1) \bigr | \le \bigl | \log \frac{1 - \varphi _\beta (1)^{12}\varphi _\kappa (1)^{-2}}{1 + \varphi _\beta (1)^{12}\varphi _\kappa (1)^{-2}}\bigr | \le \bigl | \log e^{ -2 \varphi _\beta (1)^{12}\varphi _\kappa (1)^{-2}} \bigr | \\&\qquad \le 2 \varphi _\beta (1)^{12}\varphi _\kappa (1)^{-2}. \end{aligned} \end{aligned}$$

Next, recall that, by Proposition (6.1), applied with $M = 1,$ $M' = 0,$ $\beta = \kappa _1 = \infty ,$ and $\kappa _2= \kappa ,$ for any edge $e \in \gamma ,$ we haveConsequently, for any $e \in \gamma ,$ we haveFinally, note that

$$\begin{aligned} \begin{aligned}&\sum _{e,e' \in {{\,\textrm{supp}\,}}\gamma :e \ne e'} (K_4\alpha _0(\kappa ))^{{{\,\textrm{dist}\,}}_0(e,e')} \le \sum _{e,e' \in {{\,\textrm{supp}\,}}\gamma _R :e \ne e'} (K_4\alpha _0(\kappa ))^{{{\,\textrm{dist}\,}}_0(e,e')} \\  &\qquad \le |{{\,\textrm{supp}\,}}\gamma | \Bigl ( |{{\,\textrm{supp}\,}}\gamma | /2 \cdot (K_4\alpha _0(\kappa ))^{\min (\ell _1,\ell _2)} + 2\sum _{j=1}^\infty (K_4\alpha _0(\kappa ))^{\min (j,8)} \Bigr ) \\  &\qquad \le |{{\,\textrm{supp}\,}}\gamma | \Bigl ( |{{\,\textrm{supp}\,}}\gamma | /2 \cdot (K_4 \alpha _0(\kappa ))^{\min (\ell _1,\ell _2)} + 16(K_4 \alpha _0(\kappa ))^{8} + \frac{2(K_4 \alpha _0(\kappa ))^9}{1-K_4 \alpha _0(\kappa )} \Bigr ). \end{aligned} \end{aligned}$$

Combining the above equations and recalling that when $G = {\mathbb {Z}}_2,$ we have $\alpha _0(\kappa ) = \varphi _\kappa (1)^2$ and $\alpha _2(\beta ,\kappa )^6 = \alpha _0(\beta )^6 \alpha _0(\kappa )^6 = \varphi _\beta (1)^{12}\varphi _\kappa (1)^{2},$ we finally obtainRearranging this equation, the desired conclusion now immediately follows. This concludes the proof. $\square$

#### Lemma 10.26

Let $\beta ,\kappa \ge 0$ be such that [A] and $6\beta >\kappa$ both hold, let $\gamma$ be path along the boundary of a rectangle with side lengths $\ell _1$ and $\ell _2,$ and let $G = {\mathbb {Z}}_2.$

Thenwhere $K_{13} = K_{13}(\ell _1,\ell _2)$ is given by (10.33).

#### Proof

Recall the definition of $\Upsilon _{\beta ,\kappa } (\gamma )$ from (10.37), and note that

$$\begin{aligned} \begin{aligned} \Theta _{N,\beta ,\kappa }(\gamma )&= {\mathbb {E}}_{N,\infty ,\kappa } \Bigl [ \prod _{e \in \gamma } \theta _{\beta ,\kappa }\bigl (\sigma (e)\bigr )\Bigr ] = {\mathbb {E}}_{N,\infty ,\kappa } \Bigl [ e^{\sum _{e \in \gamma } \log \theta _{\beta ,\kappa }\bigl (\sigma (e)\bigr )}\Bigr ] \\  &= {\mathbb {E}}_{N,\infty ,\kappa } \Bigl [ e^{|{{\,\textrm{supp}\,}}\gamma | \Upsilon _{\beta ,\kappa }(\gamma )}\Bigr ]. \end{aligned} \end{aligned}$$

Consequently,

$$\begin{aligned} \begin{aligned}&\biggl | Theta_{N,\beta ,\kappa }(\gamma ) - e^{|{{\,\textrm{supp}\,}}\gamma | {\mathbb {E}}_{N,\infty ,\kappa }[\Upsilon _{\beta ,\kappa }(\gamma )]} \biggr | \\  &\qquad = \biggl | {\mathbb {E}}_{N,\infty ,\kappa } \Bigl [ e^{|{{\,\textrm{supp}\,}}\gamma | \Upsilon _{\beta ,\kappa }(\gamma )} - e^{|{{\,\textrm{supp}\,}}\gamma | {\mathbb {E}}_{N,\infty ,\kappa }[\Upsilon _{\beta ,\kappa }(\gamma )]}\Bigr ] \biggr | \\  &\qquad \le {\mathbb {E}}_{N,\infty ,\kappa } \biggl [ \Bigl | e^{|{{\,\textrm{supp}\,}}\gamma | \Upsilon _{\beta ,\kappa }(\gamma )} - e^{|{{\,\textrm{supp}\,}}\gamma | {\mathbb {E}}_{N,\infty ,\kappa }[\Upsilon _{\beta ,\kappa }(\gamma )]}\Bigr | \biggr ]. \end{aligned} \end{aligned}$$

Next, note that since $\rho$ is unitary, we have $|\theta _{\beta ,\kappa }(g)|\le 1$ for all $g \in G,$ and hence $\Upsilon _{\beta ,\kappa }(\gamma ) \le 0.$ Now fix some $\varepsilon > 0.$

On the event $\bigl |\Upsilon _{\beta ,\kappa }(\gamma ) - {\mathbb {E}}_{N,\beta ,\kappa }[\Upsilon _{\beta ,\kappa }(\gamma ) ]\bigr |\ge \varepsilon ,$ since $\Upsilon _{\beta ,\kappa }(\gamma )\le 0,$ we must have

$$\begin{aligned} \Bigl | e^{|{{\,\textrm{supp}\,}}\gamma | \Upsilon _{\beta ,\kappa }(\gamma )} - e^{|{{\,\textrm{supp}\,}}\gamma | {\mathbb {E}}_{N,\infty ,\kappa }[\Upsilon _{\beta ,\kappa }(\gamma )]}\Bigr | \le 1. \end{aligned}$$

On the other hand, on the event $\bigl |\Upsilon _{\beta ,\kappa }(\gamma ) - {\mathbb {E}}_{N,\beta ,\kappa }[\Upsilon _{\beta ,\kappa }(\gamma ) ]\bigr |<\varepsilon ,$ since $\Upsilon _{\beta ,\kappa }(\gamma )\le 0,$ we have

$$\begin{aligned} \begin{aligned}&\Bigl | e^{|{{\,\textrm{supp}\,}}\gamma | \Upsilon _{\beta ,\kappa }(\gamma )} - e^{|{{\,\textrm{supp}\,}}\gamma | {\mathbb {E}}_{N,\infty ,\kappa }[\Upsilon _{\beta ,\kappa }(\gamma )]}\Bigr | \le \Bigl | \Upsilon _{\beta ,\kappa }(\gamma ) - {\mathbb {E}}_{N,\infty ,\kappa }[\Upsilon _{\beta ,\kappa }(\gamma )]\Bigr |< \varepsilon . \end{aligned} \end{aligned}$$

Using Lemma 10.24 with $\varepsilon = \bigl ( K_{13}\alpha _2(\beta ,\kappa )|{{\,\textrm{supp}\,}}\gamma |^{-1} \bigr )^{1/3},$ we obtain the desired conclusion. $\square$

#### Lemma 10.27

Let $\beta ,\kappa \ge 0$ be such that $6\beta > \kappa ,$ and let $G = {\mathbb {Z}}_2.$ Then

#### Proof

For the first inequality, note that

$$\begin{aligned} \begin{aligned}&\bigl | \theta _{\beta ,\kappa }(0) - e^{-2e^{-24\beta -4\kappa }} \bigr | = \bigl | \frac{1 - e^{-24\beta -4\kappa }}{1 + e^{-24\beta -4\kappa }} - e^{-2e^{-24\beta -4\kappa }} \bigr | \\  &\qquad \le \bigl | \frac{1 - e^{-24\beta -4\kappa }}{1 + e^{-24\beta -4\kappa }} - (1 - 2e^{-24\beta -4\kappa }) \bigr | + \bigl | (1 - 2e^{-24\beta -4\kappa }) - e^{-2e^{-24\beta -4\kappa }} \bigr ) \bigr | \\  &\qquad \le 2(e^{-24\beta -4\kappa })^2 + \bigl ( 2e^{-24\beta -4\kappa })^2/2 = 4(e^{-24\beta -4\kappa })^2. \end{aligned} \end{aligned}$$

The second inequality follows analogously. $\square$

#### Proof of Proposition 10.22

By definition, we haveConsequently, by Lemma 10.26,Next, by Lemma 10.6, we have

$$\begin{aligned} \begin{aligned}&\Bigl | \theta _{\beta ,\kappa }(0)^{\sum _{e \in \gamma } {\mathbb {E}}_{N,\infty ,\kappa } [ \mathbb {1}_{\sigma (e)=0} ]} \theta _{\beta ,\kappa }(1)^{\sum _{e \in \gamma } {\mathbb {E}}_{N,\infty ,\kappa } [ \mathbb {1}_{\sigma (e)=1} ]}\\&\qquad \qquad - e^{-2e^{-24\beta -4\kappa }\sum _{e \in \gamma }{\mathbb {E}}_{N,\infty ,\kappa }[\mathbb {1}_{\sigma (e)=0}]-2e^{-24\beta +4\kappa }\sum _{e \in \gamma }{\mathbb {E}}_{N,\infty ,\kappa }[\mathbb {1}_{\sigma (e)=1}]} \Bigr |\\&\qquad \le \sum _{e \in \gamma } {\mathbb {E}}_{N,\infty ,\kappa } [ \mathbb {1}_{\sigma (e)=0} ]\cdot \bigl | \theta _{\beta ,\kappa }(0) - e^{-2e^{-24-4\kappa }} \bigr | + \sum _{e \in \gamma } {\mathbb {E}}_{N,\infty ,\kappa } [ \mathbb {1}_{\sigma (e)=1} ]\cdot \bigl | \theta _{\beta ,\kappa }(1)\\&\qquad - e^{-2e^{-24+4\kappa }} \bigr |. \end{aligned} \end{aligned}$$

By combining Lemma 10.27 with Proposition 6.1, applied with $M = 1,$ $M' = 0,$ $\beta = \kappa _1 = \infty ,$ and $\kappa _2= \kappa ,$ we can bound the previous equation from above by

$$\begin{aligned} \begin{aligned}&|{{\,\textrm{supp}\,}}\gamma | \cdot 4(e^{-24\beta -4\kappa })^2 + K_1(\infty ,\kappa ) \bigl (K_4 \alpha _0(\kappa ) \bigr )^8 |{{\,\textrm{supp}\,}}\gamma | \cdot 4(e^{-24\beta +4\kappa })^2. \end{aligned} \end{aligned}$$

Combining the previous equations, we thus obtainRearranging this equation, and recalling from (10.36) that $e^{-24\beta -4\kappa } = \alpha _2(\beta ,\kappa )^6$ and $\alpha _0(\kappa ) = e^{-4\kappa },$ we obtain (10.32) as desired. $\square$

### A proof of Theorem 1.1

We now provide a proof of Theorem 1.1. Since this proof is very similar to the proof of Theorem 10.1, we will refer to this proof in order to avoid repetition.

#### Proof of Theorem 1.1

Let *N* be sufficiently large so that ${{\,\textrm{dist}\,}}_0(\gamma ,\partial B_N) \ge 8$ and so that for each $e \in \gamma ,$ ${\hat{\partial }} e$ contains no boundary plaquettes of $C_2(B_N).$

Using (10.35), it follows that if $\beta$ and $\kappa$ satisfy the assumptions of Theorem 1.1, then [A] hold.

By combining Propositions 10.18 and 10.32, using that  we obtain

$$\begin{aligned} \begin{aligned}&\Bigl |{\mathbb {E}}_{N,\beta ,\kappa } \bigl [L_\gamma (\sigma )\bigr ]- {\mathbb {E}}_{N,\infty ,\kappa } \bigl [ L_\gamma (\sigma ) \bigr ] \Theta '_{N,\beta ,\kappa }(\gamma ) \Bigr |\\&\quad \le \Bigl |{\mathbb {E}}_{N,\beta ,\kappa } \bigl [L_\gamma (\sigma )\bigr ]- {\mathbb {E}}_{N,\infty ,\kappa } \bigl [ L_\gamma (\sigma ) \bigr ] \Theta _{N,\beta ,\kappa }(\gamma ) \Bigr | + \Bigl | \Theta _{N,\beta ,\kappa }(\gamma ) - \Theta '_{N,\beta ,\kappa }(\gamma )\Bigr |\\&\quad \le \Bigl (B+\frac{K_{14} \alpha _2(\beta ,\kappa )^{12}}{2\alpha _5(\beta ,\kappa )}\Bigr ) \cdot 2|{{\,\textrm{supp}\,}}\gamma |\alpha _5(\beta ,\kappa ) + B' \cdot 2\sqrt{2|{{\,\textrm{supp}\,}}\gamma | \alpha _5(\beta ,\kappa )}\\&\qquad + \root 3 \of {\frac{K_{13}\alpha _2(\beta ,\kappa )}{|{{\,\textrm{supp}\,}}\gamma |^2 \alpha _5(\beta ,\kappa )}} \cdot 2\root 3 \of {|{{\,\textrm{supp}\,}}\gamma |\alpha _5(\beta ,\kappa )}, \end{aligned} \end{aligned}$$

where *B* and $B'$ are given in (10.28).

Using that for $x>0$, we have $x \le e^x,$ $2\sqrt{x}\le e^x,$ and $2\root 3 \of {x}\le e^x$, it follows that

$$\begin{aligned} \begin{aligned}&\Bigl |{\mathbb {E}}_{N,\beta ,\kappa } \bigl [L_\gamma (\sigma )\bigr ]-{\mathbb {E}}_{N,\infty ,\kappa } \bigl [L_\gamma (\sigma )\bigr ] \Theta '_{N,\beta ,\kappa }(\gamma ) \Bigr |\\&\qquad \le \Bigl (B + \frac{K_{14} \alpha _2(\beta ,\kappa )^{12}}{2\alpha _5(\beta ,\kappa )} + B' + \root 3 \of {\frac{K_{13}\alpha _2(\beta ,\kappa )}{|{{\,\textrm{supp}\,}}\gamma |^2 \alpha _5(\beta ,\kappa )}} \Bigr ) e^{2 |{{\,\textrm{supp}\,}}\gamma | \alpha _5(\beta ,\kappa ) }. \end{aligned} \end{aligned}$$

Combining this inequality with (10.30), we obtain

10.38 $$\begin{aligned}&\Bigl |{\mathbb {E}}_{N,\beta ,\kappa }[ L_\gamma (\sigma )] - {\mathbb {E}}_{N,\infty ,\kappa }[ L_\gamma (\sigma )] {\mathbb {E}}_{N,\infty ,\kappa } \Bigl [ \, \prod _{e \in \gamma } \theta _{\beta ,\kappa }(\sigma _e) \Bigr ] \Bigr |^{1 + 2|{{\,\textrm{supp}\,}}\gamma |/|{{\,\textrm{supp}\,}}(\gamma -\gamma _c)|}\nonumber \\&\qquad \le 2^{2|{{\,\textrm{supp}\,}}\gamma |/|{{\,\textrm{supp}\,}}(\gamma -\gamma _c)|} \Bigl (K_{11} + \frac{K_{14} \alpha _2(\beta ,\kappa )^{12}}{2\alpha _5(\beta ,\kappa )} + K_{12} + \root 3 \of {\frac{K_{13}\alpha _2(\beta ,\kappa )}{|{{\,\textrm{supp}\,}}\gamma |^2 \alpha _5(\beta ,\kappa )}} \Bigr ). \end{aligned}$$

Now recall (10.36), and note that these expression imply that

10.39 $$\begin{aligned} \frac{\alpha _2(\beta ,\kappa )^6}{\alpha _5(\beta ,\kappa )} \le 1 \quad \text {and} \quad \frac{ \alpha _4(\beta ,\kappa ) \alpha _0(\kappa )^2}{\alpha _5(\beta ,\kappa )} \le 1. \end{aligned}$$

Using these equations and inequalities and Proposition 10.23, it follows thatNow note that since $\gamma$ is a path along the boundary of a rectangle with side lengths $\ell _1,\ell _2 \ge 2,$ we must have $|{{\,\textrm{supp}\,}}\gamma _c| \le 8.$ Since [A] holds, we must have $2\alpha _0(\kappa ) \le K_4 \alpha _0(\kappa )\le 1,$ and since $G = {\mathbb {Z}}_2$, we have $\alpha _2(\beta ,\kappa ),\alpha _0(\beta )\le 1.$

Recalling Proposition 2.16 and Proposition 2.19, letting $N \rightarrow \infty ,$ and simplifying, we thus obtainwhere10.40Next, note that since $\gamma$ is a path along the boundary of some rectangle with side lengths $\ell _1,\ell _2 \ge 2,$ we have $|{{\,\textrm{supp}\,}}\gamma _c| \le 8.$ Since, by assumption, we have $|{{\,\textrm{supp}\,}}\gamma | \ge 24,,$ it follows that $|{{\,\textrm{supp}\,}}\gamma _c|/|{{\,\textrm{supp}\,}}\gamma | \le 1/3,$ and hence

$$\begin{aligned} \frac{1}{4} \le \frac{1}{1+2|{{\,\textrm{supp}\,}}\gamma |/(|{{\,\textrm{supp}\,}}\gamma |-|{{\,\textrm{supp}\,}}\gamma _c|)} \le \frac{1}{3}. \end{aligned}$$

If we in addition have $\alpha _2(\beta ,\kappa )+ \sqrt{\max (1,|{{\,\textrm{supp}\,}}\gamma _c|)/|{{\,\textrm{supp}\,}}\gamma |}\le 1$, then it follows that

10.41 $$\begin{aligned}  &   \Bigl |\bigl \langle L_\gamma (\sigma ,\phi ) \bigr \rangle _{\beta ,\kappa ,\infty } - \bigl \langle L_\gamma (\sigma ,\phi ) \bigr \rangle _{\infty ,\kappa ,\infty } \Theta '_{\beta ,\kappa }(\gamma ) \Bigr | \le 2^{1-\frac{1}{4}} \cdot K_{15}^{1/3}\nonumber \\    &   \quad \cdot \Bigl ( \alpha _2(\beta ,\kappa ) + \sqrt{1 /|{{\,\textrm{supp}\,}}\gamma |}\Bigr )^{\frac{1}{4}}. \end{aligned}$$

Since $|\rho (g)| = 1$ for all $g \in G$, we always haveConsequently, if $\alpha _2(\beta ,\kappa ) + \sqrt{|{{\,\textrm{supp}\,}}\gamma _c|/|{{\,\textrm{supp}\,}}\gamma |}\ge 1$, then (10.41) automatically holds. If we let

10.42 $$\begin{aligned} \begin{aligned}&K_0\, {:}{=}\, 2^{\frac{3}{4}} K_{15}^{1/3}, \end{aligned} \end{aligned}$$

we thus obtain (1.2). This completes the proof of Theorem 1.1. $\square$
